# Repurposing of Antimicrobial Agents for Cancer Therapy: What Do We Know?

**DOI:** 10.3390/cancers13133193

**Published:** 2021-06-26

**Authors:** Christina Pfab, Luisa Schnobrich, Samir Eldnasoury, André Gessner, Nahed El-Najjar

**Affiliations:** Institute of Clinical Microbiology and Hygiene, University Hospital Regensburg, 93053 Regensburg, Germany; Christina.Pfab@klinik.uni-regensburg.de (C.P.); Luisa.Schnobrich@klinik.uni-regensburg.de (L.S.); Samir.Eldanasoury@klinik.uni-regensburg.de (S.E.); Andre.Gessner@klinik.uni-regensburg.de (A.G.)

**Keywords:** repurposing drugs, oncology, antibiotics, off-targets, resistance, antivirals, antifungals, anthelmintic agents, antimalarial drugs

## Abstract

**Simple Summary:**

Despite the leaps and bounds in the prevention and treatment of cancer, it remains the second leading cause of death worldwide. A significant hurdle during cancer treatment is the occurrence of intrinsic and acquired therapeutic resistance. This latter case emphasizes the need for new drugs to overcome the challenges resulting from the current therapy. Considering that every drug has at least six off-targets, which might be relevant in cancer therapy, makes drug repurposing an excellent mean to speed up access to new therapeutic options. This review aims to thoroughly discuss anti-microbial agents currently in different trials or those evaluated in pre-clinical settings against various types of cancer and provide an overview of the mechanism(s) by which these agents exert their effects.

**Abstract:**

The substantial costs of clinical trials, the lengthy timelines of new drug discovery and development, along the high attrition rates underscore the need for alternative strategies for finding quickly suitable therapeutics agents. Given that most approved drugs possess more than one target tightly linked to other diseases, it encourages promptly testing these drugs in patients. Over the past decades, this has led to considerable attention for drug repurposing, which relies on identifying new uses for approved or investigational drugs outside the scope of the original medical indication. The known safety of approved drugs minimizes the possibility of failure for adverse toxicology, making them attractive de-risked compounds for new applications with potentially lower overall development costs and shorter development timelines. This latter case is an exciting opportunity, specifically in oncology, due to increased resistance towards the current therapies. Indeed, a large body of evidence shows that a wealth of non-cancer drugs has beneficial effects against cancer. Interestingly, 335 drugs are currently being evaluated in different clinical trials for their potential activities against various cancers (Redo database). This review aims to provide an extensive discussion about the anti-cancer activities exerted by antimicrobial agents and presents information about their mechanism(s) of action and stage of development/evaluation.

## 1. Introduction

High costs and long development times, high attrition rates, including failure in expensive late-stage clinical trials, and the rapid development of drug resistance, increase the financial burden on the pharmaceutical companies and unveil the limitations and challenges faced by traditional drug discovery [1,2,3,4,5]. This highlights the ever-increasing need for rapidly finding new affordable drugs. New uses of old drugs known as drug repurposing, drug repositioning, drug re-profiling, therapeutic switching, or indication switching rely on evaluating the effectiveness of currently approved drugs with known targets and reliable biomarkers against other illnesses [6,7]. During the past decade, interest in new uses for old medicines has grown among clinicians and researchers. From the day of its first appearance in 2005 [8,9] to date, more than 5000 hits were obtained in PUBMED only by searching the words “drug repurposing”. This is mainly because approximately 90% of approved drugs possess off-site targets that can be used to treat other illnesses [10,11]. Traditionally, drug’s off-targets are often considered responsible for toxic side effects; yet, although this is truthful in many cases, inadvertent target inhibition per se may elicit unexpected beneficial effects in a different disease context [5,12]. Such a drug thus constitutes an excellent candidate for drug repurposing if the off-target is inhibited potently enough and simultaneously is a potent disease driver. Besides, in the last decades, our understanding of the molecular and genetic bases of diseases has increased tremendously, and it is now acknowledged that many diseases share a molecular mechanism of pathogenesis. In this respect, the use of the same drug for more than one disease is also conceivable.

The most critical step in drug repurposing is identifying the right drug for an indication of interest. To overcome this obstacle, different assessment procedures, using computational and experimental approaches, are used excessively alone or in combination [3]. Although preset knowledge of the potential influence on common biological pathways or chemical structure of drugs allows the identification of repurposed drug candidates [5], historically, drug repurposing has mainly been opportunistic (based on side effects) and serendipitous. The most famous example of the former is sildenafil citrate, used initially to treat pulmonic hypertonia but is also found, following retrospective clinical experience, to be useful for erectile dysfunction [3,13]. Repurposing of thalidomide for erythema nodosum leprosum and multiple myeloma application, on the other hand, was based on serendipity [3,14].

### Repurposing Drugs in Oncology

The past years witnessed a revolution in our understanding of the molecular basis of cancer and the advancement of its prevention and therapy. Specifically, the concept of personalized medicine known as targeted therapy has emerged in the past two decades and resulted in the development of potent new-targeted drugs, which prolonged and improved the lives of many cancer patients [5,15,16,17,18]. This approach has been thought to cure cancer with little or no side effects and overcome the challenges faced by non-targeted therapies. Yet, unfortunately, only a few have curative potential among the wealth of the identified targeted drugs. To aggravate things further, the recurrent occurrence of resistance against anti-cancer drugs (targeted and non-targeted therapies) limits the duration of the clinical response and poses a significant pressure on the pharmaceutical companies to continuously search and invest in the discovery of new lead drugs [5,19,20,21]. 

**Figure 1:**
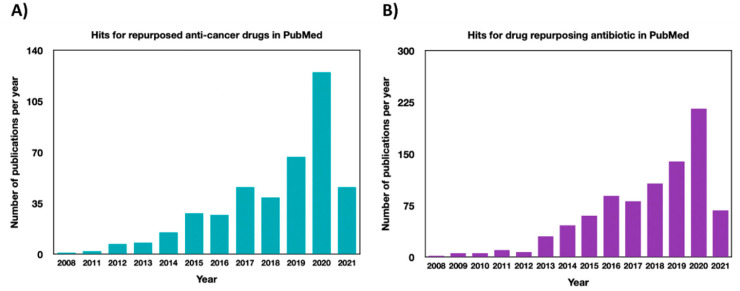
PubMed publications’ hits dated from 2008 for (**A**) “repurposed anti-cancer drugs” and (**B**) “drug repurposing antibiotic”.

Consequently, despite extensive resources and advances in cancer prevention and treatment, this ailment remains the second leading cause of mortality worldwide. This underscores the need for alternative efforts for cancer drug development. In the last years, attempts to use drug repurposing are increasing and encouraged by (1) the knowledge that cancer growth and metastases are orchestrated by multiple potential pathways and (2) the fact that most drugs have off-site targets, which are known anti-cancer targets in their own right [5,22]. Interestingly, more than 2000 drugs are approved worldwide, each of which has on average more than six relevant targets [23] that could be beneficial off-targets leading to quick, novel, safe, and cheap therapy. Subsequently, suppose logistic obstacles such as patent and regulatory considerations discussed extensively by Kato et al. and Pushpakon et al. [3,24] are overcome; the existing stock of drugs represents a reservoir of unexploited agents that could potentially make a clinically significant contribution in oncology. To date, there are a plethora of successful drug repurposing cases for many diseases, including oncology [5,25,26,27,28]. In this latter case, four repurposed drugs (thalidomide in multiple myeloma, retinoic acid in acute promyelocytic leukemia, zoledronic acid in solid cancer with bone metastases, NSAIDs in desmoid tumors) are already incorporated in the guidelines of the European Society for Medical Oncology (ESMO) or of the National Comprehensive Cancer Network (NCCN) [29]. Furthermore, much momentum is ongoing in pre-clinical and clinical settings to find new applications for other already approved non-cancer drugs. For instance, searching PUBMED for “repurposed anti-cancer drugs” has provided 376 hits, most of which are concentrated from 2015–2021 (Figure 1A). Surprisingly, in addition to anti-inflammatory, anti-diabetic, and anti-hypertension drugs, antimicrobial agents, thought to be only effective against prokaryotic cells, represent a rich source of agents with a wide range of off-targets in different diseases (Figure 1B). Therefore, in this review, light is shed on the current knowledge about diverse classes of approved antimicrobial drugs that are or can be repurposed in oncology. Different resources (the ReDo database, the Drug bank database, the national institute of health (NIH), ClinicalTrials.gov, google scholar, and PUBMED) have been used to identify potential repurposed antimicrobial candidates and their actual stand of investigation as anti-cancer drugs. The search covered approved repurposed drugs, drugs in clinical trials, or drugs that possess anti-cancer activities using in vitro and in vivo studies. 

## 2. Repurposing of Antimicrobial Agents 

**Figure 2:**
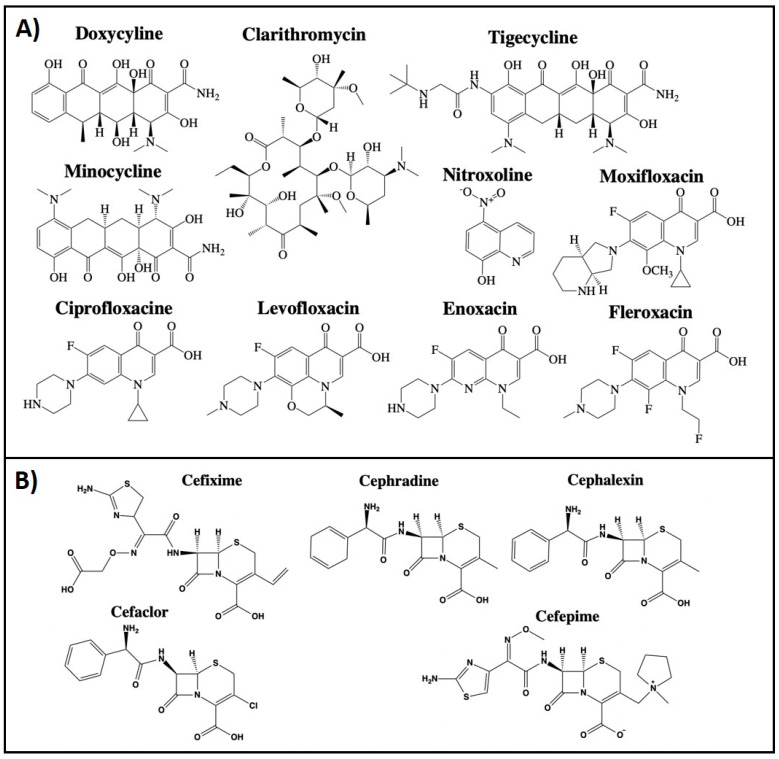
Structures of different classes of antibiotics with described off-site targets for cancer therapy. (**A**) Tetracyclin, Glycylcyclin, Fluoroquinolone, Makrolidantibiotic, and 8-Hydroxychinoline and (**B**) Cephalosporin’s antibiotics. Structures have been drawn with ChemDraw 20.0.

**Figure 3:**
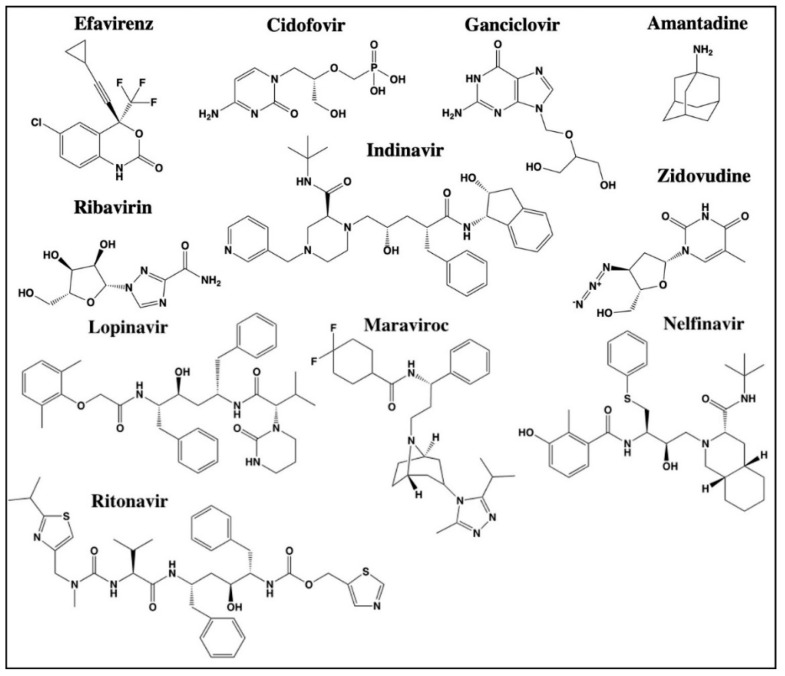
Structures of antiviral agents with reported off-site targets for cancer therapy (Efavirenz, Cidofovir, Ganciclovir, Ribavirin, Lopinavir, Ritonavir, Maraviroc, Nelfinavir, Zidovudine, and Indinavir) or detection (Amantadine). Structures have been drawn with ChemDraw 20.0.

**Figure 4:**
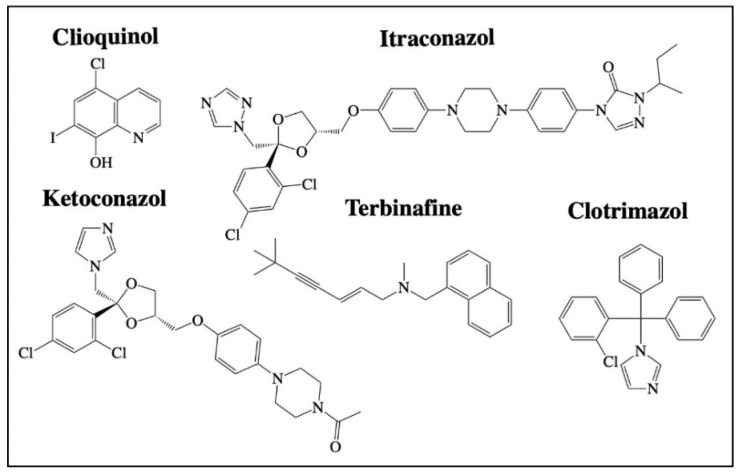
Structures of antifungals with reported off-site targets for cancer therapy. Structures have been drawn with ChemDraw 20.0.

**Figure 5:**
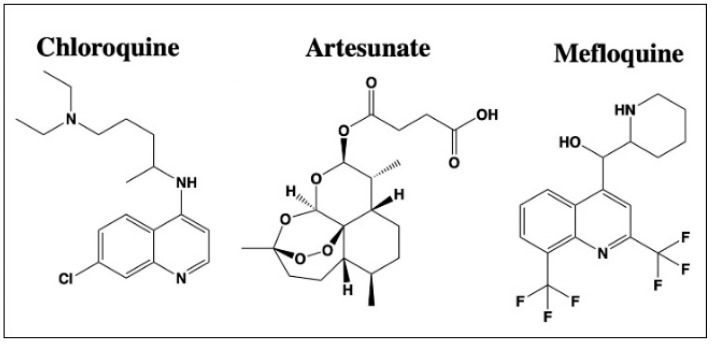
Structures of anti-malarial agents with reported off-site targets for cancer therapy. Structures have been drawn with ChemDraw 20.0.

**Figure 6:**
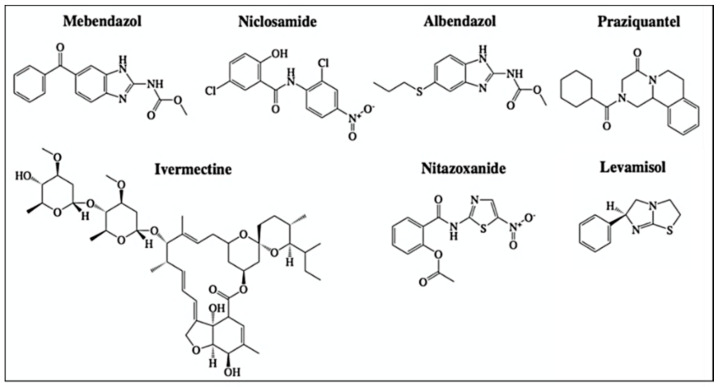
Structures of anthelmintic agents with reported off-site targets for cancer therapy. Structures have been drawn with ChemDraw 20.0.

Chemotherapy-treated cancer patients are immunosuppressed and therefore prone to infections [30,31]. Due to this reason, cancer patients often receive, in addition to chemotherapeutic agents, a multi-drug cocktail of antimicrobial agents to prevent or eradicate various infections [32,33]. Interestingly, it has been shown often in the past that some co-treatments (antimicrobial agents-chemotherapeutic agents) resulted in a higher survival rate or a better outcome in cancer patients in comparison to chemotherapy alone. Therefore, in the past years, extensive research has focused on (1) drugs enhancing the effectiveness of existing chemotherapeutic agents or (2) approved drugs with potent off-target sites in cancer. Consequently, in the followings the potentials of selected repurposed antimicrobial agents antibiotics (Figure 2), antifungals (Figure 3), antihelmintic (Figure 4), antivirals (Figure 5), and anti-malarial drugs (Figure 6) are thoroughly discussed in terms of their original application, off-targets against cancer, as well as their current status in repurposing in oncology. A more extensive summary is provided in Supplemental data Tables S1–S5.

### 2.1. Antibiotics

#### 2.1.1. Clarithromycin 

Clarithromycin is an FDA-approved drug sold under the name “BIAXIN” and is also available as a generic drug. Clarithromycin is a macrolide antibiotic active against Gram-positive and Gram-negative microorganisms. Through its binding to the bacterial 50S ribosomal subunit, it inhibits protein synthesis and hence arrests bacterial cell growth [34]. This antibiotic is used to treat infections of the respiratory system, i.e., pneumonia and bronchitis, as well as infections of the mouth, ear, nose, and throat [35]. Clarithromycin’s effect goes beyond its inhibitory effect against bacteria, whereas ample in vitro, in vivo, and clinical trial data is available about its beneficial effects against cancer (Supplemental Data, Table S1). The first clinical trial is dated back to 1997, when it proved effective against lung cancer [36]. Since then, a significant number of clinical trials have been performed or are still ongoing to test its effectiveness against different types of tumors such as Hodgkin lymphoma [37]. Nevertheless, although the most popular application of Clarithromycin is reducing tumor lung cell survival as a mono-treatment [38], it is much more effective when used in combination therapy. For instance, the cytotoxic effect of a combination of Bortezomib, a proteasome inhibitor, and Clarithromycin is more pronounced against melanoma when compared to the single treatment [39]. A similar effect has been observed with a combination of Clarithromycin and Carboplatin or Cyclophosphamide against adenocarcinoma [40], in myeloma when in combination with Thalidomide [41,42,43], and against breast cancer when in combination with Bortezomib [44] and Vorinostat [45]. Interestingly, Hamada et al. [38] showed that Clarithromycin treatment seven days post-chemotherapy with Vindesin sulfate and Cisplatin significantly enhances the activity of natural killer T-cells and CD8+ T cell cytotoxicity, caused a higher number of INF-ℽ- and IL-4-producing T cells leading to a better over-all outcome of the chemotherapy. Over the past years, extensive research shows that Clarithromycin induces its beneficial effects through inhibition of autophagy, caused by a prompted fusion of autophagosomes and lysosomes [41], apoptosis induction, and angiogenesis inhibition via inhibition of the endothelial cell tube formation [46], as well as metastasis reduction [47,48]. 

#### 2.1.2. Doxycycline 

Doxycycline is an FDA-approved broad-spectrum tetracycline antibiotic sold as a generic drug and under various brand names such as “Acticlate”, “Atridox,” or “Dorxy”. Doxycycline inhibits the protein synthesis in bacteria by binding to the 16S portion of the ribosomal RNA [49,50]. It is used to treat infections of the gastrointestinal and urinary tracts, the respiratory system, and the skin. Doxycycline is also used as a prophylaxis of malaria [50]. One characteristic of Doxycycline is its chelating properties of divalent cations such as Mg^2+^ and Ca^2+^ ions, which might affect their bioavailability in patients’ serum [51]. Screening the ClinicalTrials.gov (accessed on 5 April 2021) database provided many hits for clinical trials where Doxycycline is used to treat a wide range of cancers, including melanoma, different types of lymphoma, and prostate cancer, among others, by modulating different targets/ pathways as discussed hereafter (Supplemental Data, Table S1). Doxycycline inhibits cancer cells by blocking iNOS (nitric oxide synthase), essential for tumor development, growth, and angiogenesis [28,29,30]. In colon cancer cells, Doxycycline inhibits cancer cell growth by inducing G0/G1 arrest and acts as an inhibitor of matrix metalloproteinase, an effect that is potentiated when used in combination with COX-2 inhibitor [52]. Additionally, Doxycycline downregulates the proliferation and induces apoptosis of cervical cancer cells, affects oxygen consumption and glycolysis, and reduces ATP levels in vivo and in vitro [53]. In vitro studies on breast cancer cells show that Doxycycline inhibits cancer cell proliferation/viability and downregulates the expression of stem cell factors (Oct4, Sox2, Nanog, and CD44) and autophagy markers (LC-3BI and LC-3BII) [54,55]. Furthermore, Doxycycline reduces tumor-sphere formation in a dose-dependent manner in a large spectrum of cancer stem cells from the groups of breast cancer, ductal carcinoma in situ, ovarian cancer, prostate cancer, lung cancer, pancreatic cancer, melanoma, and glioblastoma. The mechanism by which Doxycycline induces its effect is through disruption of mitochondrial biogenesis [56]. This finding is a gain in cancer therapy as the observed effect is not cancer type-specific, and tumor formation could be reduced at an early stage. In addition, Doxycycline affects glycolysis, decreases mitochondrial activity, and blocks signaling through the STAT1/3, Sonic Hedgehog (Shh), Notch, WNT, and TGF-beta pathways in breast cancer cells [57]. In these latter ones, Lamb et al. [57] also describe a radio-sensitizing effect of Doxycycline by inhibiting the activity of DNA-PK, an enzyme that repairs DNA damage caused by irradiation. 

#### 2.1.3. Minocycline

Minocycline is another FDA-approved broad-spectrum tetracycline antibiotic active against Gram-negative and Gram-positive pathogens [58]. Minocycline is used to treat Lyme disease and infections of the urinary tract and of the skin (i.e., acne) [59]. It binds to the 30S subunit of the bacterial ribosome and inhibits protein synthesis in pathogens [58,60]. Minocycline is available under different names, “Arestin” or “Minocin“, as well as a generic drug. Ample in vitro and in vivo studies support the effectiveness of Minocycline alone in different types of cancer (Supplemental Data, Table S1). For instance, it has been shown that it inhibits ovarian cancer cells’ colony formation and proliferation by inducing cell cycle arrest, accompanied by the downregulation of cyclins A, B, and E. Minocycline affects ovarian cancer cells through the suppression of IL-6 expression, modulation of the IL-6 receptor system, and suppression of TGF-β1-TAK1-IκB signaling pathway [61,62,63]. On the other hand, in breast cancer, the combination of Minocycline and Celecoxib inhibits tumor cell growth, decreases the micro-vessel density, and lowers the expression of vascular endothelial growth factor (VEGF) and matrix metalloproteinase [64]. The latter enzyme is also inhibited in glioma cells treated with Minocycline, which induces its effect through autophagy induction [65,66]. Moreover, several clinical trials (e.g., NCT02297412, NCT01693523, and NCT02055963) are ongoing to test the ability of Minocycline to reduce side effects associated with chemotherapy as well as to increase the clinical outcome from the chemotherapy treatment of cancer-treated patients (ClinicalTrials.gov database (accessed on 5 April 2021), NIH).

#### 2.1.4. Tigecycline

Tigecycline is an FDA-approved broad-spectrum tetracycline antibiotic, available as a generic drug and also sold under the name “Tygacil”. Tigecycline is a reserve antibiotic used against multi-resistant Gram-positive and Gram-negative pathogens and should therefore not be used commonly for standard patients’ treatment [67]. In microorganisms, it inhibits protein synthesis through binding to the ribosomal subunit [68]. Tigecycline’s efficacy extends from inhibiting microbes to inhibiting cancer such as myeloid leukemia, glioma, non-small cell lung cancer, and retinoblastoma transcriptional corepressor 1 (RB1) negative breast cancer [69,70]. Due to its efficacy, it is in phase I trials for the treatment of acute myeloid leukemia. In vitro studies show that Tigecycline affects different cancer types through diverse mechanisms of action, including but not limited to inhibition of cell proliferation, migration/invasion [71], and angiogenesis [72], and induction of cell cycle arrest [71,73,74,75,76,77], oxidative stress [71,78,79], autophagy [73,77,80,81], and apoptosis [72,81,82,83,84]. Interestingly, Tigecycline is also active against triple-negative breast cancer that lacks the functions of the tumor-suppressor genes RB and TP53. These cells are characterized by an elevated ratio of mitochondrial protein translation associated with enhanced proliferation. Interestingly, Jones et al. [69] show, using in vitro and in vivo xenograft models, that Tigecycline inhibits mitochondrial protein translation and therefore is a potent inhibitor of RB deficient cells. A detailed description of the mechanism(s) by which Tigecycline affects mitochondrial gene expression and mitochondrial function in different cancer types is discussed extensively elsewhere [56,69,81,82,85,86,87]. Additional information about Tygecycline’s mechanisms of action is summarized in Supplemental Data, Table S1.

#### 2.1.5. Nitroxoline

Nitroxoline, a hydroxyquinoline derivative, is an approved antibiotic used to treat urinary tract infections caused by Gram-positive and Gram-negative bacteria as well as sprouts [88,89]. Nitroxoline is available under the brand name “Cysto-saar” but is also sold as a generic drug. A hallmark of its antimicrobial efficacy is related to its ability to chelate divalent cations, including but not limited to Zn^2+^, Mg^2+^, Ca^2+^, and Fe^2+^, which are essential for RNA synthesis and hence pathogens’ survival [90,91]. Sufficient in vitro and in vivo studies highlight the potential use of Nitroxoline as a repurposed drug for different cancer types [92,93,94,95,96] through various mechanisms of action (Supplemental Data, Table S1). For instance, Chang et al. [97] show that Nitroxoline inhibits prostate cancer cells by inducing cell cycle arrest, accompanied by a decrease in cyclin D1, Cdc25A, and phosphorylated Rb. Nitroxoline also increases AMP-activated protein kinase (AMPK) activity that inhibits the mTOR-p70S6K signaling pathway leading thus to an inhibition of cell proliferation [97]. On the other hand, in bladder and breast cancer, Nitroxoline inhibits cell proliferation via inhibition of MetAP2 activity and senescence induction. It also inhibits angiogenesis through inhibition of endothelial tube formation and reduction of micro-vessel density [92]. In other cell types such as lymphoma, leukemia, and pancreatic cancer, reactive oxygen species (ROS) generation is a hallmark of the Nitroxoline inhibitory effect that is further enhanced in the presence of copper as co-treatment [93].

#### 2.1.6. Cephalosporins

Cephalosporins, ß-lactam antibiotics, are commonly used broad-spectrum antibiotics against Gram-positive and Gram-negative bacteria [98,99,100,101,102,103,104]. The ß-lactam ring, characteristic of this class of antibiotics, is responsible for inhibiting bacterial cell wall synthesis and hence their effects. In vitro as well as in vivo studies show that cephalosporins (i.e., Cefepime, Cephalexin, Cefaclor, Cephradine, and Cefixime) (Supplemental Data, Table S1) are effective radio-sensitizer in breast, head, and neck cancer [105]. Among the cephalosporins mentioned above, only Cefepime, as a manganese complex, can inhibit breast tumor cell proliferation and colon cancer cells without irradiation [106]. 

#### 2.1.7. Fluoroquinolones

Fluoroquinolones are broad-spectrum antibiotic agents that inhibit the activity of bacterial gyrase and lead to a malfunction of DNA synthesis [107,108]. Several fluoroquinolones such as Ciprofloxacin, Moxifloxacin, Levofloxacin, Enoxacin, and Fleroxacin, among others, show potent anti-cancer activity (Supplemental Data, Table S1). Ciprofloxacin, for example, is active against different cancer types encompassing colon, bladder, prostate, osteoblastoma, osteosarcoma, neuroblastoma, and leukemia [109,110,111]. Ciprofloxacin induces mitochondrial damage, DNA fragmentation, and cell cycle arrest, as well as upregulates TGF-ß [109,112,113,114]. Enoxacin inhibits cell growth and induces cell cycle arrest and apoptosis of prostate, breast, and cervical cancer cells. Interestingly, Enoxacin is a cancer-specific modulator of miRNA biosynthesis [115,116,117,118]. Yadav and Talwar [119] summarize a wealth of data supporting the repurposing of fluoroquinolones as anti-cancer drugs alone, in combination (synergistic effect in the presence of 5-Fluorouracil per se), and as a metal-ion complex [119]. 

### 2.2. Antivirals

In addition to genetic abnormalities and unhealthy lifestyle, viral infections as the human papillomavirus (HPV), the human immunodeficiency virus (HIV), or the Epstein-Barr Virus can also be the basis for cancer development [120,121]. According to the WHO, 32 million people had HIV in 2019. The compromised immune system of HIV patients makes them prone to additional infections [121,122] and cancer development such as lymphomas and Kaposi’s Sarcoma [122]. Interestingly, beneficial effects against cancer therapy have been associated with antivirals used as a single- or combination-treatment in virally infected patients. Some of the most promising drugs are discussed hereafter. 

#### 2.2.1. Ganciclovir

Ganciclovir, an FDA-approved antiviral drug, is a structural analog to the nucleic base guanine and is used to treat Herpes Virus and Epstein - Barr virus-infected patients. Upon entry into infected host cells, Ganciclovir is further metabolized by viral kinases to its triphosphate analog. This latter one inhibits viral DNA polymerases and slows down the elongation of viral DNA [123]. Eastham et al. transduced a replicative-defective recombinant adenovirus (ADV) carrying the herpes simplex virus thymidine kinase gene (HSV-tk) into prostate cancer cells. A single treatment with Ganciclovir induced necrosis and apoptosis in tumor cells in vitro and in vivo [124,125]. Furthermore, 23 men with prostate cancer were subjected to a neoadjuvant HSV-tk gene therapy trial to sensitize the malignant cells to Ganciclovir. Briefly, an intraprostatic viral injection was performed, followed by Ganciclovir therapy. Interestingly, histological investigations of surgical specimens, taken two to four weeks after the viral injection, showed apoptosis induction and decreased micro-vessel density [125]. Similarly, ample in vitro and in vivo studies show promising antineoplastic results with HSV-TK transduced tumor cells, i.e., cervical cancer cells [126] or lung cancer cells [127]. This strategy is in clinical trials with different types of cancer (Supplemental Data, Table S2).

#### 2.2.2. Lopinavir

Lopinavir is an FDA-approved antiviral drug used to treat HIV-infected patients in antiretroviral combination therapy with Ritonavir. Lopinavir inhibits HIV-1 and HIV-2 proteases, which leads to a downregulation of the gag-pol-polyprotein cleavage resulting in immature non-infectious viruses [128,129,130,131]. Interestingly, combination therapy of Lopinavir and Ritonavir has been in focus for their potential use against cancer. For instance, this combination causes, in urological cancer cells, endoplasmic reticulum stress and increases the expression levels of AMP-activated protein kinase and tumor necrosis factor-related apoptosis-inducing ligand (TRAIL) receptor [132]. In lung cancer cells, the same combination induces cell cycle arrest, downregulates cell viability, and induces apoptosis [133]. Additionally, as a single treatment Lopinavir and its generated Lopinavir nitric oxide derivative inhibit cell proliferation, cause morphological changes, and induce reactive oxygen species production and apoptosis in melanoma [134].

#### 2.2.3. Indinavir

Indinavir, an FDA-approved antiviral drug sold under the name “Crixivan”, acts as an HIV protease inhibitor. Usually, Indinavir is prescribed to patients as a retro-viral combination treatment (e.g., with Ritonavir) to avoid resistance development [135,136,137,138]. In a programmed computer model, Indinavir has been found to modulate the alpha-7 nicotinic acetylcholine receptor (a pro-carcinogenic protein) and the matrix metalloproteinase-2 (MMP-2), a malignancy promotor [139]. In HPV-induced cervical cancer cells, Indinavir reduces cell viability, decreases the secretion of MMP-2 and MMP-9, and induces apoptosis. Additionally, Indinavir reduces tumor size in vivo [140]. Furthermore, in hepatocarcinoma, Indinavir also downregulates the MMP-2 proteolytic activation, inhibits angiogenesis, induces apoptosis, and delays tumor growth in vivo [141]. Similar effects were observed in Kaposi’s Sarcoma [142,143,144].

#### 2.2.4. Cidofovir

Cidofovir, an FDA-approved antiviral drug, inhibits the viral DNA polymerase and is used to treat cytomegalovirus (CMV) infections of AIDS patients [145]. The potential repurposing of Cidofovir is being investigated in vitro, in vivo, and in clinical trials. For instance, it has been shown that Cidofovir induces apoptosis in Glioblastoma [146], enhances the levels of p53 and p-pRb in cervical cancer infected with HPV [147,148], and is a potent radio-sensitizer [146,149].

#### 2.2.5. Efavirenz

Efavirenz, an FDA-approved antiviral drug sold under the name “Sustiva”, is given to HIV-1 infected people as an antiretroviral combination treatment, e.g., with Indinavir or Saquinavir. The antiviral activity of Efavirenz is due to its binding to the viral reverse transcriptase and hence downregulates the synthesis of the viral DNA [150,151]. Ample evidence supports the repurposing of Efavirenz for cancer therapy. In vitro, Efavirenz inhibits the progression of different cancer types, including colorectal, pancreatic [152,153], lung cancer [133], as well as to Glioblastoma [152], and leukemia [154]. Interestingly, this drug is not only effective as a single treatment but is also shown to synergize the effect of radiation therapy [155]. It is worth mentioning that the effect of Efavirenz, used at 600 mg, on prostate cancer in a clinical trial phase II has not shown a statistically significant result; yet, in some patients, a positive outcome on tumor suppression was seen [156]. Therefore, a higher dose is suggested for further trials. Other clinical trials with Efavirenz are listed in Supplemental Data, Table S2. One drawback of Efavirenz is its ability to bind with a high affinity to estrogen receptors, which might cause breast cancer [157]. 

#### 2.2.6. Maraviroc

Maraviroc, an antiretroviral drug, is the most prescribed C-C chemokine receptor type five (CCR5) co-receptor antagonist used to treat HIV infections. It interferes with the binding of C–C chemokine ligand 5 (CCL5) to CCR5, thus inhibiting the entry of the virus into the cell [158]. Ample in vivo and in vitro studies and one clinical trial (NCT01276236) show the potential of Maraviroc to treat cancer types typically associated with HIV infection, like Kaposi’s sarcoma and Lymphomas. However, accumulating evidence supports the effectiveness of Maraviroc against other tumor types, i.e., breast, colon, and gastric cancer, as well as different forms of leukemia [159,160]. Mechanistically, Maraviroc induces apoptosis in colorectal cancer [161], leads to cell cycle arrest in colorectal and pancreatic cancer [161,162], inhibits breast tumor metastasis [163], reduces monocyte accumulation in the tumor microenvironment of Lymphomas [164], and enhances the effect of a broad spectrum of drugs [159,163,165].

#### 2.2.7. Nelfinavir

Nelfinavir, an antiretroviral drug, is an HIV-1 protease inhibitor [166]. Several lines of evidence support the effectiveness of Nelfinavir against cancer. Mechanistically, it inhibits the proteasome activity in Glioblastoma, myeloma, laryngeal, ovarian, and lung cancer [167,168,169,170,171], leading to the accumulation of misfolded proteins, ER stress, and UPR response, which in turn results in apoptosis [167,168,169,170,171]. Furthermore, Nelfinavir inhibits HER2 protein expression and phosphorylation in breast cancer cells, as well as AKT and ERK1/2 signaling [172]. Nelfinavir also decreases MnSOD protein expression in cervical cancer and increases mitochondrial ROS production, leading to apoptosis and G1 cell cycle arrest [173]. Additionally, Nelfinavir synergizes the effect of several chemotherapeutic agents [168,169,174], showing its great potential to be repurposed for cancer treatment. Nelfinavir is currently under investigation in over 30 trials clinicaltrials.gov (accessed on 18 March 2021), listed in Supplemental Data, Table S2.

#### 2.2.8. Ritonavir

Ritonavir, similarly to Nelfinavir, is an antiretroviral drug used to inhibit the HIV-1 protease [175]. The potential of Ritonavir in cancer therapy is acknowledged. Ritonavir inhibits NF-κB activity, and the subsequent expression of NF-κB regulated gene products and cytokines in T-cell leukemia [176] and Kaposi’s Sarcoma [177], respectively. Additionally, it leads to G1 cell cycle arrest in breast, lung, and ovarian cancer [178,179,180,181]. The activity of Ritonavir is cell type-dependent, whereas it has been shown to activate and inhibit, respectively, in T-cell leukemia [182] and Glioma cells [78], the chymotrypsin-like activity of the proteasome. Ritonavir also synergizes the effects of many agents [178,180,181,183,184,185]. Ritonavir is in clinical trials phase I, II, and IV, often combined with other drugs such as Doxorubicin, Lopinavir, Metformin, and DS-8201a. Currently, there is only one clinical trial phase I where Ritonavir is used as monotherapy for breast cancer (Supplemental Data, Table S2).

#### 2.2.9. Ribavirin

Ribavirin is a broad-spectrum antiviral used in combination with Interferon-α to treat Hepatitis C via a mechanism that is not yet fully understood [186]. Ribavirin shows promising effects against cancer via suppressing eIF4E phosphorylation and activation and leading to decreased mRNA export and protein synthesis in pharyngeal, ovarian, breast cancer, acute lymphoblastic leukemia (ALL), osteosarcoma, and glioma [187,188,189,190,191,192,193,194]. Furthermore, Ribavirin modulates EZH2, Snail, and eIF4E in nasopharyngeal carcinoma (NPC), osteosarcoma, and glioma leading to decreased migration and adhesion [191,192,195]. Ribavirin also synergizes the effects of many chemotherapeutic agents [196] (Supplemental Data, Table S2). Ribavirin is currently being investigated alone in clinical trials phase I and II for AML, breast, head, and neck cancer and HCC and in combination with other drugs in two Phase IV clinical trials (Supplemental Data, Table S2).

#### 2.2.10. Zidovudine

Zidovudine, also known as Azidothymidine (AZT), an antiretroviral drug, is a thymidine analog that blocks chain elongation [197,198]. Zidovudine decreases telomerase activity in esophageal, colorectal, breast, parathyroid and ovarian cancer, as well as HCC [197,199,200,201,202]. It also induces mitochondrial dysfunction, increases oxidative stress [203], and synergizes the effects of many drugs [200,204,205] and irradiation [200,206]. Zidovudine is investigated in several clinical trials, i.e., phases I, II, and IV for the treatment of Kaposi’s Sarcoma and Lymphoma, and is in one clinical trial phase II against T-cell leukemia in combination with Interferon-α (Supplemental Data, Table S2). 

#### 2.2.11. Amantadine

Amantadine, an FDA-approved drug to treat symptoms of Parkinson’s disease [207,208,209], protects from Influenza A infection by blocking the viral M2 ion channel, preventing the entry of the virus into the host cell [207,210,211]. In opposition to the drugs mentioned above, Amantadine can be used for cancer detection but not therapy. Interestingly, Amantadine can be metabolized to N-acetylamantadine by spermidine/spermine N-acetyltransferase (SSAT), present in high levels in cancer cells only. N-acetylamantadine can be detected in urine and is considered a non-invasive, cheap, and fast detection method of cancer. Amantadine is currently in clinical phase II trial as a potential diagnostic biomarker for cancer detection. 

### 2.3. Antifungals

#### 2.3.1. Itraconazole

Itraconazole is an FDA-approved triazole antifungal drug primarily used orally to treat internal infestations. It eradicates fungal infection via inhibition of the ergosterol synthesis and hence membrane synthesis [212]. A wealth of in vitro and in vivo studies [213] show that Itraconazole eradicates not only fungal infestations but also has promising anti-cancer effects (Supplemental Data, Table S3). For example, Itraconazole inhibits tumor cell growth, cell proliferation, and colony formation in melanoma. The observed effects are due to a downregulation of the expression of Gli-1, Gli-2, Wnt3A, β-catenin, and cyclin D1, which result in an obstruction of the Hedgehog and WNT signaling pathway. Furthermore, Itraconazole also inhibits the phosphorylation of p70S6K, 4E-BP1, and AKT and hence suppresses the PI3K/mTOR signaling pathway [214]. In pancreatic and ovarian cancers, Itraconazole, in combination with chemotherapeutic drugs, significantly enhances the overall survival of patients [213,215] through modulation of the Hedgehog signaling pathway and the inhibition of angiogenesis by its triazole unit [215,216]. Mechanistic analysis using in vivo and in vitro studies with endothelial cells show that Itraconazole restrains their progression, induces cell cycle arrest, and blocks the endothelial growth factor, which leads to an inhibition of angiogenesis [217]. Currently, it is in clinical phase I and II trials against different cancers (i.e., prostate, lung, and Esophageal).

#### 2.3.2. Ketoconazole

Ketoconazole is an antifungal drug used to treat infections of the skin as foot fungus. It is supplemented in special creams or shampoos. Ketoconazole inhibits the synthesis of fungals’ ergosterol and damages the cell membrane [218]. Another application of Ketoconazole is against Cushing-Syndrome, where it inhibits steroid and cholesterol synthesis in the adrenal gland by inhibiting the cytochrome P450 system [219,220,221]. Through its anti-androgen properties [222], Ketoconazole is also active against prostate cancer [223]. Despite the promising results of Ketoconazole displayed at high doses in clinical trials, unfortunately, its use is limited due to gastrointestinal side-effects [224]. Nevertheless, further clinical trials are ongoing to evaluate its effects against prostate and breast cancer (Supplemental Data, Table S3). 

#### 2.3.3. Clioquinol

Clioquinol is an antiseptic agent used for external infections caused by fungi or bacteria. It is commonly used for skin applications and therefore is added to ointments intended for wound-healing or neurodermatitis. The antimicrobial action of Clioquinol is due to its ability to bind to sulfhydryl-groups, which leads to an inhibition of essential enzymes of the pathogens. Furthermore, its ability to chelate divalent cations as Zn^2+^ and Cu^2+^ made it an attractive drug to be repurposed for Alzheimer’s disease, characterized by an overload ratio of these cations [225,226]. Clioquinol also shows promising effects against various cancer cells and already in phase I clinical trial against leukemia (Supplemental Data, Table S3). Its mechanism of action against tumor cells is mainly caused by its properties to act as a Zn^2+^ ionophore. Briefly, treatment of cancer cells with a combination of Clioquinol and zinc enhances zinc’s level inside the cells, leading to a disruption of lysosome integrity followed by cleavage of Bid and apoptosis induction. Apart from targeting lysosomes, Clioquinol independently inhibits the activity of NF-κB [225,227,228,229].

#### 2.3.4. Clotrimazole

Clotrimazole is an FDA-approved broad-spectrum anti-mycotic drug used to treat fungal infections of the skin and vagina. It is often applied as a solution or cream. It affects the ergosterol synthesis of the fungus, which leads to an inhibition of fungal cell growth and cell wall permeability [230,231]. Promising in vivo and in vitro data is available about the anticancer activity of Clotrimazole against different types of cancer such as breast, melanoma, colon, and lung tissues (Supplemental Data, Table S3). The mechanism by which Clotrimazole induces its effect is through the detachment, from the cytoskeleton, of specific glycolytic enzymes such as hexokinase, aldolase, or fructokinase [232,233,234,235,236]. Kadavalkollu et al. suggest that Ca^2+^-activated potassium channels also mediate Clotrimazol-induced anticancer activity [237]. Furthermore, Labay et al. [105] report, in vivo, a radio-sensitizing effect of Clotrimazol in melanoma. 

#### 2.3.5. Terbinafine

Terbinafine is an FDA-approved broad-spectrum anti-mycotic agent used to treat fungal colonization of nails and on the skin. It is applied as cream, gel, solution, or spray but can also be taken orally. This drug inhibits non-competitively the squalene oxidase, which by the inhibition of the ergosterol synthesis, affects cell wall formation of the fungus [238,239]. Labay et al. [105] report a radio-sensitizing effect of Terbafine in an in vivo model using murine melanoma (Supplemental Data, Table S3).

### 2.4. Anti-Malarial Drugs 

In addition to the afore-discussed drugs, three anti-malarial drugs (Chloroquine, Artesunate, and Mefloquine) show strong potential to be repurposed as anti-cancer drugs. Chloroquine, an FDA-approved drug invented to treat malaria, shows significant activity against different illnesses such as lupus, rheumatic arthritis, discoid [240], Covid-19 infections [240,241,242,243,244,245,246,247], and cancer [248] (Supplemental Data, Table S4). For instance, in glioblastoma, Chloroquine delays tumor growth in epidermal growth factor receptor overexpressing glioblastoma xenografts, inhibits autophagy and is a potent radio-sensitizer [249]. In combination with gold-standard chemotherapeutic agents, Chloroquine exerts a synergistic effect on the overall outcome. In breast cancer cells, on the other hand, Chloroquine activates p53 and sensitizes the tumors to PI3K/AKT inhibitors [250]. Artesunate, used to treat malaria, induces its effects by cleaving endoperoxide bridges, inducing ROS generation in the parasite, and DNA double-strand breaks formation [251]. Significant in vivo and in vitro data support the beneficial effects of Artesunate against cancer (Supplemental Data, Table S4). For example, in T-cell leukemia, Artesunate induces ROS generation and apoptosis, associated with cytochrome C release and caspase-9 cleavage [252]. Furthermore, Artesunate inhibits angiogenesis and tumor growth in Kaposi’s sarcoma [253]. In renal cell carcinoma, Artesunate inhibits cell proliferation, migration as well as metastasis, and angiogenesis via decreased phosphorylation of the VEGFR2 [254]. Hamacher-Brady et al. [255] show that Artesunate disrupts the endo-lysosomal trafficking and inhibits the autophagic flux in breast cancer. Artesunate is not only active against cancer as a mono-treatment but also radio-sensitizes lung cancer cells to radiation [256] and synergizes the effect of many drugs such as Sorafenib [257], Doxorubicin, Paclitaxel [258], and Cisplatin [259]. Mefloquine, an anti-malarial drug with not a well-defined mechanism of action, is shown to target the 80S-ribosomal subunit of the parasite [260], which might affect protein synthesis. In addition to its original application, Mefloquine potentiated the effect of many drugs against different cancer types (Supplemental Data, Table S4). For instance, when combined with Doxorubicin and Paclitaxel a synergism and chemo-sensitization effects are observed in breast cancer cells [258], leukemia [261], gastric cancer [262], cervical cancer [263], and colon cancer [264]. This effect is caused by a decreased P-gp expression, which reduces the activity of the efflux pump, as well as by its ability to decreases cell proliferation and self-renewal. As a single-treatment in leukemia cells, Mefloquine disrupts lysosomes via ROS production, releases cathepsins, and decreases proliferation leading to enhanced cell death [265]. In breast cancer cells, on the other hand, Mefloquine arrests autophagy at the stage of autophagosome formation via increased LC3 and p62 expression [266]. Liu et al. [262] further show that Mefloquine inhibits gastric cancer by inhibiting the PI3K/Akt/mTOR phosphorylation and signaling. 

### 2.5. Anthelmintic Agents

#### 2.5.1. Mebendazole, Niclosamide, Albendazole, and Ivermectin 

Mebendazole, Niclosamide, Albendazole, and Ivermectin are standard anthelmintic drugs. The latter two are FDA-approved. All are used to treat worm infections via different mechanisms of action. Ivermectin induces its effect via modulation of glutamate-gated ion channels and γ-aminobutyric acid receptors [267,268,269], while Mebendazole and Albendazole bind to tubulin and inhibit tubulin formation in the worms [270]. Niclosamide, on the other hand, affects the glucose uptake by the worm leading to starvation [271]. All the aforementioned drugs show great potential for cancer treatment in vitro as well as in vivo studies and are already tested for their effectiveness in different clinical trials [61,272,273,274,275,276,277,278,279,280,281,282,283,284,285,286,287] (Supplemental Data, Table S5). Mechanistically, Mebendazole inhibits colony formation, reduces angiogenesis, and induces cytotoxicity in meningioma cells via caspase-3 activation. The anti-tumor effects of Mebendazole are enhanced by radiation [282]. In medulla blastoma, Mebendazole suppresses the primary cilium formation, a tubulin-based organelle that is a signaling hub for the Hedgehog pathway leading thus to reduced cell growth [286]. Interestingly, Mebendazole also stimulates the immune system, which through stimulation of CD14+ myeloid cells leads to an enhanced T-cell activation resulting in a stronger ability to kill tumor cells [285]. Additionally, Mebendazol increases the levels of TNF-α and IFN-γ [285]. Albendazole inhibits colony formation and cell proliferation of ovarian cancer cells [287] and disrupts tubulin formation, resulting in the inhibition of tumor cell growth and angiogenesis [288]. Due to its poor aqueous solubility, it has been tested in vivo as a BSA-Albendazole nanoparticle. These later ones show an enhancement of its inhibitory effect on vascular endothelial growth factor [289]. The antineoplastic effect of Albendazole against advanced malignancies has been confirmed in clinical phase I trial [290]. At the moment, it is in a clinical phase II trial to treat neoplasms. Niclosamide has different effects against different types of tumors. For instance, in adrenocortical carcinoma cells, Niclosamide restrains tumor growth and proliferation, induces cell cycle arrest and apoptosis, and reduces cellular migration [291]. In renal cancer cells, it inhibits C-MYC and E2F1 expression and enhances the expression of PTEN, thus leading to an inhibition of cell proliferation, migration, and progression. In lung cancer, it acts as a radio-sensitizer [292]. Furthermore, a synergizing effect is seen when Niclosamide has been used in combination with Sorafenib, a protein kinase inhibitor [293].

#### 2.5.2. Ivermectin

Ivermectin, an anthelmintic drug active against ectoparasites and threadworms, is used in various applications for humans and animals and to protect crops in agriculture [267]. Many in vivo and in vitro data highlight the potential of Ivermectin as an anti-cancer agent against a wide range of cancers (Supplemental Data, Table S5). For example, in ovarian cancer, Ivermectine causes DNA damage through induction of double-strand DNA breaks and induces intrinsic apoptosis by disrupting the mitochondrial membrane, associated with upregulation of BAX/BCL-2 and cytochrome C release [268]. Similar effects are seen in Ivermectin-treated liver cancer [269]. Interestingly, Ivermectin induces cell death in leukemia via a different mechanism of action, such as increasing the intracellular level of chloride ions via plasma-membrane hyperpolarization and increasing ROS levels [273]. Furthermore, Ivermectin inhibits cell proliferation and induces apoptosis in colon cancer cells by blocking the canonical WNT pathway, only on TCF-dependent cell types [276]. In glioblastoma, Ivermectin inhibits angiogenesis and deactivates the Akt/mTOR signaling pathway following mitochondrial stress and enhanced ROS levels [277]. Like glioblastoma but with an additional downregulation of self-renewal transcription factors, Ivermectin inhibits the growth of stem-like breast cancer cells [294]. In vivo and in vitro studies confirming the anti-cancer properties of the above-mentioned anthelmintic drugs are thoroughly discussed and summarized in the review by Hamilton and Rath [284]. Ivermectin is currently in clinical phase II trial against neoplasms (Supplemental Data, Table S5).

#### 2.5.3. Nitazoxanide

Nitazoxanide is an FDA-approved anti-microbial agent, developed initially as an anthelmintic agent due to its inhibitory effect on pyruvate-ferredoxin-oxidoreductase, essential for the anaerobic metabolism of parasites [295,296]. Nitazoxanide also shows significant activity against cancer (Supplemental Data, Table S5). For instance, treating colon cancer cells with Nitazoxanide leads to an inhibition of cell growth, nuclear condensation, DNA fragmentation, and apoptosis induction, at which glutathione-S-transferase P1 (GSTP1) is described as its primary target [297]. Furthermore, it activates the AMPK pathway and downregulates the c-Myc, mTOR, and WNT signaling in colon cancer [298]. In glioblastoma, Nitazoxanide inhibits autophagy regulation by blocking the late-stage lysosome acidification, suppressing cell growth, and inducing cell cycle arrest by upregulating the expression of inhibitor growth protein 1 (ING1) [299]. In breast cancer cells, it inhibits the expression of c-Myc, which leads to suppression of tumor growth, and apoptosis induction [300]. Additional anti-cancer properties of Nitazoxanide are extensively discussed in the review by Di Santo and Ehrisman [301]. 

#### 2.5.4. Praziquantel

Praziquantel is an FDA-approved anti-parasite drug with not yet a defined mechanism of action [302]. Nevertheless, Thomas and Timson [303] postulate potential targets that synergize to produce the overall pharmacological outcome. One confirmed mechanism is that Praziquantel causes spastic paralysis of the worm musculature related to a fast Ca^2+^ influx inside the worm [302,304]. In addition to its original application, Praziquantel shows promising anti-cancer activity both in vitro and in vivo and is currently used in a clinical trial to treat neoplasms (Supplemental Data, Table S5). Hua Wu et al. [305] show that when in combination with Paclitaxel, Praziquantel potentiates cell growth inhibition by downregulating the apoptosis protein XIAP in colon (DLD-1) and lung (H1299) cancer cells.

#### 2.5.5. Levamisole

Levamisole is used to treat worm infections by binding to the nicotinic acetylcholine receptors, which inhibit the reproduction of the male worms [306,307,308]. Furthermore, Levamisole stimulates or suppresses in humans the immune system, depending on the applied concentration and the timing of application [309,310]. Levamisole also shows potent activity against lung cancer, melanoma, and myeloma, in vivo and in vitro [311,312]. For instance, Levamisole inhibits myeloma cells by inducing the loss of CD138, a transmembrane heparin sulfate glycoprotein enhanced in malignant cells and by increasing IL-6 secretion [311]. In lung cancer cells, it inhibits cJNK phosphorylation and induces cell cycle arrest and tumor necrosis factor-related apoptosis [312]. Alone, Levamisole is in clinical trials to treat neoplasms (Supplemental Data, Table S5). Besides, many trials are ongoing to test its efficacy when combined with other standard chemotherapeutic agents such as 5-fluorouracil to treat colon cancer (NCT00002593, NCT00425152, NCT00002551, NCT00309530, NCT00003063) (Supplemental Data, Table S5). 

#### 2.5.6. Pyrvinium

Pyrvinium is used to treat worm infestations of the human intestinal tract with tapeworm per se. Its mechanism of action relies on inhibiting glucose uptake by the worm, which leads to starvation [284]. A Large number of in vivo and in vitro studies support its activity against cancer cells (Supplemental Data, Table S5). For instance, Pyrvinium inhibits cell proliferation and tumor cell renewal in colon [313] and breast [314] cancers. It has been seen that Pyrvinium affects the WNT signal transduction pathway via downregulation of the mRNA transcription or the protein expression of known WNT targets [313]. Furthermore, Pyrvinium also inhibits autophagy in various cancer cells (HeLa, HeLa-GFP-LC3, HEK293, PANC1, HCT-116, and MEF). In breast cancer stem-like cells, Pyrvinium impedes lipid anabolism and impairs the anabolic flux from glucose to cholesterol and fatty acids [315]. Moreover, Pyrvinium disrupts mitochondrial biogenesis [56] and intercalates into DNA [316]. Interestingly, Pyrvinium is more toxic to cancer cells when combined with the anti-cancer and autophagy stimulating agent 2-deoxy-D-glucose [317] and with 5-fluorouracil in colon cancer cells [313]. 

## 3. Drug Repurposing Pros and Cons

Several advantages result from repurposing clinically used drugs over developing an entirely new drug for a given indication [11,318]. On the one hand, the risk of failure is lower, at least from a safety point of view; on the other hand, information about their toxicity, posology, and formulation development is already available, which will significantly lower the costs needed for the drugs to reach the patients [2,3,5,11,318]. Indeed, the costs of bringing a repurposed drug to the market are ten times lower than the costs estimated for new chemical entities [3,319]. Furthermore, not only are the costs lower, but also the development time is decreased due to the wealth of pre-existing knowledge obtained from phase I clinical trial, i.e., pharmacokinetics and bioavailability [27]. For instance, compared to normal drug development that takes 10-17 years, the development of repurposed drugs needs a range of 3-12 years [3,14]. This is further facilitated by the faster processing time by the required authorities such as the FDA to review drug applications and less stringent requirements for approval of secondary indication of a drug, as the FDA often does not require duplication of drug safety assessments per se [10]. Yet, it is worth mentioning that despite the many advantages of repurposing drugs, failure in the later stage (phase II or III trials) is similarly expected, as encountered with a new chemical entity; however, it is unlikely to occur due to toxicity but rather due to lack of significant potency [1,5,320,321]. It is noteworthy that even if the safety profile of the repurposed drug is already defined, it is often that its recommended dose for treating cancer is much higher than the dose required for its original application, which might result in undesired side effects. Therefore, in many cases, the identification of anti-cancer activity for a non-cancer drug does not omit the necessity of conducting Phase I trials to establish the safety profile of the repurposed drug at the relevant anticancer concentration. Altogether, this might affect the incentive of the pharmaceutical industry to fund the randomized trials that would show/confirm the effectiveness of generic and inexpensive repurposed drugs. Besides, additional barriers such as patent considerations, regulatory considerations, and organizational hurdles limit adopting a repurposed drug; this is discussed extensively elsewhere [3,24]. 

## 4. Conclusions and Future Prospects

In 2020, 4.8 million people were diagnosed with cancer, reflecting the discovery of 13,000 cases/day or 9 cases/minute [322]. The alarming increase in cancer cases and the challenges and failure of current therapy increase the need for new approaches to treating this devastating illness. Re-evaluating the activity of approved drugs towards new uses outside the scope of their medical application stems from previous knowledge of their biological activities on disease targets or their off-sites targets. This is further encouraged by their known safety profile, as they have passed a substantial number of toxicity tests, which will minimize the possibility of failure for reasons of adverse toxicology. In the case of off-sites targets, it is crucial to understand the biological context of the respective off-target and its impact on the specific disease before testing it in clinical trials. Furthermore, toxicity tests should also be repeated if the drug is effective at a much higher concentration than its original application. Considering that anticancer drugs are costly, which poses an enormous burden on the health system, discovering anticancer activities from existing generic drugs, which are often much cheaper, is a better alternative for cancer therapy, even in low-income countries. Despite that many non-cancer drugs are currently being investigated against different types of cancer in preclinical and clinical settings, only a handful of drugs are approved. Therefore, more efforts should be made to overcome obstacles, such as patent and regulatory considerations, and encourage the different authorities to adopt and fund the repurposing of drugs, as this strategy offers an exciting opportunity for increasing the number of available anticancer therapies rapidly. Collectively, this review provides an overview of the wide variety of off-targets modulated by antimicrobial agents and highlights their potentials as cheap and potent drugs to be used in the future to treat various cancer types alone or in combination with chemotherapy. 

## Supplementary Materials

The following are available online at https://www.mdpi.com/article/10.3390/cancers13133193/s1, Table S1: List of antibiotic agents that are currently being investigated in clinical trials for their effectiveness against different cancer types when alone and/or in combination with chemotherapeutic agents, as well as antibiotics that are potential new anti-cancer drugs shown to inhibit malignant cells in vivo/in vitro. Table S2: List of antiviral agents that are currently being investigated in clinical trials for their effectiveness against different cancer types when alone and/or in combination with chemotherapeutic agents, as well as antivirals that are potential new anti-cancer drugs shown to inhibit malignant cells in vivo/in vitro. Table S3: List of antifungal agents that are currently being investigated in clinical trials for their effectiveness against different cancer types when alone and/or in combination with chemotherapeutic agents as well as antifungals that are potential new anti-cancer drugs shown to inhibit malignant cells in vivo/in vitro. Table S4: List of anti-malarial agents that are currently being investigated in clinical trials for their effectiveness against different cancer types when alone and/or in combination with chemotherapeutic agents. Table S5. List of anthelmintic agents that are currently being investigated in clinical trials for their effectiveness against different cancer types when alone and/or in combination with chemotherapeutic agents as well as anthelmintics that are potential new anti-cancer drugs shown to inhibit malignant cells in vivo/in vitro.
