# Supplementary material for: Repurposing of Antimicrobial Agents for Cancer Therapy: What Do We Know?
Source: Cancers (Basel). 2021 Jun 26;13(13):3193. doi: 10.3390/cancers13133193 (PMC8269327; doi:10.3390/cancers13133193)
Supplement: Supplementary file 1 [file cancers-13-03193-s001.zip › Supplemental Data Table S1.pdf]

Repurposing of Antimicrobial Agents for Cancer Therapy: What Do We Know?

Christina Pfab, Luisa Schnobrich, Samir Eldnasoury, André Gessner and Nahed El-Najjar

Table S1. List of antibiotic agents that are currently being investigated in clinical trials for their effectiveness against different cancer types when alone and/or in combination with chemotherapeutic agents as well as antibiotics that are potential new anti-cancer drugs shown to inhibit malignant cells in vivo/in vitro. The summarized data is obtained from clinicaltrials.gov, the National Institute of Health (NIH), the Drugbank, and the ReDo databank. PubMed and Google Scholar have been used to gain detailed information about the mechanism(s) of action of the potential repurposed drugs in vitro and in vivo. The keywords used for the search are "anti-cancer" and "repurposed". .

**Drug**	**Original Application / Target**	**New Application (anti-cancer) / Proposed Target/Mechanism of Action**	**Stage of Development**
**Clarithromycin**	**Antibiotic**<br>Interaction with the 50S subunit of the bacterial ribosome – inhibition of bacterial protein synthesis [1]	**Lung cancer:**<br>-As single treatment:<br>Reduces tumor cell survival [2]<br>Inhibits angiogenesis by inhibition of endothelial cell tube formation [3]<br>-In combination:<br>Enhances the effects of Vindesine sulfate and Cisplatin when given 7 days post chemotherapy → Clarithromycin: strengthens the activity of natural killer T-cells and CD8+ T cell cytotoxicity, and causes a higher number of INF-γ-/IL-4 producing T cells [2]<br>**Melanoma:**<br>-As single treatment:<br>Reduces tumor size, suppresses metastases, and increases apoptosis [3, 4]<br>-In combination:<br>Induces higher toxicity when Clarithromycin (autophagy inhibitor) is used in combination with Bortezomib (proteasome inhibitor) [5]	**Clinical Phase II:**<br>-Neoplasms (NCT02366884)<br>-Lymphoma, high dose (NCT01516606, NCT00327132, NCT01264822)<br>-Non-squamous cell lung cancer (DB01211)<br><br>**Clinical Phase II / III:**<br>-Multiple Myeloma in combination with Pomalidomide, Carfilzomib, Dexamethasone, Lenalidomide, and Thalidomide (NCT01745588, NCT01559935, and NCT02248428)<br><br>***In vivo:***<br>-Lewis lung carcinoma (RCB0558) in C57BL/6 mice [2]<br>-Melanoma B16BL6 cells in male C57BL mice [4]<br>-Adenocarcinoma cells 13762NF in a F-344 rat system [6]<br>-Colon cancer cells HCT-116 and LS174T in female nude mice [7]<br>-Lung cancer cells H157 in male ICR mice [3]

**Clarithromy-cin** *(Continued)*		**Lymphoma**:<br>-As Single treatment:<br>Induces apoptosis, DNA fragmentation, expression of TNFR1, Fas and caspase-3, -8 and -9, as well as the TNF-α system [8]<br>**Adenocarcinoma:**<br>**-**In combination:<br>Increases the therapeutic effect of Carboplatin or Cyclophosphamide [12]<br>**Spleen cells from tumor bearing rats:**<br>**-**As single treatment:<br>Lowers the expression of genes coding for TGF-β and ILK-6 [6]<br>**Myeloma cells:**<br>**-**As single treatment:<br>Inhibits autophagy by fusion of autophagosomes with lysosomes [9]<br>-In combination:<br>Enhances Thalidomide's effect [9–11]<br>**Breast cancer cells:**<br>**-**As single treatment:<br>Blocks autophagy flux [12]<br>-In Combination:<br>Enhances Bortezomib's cytotoxicity [12]<br>**Colon cancer:**<br>**-**As single treatment: Reduces tumor growth and enhances of overall-survival *in vivo* by inducting apoptosis and inhibiting autophagy [7]	***In vitro:***<br>-Lymphoma cells (300-19) [8]<br>-Spleen cells from tumor bearing rats [6]<br>-Primary myeloma cells and myeloma cell lines 12PE [9], U266, IM-9 and RPMI8226 [5]<br>-Breast cancer cell lines MDA-MB-231 and MDA-MB-468 [12, 13]<br>-Lung cancer cells H157 [3]

**Doxycycline**	**Antibiotic**<br>Interaction with the 16S subunit of the bacterial ribosomal rRNA – inhibition of the bacterial protein synthesis [14]	**Cervical cancer:**<br>**-**As single treatment:<br>Inhibits proliferation, induces apoptosis, affects oxygen consumption, glycolysis, and reduces ATP levels – targets energy metabolism [15]<br>**Colon cancer:**<br>-As single treatment:<br>Inhibits cell growth and induces G0/G1 arrest downregulates matrix metalloproteinase activity [16]<br>-In combination:<br>Enhances COX-2 inhibitor's - antiproliferative and anti-invasive effects [16]<br>**Breast cancer:**<br>-As single treatment:<br>Inhibits proliferation and viability, downregulates the expression of stem cell factors (Oct4, Sox2, Nanog and CD44) and autophagy markers (LC-3BI and LC-3BII) [17, 18]<br>-In combination:<br>Enhances radiation's effect by inhibiting DNA-PK, affecting of mitochondrial and glycolydic activity, and blocking different signaling pathways (STAT1/3, Sonic Hedgehog (Shh), Notch, WNT and TGF-β) [19]<br>**Breast, DCIS, ovarian, prostate, lung, pancreatic, melanoma, and glioblastoma:**<br>-As single treatment:<br>Disrupts mitochondrial biogenesis [20]	**Clinical Phase III:**<br>-Early-stage Lymphoma (NCT03454945)<br>-Cancer overall survival (NCT02201381)<br><br>**Clinical Phase II:**<br>-Pancreatic Cancer (NCT02775695)<br>-Non-Hodgkin Lymphomas (NCT02086591<br>-Cutaneous T-cell Lymphomas (NCT02341209)<br>-Marginal Zone Lymphoma of Ocular Adnexal (NCT01820910)<br>-Neoplasms (NCT02366884)<br><br>**Clinical Phase I:**<br>-Melanoma (NCT01590082)<br><br>***In vivo:***<br>-Hela xenograft mouse model [15]<br><br>***In vitro:***<br>-Colon cancer cells LS174T and HT29 [16]<br>-Breast cancer cells MCF10.DCIS, MCF-7, MDA-MB-468, MDA-MB-231, [17] and T47-D [19]<br>-Cervical cancer cells [15]<br>-Ovarian cancer SKOV3, Tov21G, ES2 [20]<br>-Prostate cancer cells P3C [20]<br>-Pancreas cancer cells Ma PaCa2 [36]<br>-Lung caner cells A549 [36]-Melanoma cells A379 [36]<br>-Glioma cells U-87 MG [36]

**Tigecycline**	**Antibiotic**<br>Interaction with bacterial ribosome – inhibition of protein synthesis [21]	**Breast, DCIS, ovarian, prostate, lung, pancreatic, melanoma, and glioblastoma:**<br>-As single treatment:<br>Disrupts mitochondrial biogenesis [20]<br>**Breast cancer:**<br>-As single treatment:<br>Inhibits *Rb*/*p53*-deficient tumor cell proliferation [22]<br>**Lung cancer:**<br>-As single treatment:<br>Inhibits proliferation, increases ROS, inhibits mitochondrial respiration, decrease mitochondrial membrane potential and ATP levels, and induces apoptosis [23]<br>**Neuroblastoma:**<br>-As single treatment:<br>Inhibits the Akt pathway and induces G1 arrest [24]<br>**Myeloma:**<br>-As single treatment:<br>Overexpresses p21, induces G0/G1 arrest, inhibits cell proliferation [25]<br>-In combination with the afilomycin A1 (autophagy inhibitor)<br>Impairs colony formation, induces G0/G1 arrest, downregulates p21, CDK2 and cyclin D1, induces autophagy [26]<br>**Glioma:**<br>-As single treatment:<br>Increases miR-199b-5p → HES1 downregulation → affects Akt pathway → inhibits proliferation, induces cell cycle arrest, elevates p21 expression [27]	**Clinical trial Phase I:**<br>-Acute myeloid leukemia (NCT01332786)<br><br>***In vivo:***<br>-Triple-negative breast cancer cells in mice [22]<br>-Lung cancer cells A549 in SCID mice [23]<br>-A355 and MV3 cells in female nude mice [25]<br>-SDIC mice with BE2C and SK-N-AS cells [24]<br>-Glioma cells U87 in nude mice [27]<br>-Myeloma cells RPMI-8226 in NOD/SCID mice [26]

**Tigecycline** *(Continued)*		**Leukemia:**<br>-As single treatment:<br>Inhibits mitochondrial biogenesis and function → induction of apoptosis, downregulation of the PI3K-AKT-mTOR pathway →induction of autophagy<br>-In combination with autophagy inhibitors like 3-Methyladenine or Chloroquine<br>Enhances the anti-cancer effect [28, 29]<br>**Cervical cancer:**<br>-As single treatment:<br>Inhibits the Wnt/β-catenin signaling pathway and expression → inhibition of proliferation, induction of apoptosis, inhibits colony formation<br>-In combination:<br>Enhances Paclitaxel' effect [30]	***In vitro:***<br>-Ovarian cancer SKOV3, Tov21G, ES2 [20]<br>-Prostate cancer cells P3C [20]<br>-Pancreas cancer cells Ma PaCa2 [36]<br>-Lung caner cells A549 [36], PC9, H157, and H1975 [23]<br>-Melanoma cells A379 [36], A375 and MV3 [25]<br>-Glioma cells U-87 MG [36] U118, and U251 [27]<br>-Breast cancer cells MCF10.DCIS, MCF-7, T47D, and MDA-MB-231 [20]TMCRP1, TMCRP2, TMCRP3, RB400, TC53-120, TC53-127, MDA- MB-231, and Hs578T [22],<br>-Neuroblastoma: BE2C and SK-N-AS [24]<br>-Chronic myeloid leukemia cells [28]<br>- Melanoma: RPMI-8226, NCI-H929 and U266 [26]<br>-Cervical cancer: Hela cells [30]
**Cefepime**	**Antibiotic**<br>Inhibition of bacterial cell wall synthesis [31]	**Breast cancer:**<br>**-**As single treatment but as a Manganese-Cefepime complex:<br>Inhibits cell proliferation and proteasome activity [32]<br>-In combination:<br>Enhances radiation' effect (radiosensitizer) [33]	***In vivo:***<br>-Murine melanoma B16.SIY cells in C57BL/6 female mice [33]<br><br>***In vitro:***<br>-Breast cancer cells MDA-MB-231[32]<br>-Melanoma B16.SIY, breast cancer MDA-MB-435 and head cancer [33]

**Ciprofloxacin**	**Antibiotic**<br>Inhibits bacterial gyrase – inhibition of the DNA replication [34–36]	**Transitional cancer:**<br>**-**As single treatment:<br>Inhibits cell growth [37]<br>**Glioblastoma:**<br>**-**As single treatment:<br>Inhibits cell proliferation, induces cell cycle arrest, downregulates glutathione level, induces mitochondrial dysfunction and apoptosis via caspase-3/7 activation [38]<br>**Breast cancer:**<br>**-**As single treatment:<br>Downregulates cell viability, alters the redox signaling pathway, disrupts mitochondrial membrane, induces cell cycle arrest/DNA fragmentation/p53-dependent apoptosis [39]<br>**Melanoma:**<br>**-**As single treatment:<br>Decreases cell viability, induces DNA fragmentation and cell cycle arrest, disrupts mitochondrial membrane, and induces apoptosis [40]<br>**Colon cancer:**<br>**-**As single treatment:<br>Inhibits proliferation, induces cell cycle arrest, and upregulates TGF-β1 [41]<br><br>More extensive summary is presented in the review of Yadav and Talwar [42]	***In vitro:***<br>-Transitional cancer cells MBT-2 [37]<br>-Glioblastoma cells U87MG [38]<br>-Breast cancer cells MDA MB-231 [39]<br>-Melanoma cells Colo 829 [40]<br>-Colon cancer cells HT-29 [41]

**Nitroxoline**	**Antibiotic**<br>Chelates divalent cations → interrupts RNA synthesis [43–45]	**Bladder and breast cancer:**<br>**-**As single treatment:<br>Inhibits MetAP2 activity, induces senescence, inhibits endothelial tube formation, reduces micro-vessel density → decrease angiogenesis [46]<br>**Lymphoma, leukemia, pancreatic cancer:**<br>**-**As single treatment:<br>Induces ROS, an effect that is enhanced in presence of copper [47]<br>**Prostate cancer:**<br>**-**As single treatment:<br>Inhibits AMPK- mTOR-p70S6K signaling pathway and cyclin D1-Rb-Cdc25A axis → leading to cell cycle arrest and apoptosis [48]<br>**Glioma:**<br>**-**As single treatment:<br>Inhibits cell growth, induces cell cycle arrest and apoptosis via caspase 3 and poly (ADP-ribose) polymerase cleavage [49]<br>**Multi cancer types investigation:**<br>**-**As single treatment:<br>Inhibits cell proliferation [50]	***In vivo:***<br>-Breast cancer cells HCC1954 in female athymic nude mice [46]<br>-Bladder cancer cells KU7 in female athymic nude mice [46]<br>-Bladder cancer cells 5637 and T24 in nude mice [50]<br>-Renal tumor cells KCC853 in mice [50]<br>-Mouse glioma model [49]<br><br>***In vitro:***<br>-Bladder cancer cells KU7 [46]<br>-Breast cancer cells HCC1954 [46]<br>-Lymphoma Raji [47]<br>-Leukemia HL-60 [47]<br>-Pancreatic cancer Pan-1 [47]<br>-Broad spectrum cancer analysis, among them HUVEC, HepG2, A549, LoVo, MCF7, T24, 5637 and J82 cells [50]<br>-Prostate cancer PC-3, DU-145 and LNCaP cells [48]<br>-Glioma cells U87 and U251[49]
**Fleroxacin**	**Antibiotic**<br>DNA damage – inhibition of topoisomerase [34, 51]	**Transitional cancer:**<br>-As single treatment:<br>Inhibition of cell proliferation [37]	***In vitro:***<br>-Transitional carcinoma cells MBT-2 and T24 [37]
**Cephalexin**<br>**Cefaclor**<br>**Cephradine**<br>**Cefixime**	**Antibiotics**<br>Inhibition of bacterial cell wall synthesis [71–75]	**Melanoma, breast cancer:**<br>-In combination<br>Enhances radiation' effect (radiosensitizer) [33]	***In vivo:***<br>-Murine melanoma B16.SIY cells in C57BL/6 female mice [33]<br>***In vitro:***<br>-Breast cancer [33] MDA-MB-435 (Cephalexin)<br>-Melanoma B16.SIY, breast cancer MDA-MB-435 and head cancer [33] (Cefaclor)<br>-Breast cancer MDA-MB-435 and head cancer [33] (Cephradine/Cefixime)

**Minocycline**	**Antibiotic** (also known as an anti-inflamma-tory drug [52])<br><br>Inhibits protein synthesis [53]	**Ovarian cancer:**<br>**-**As single treatment:<br>Inhibits cell and colony formation, induces cell-cycle arrest with a downregulation of cyclins A, B, and E and suppression of DNA synthesis, induces caspase-3 and PARP-1 cleavage, inhibits *in vivo* angiogenesis and tumor growth [54]<br>Suppresses IL-6 expression, modulates the IL-6 receptor System, and suppresses TGF-β1-TAK1-IκB signaling [55, 56]<br>**Breast cancer:**<br>-In combination with Celecoxib:<br>Inhibits cell growth, decreases micro-vessel density, lowers the expression of vascular endothelial growth factor (VEGF) and matrix metalloproteinase (MMP)-9 [57]<br>**Glioma:**<br>**-**As single treatment:<br>Inhibits cell growth, inhibits membrane type 1 (MT1-MMP) expression in malignant cells [58]<br>Induces autophagy mediated cell death [59]	***In vivo:***<br>-Ovarian cancer OVCAR-3 cells in female nude mice [55, 56, 60]<br>-Breast cancer cells MDA-MB-435S in nude mice [57]<br>-Glioma cells GL261 in wild type C57BL/6 mice [58]<br><br>***In vitro:***<br>-Ovarian cancer cells OVCAR-3, CAOV-3, SKOV-3 and A2780 [54–56, 61]<br>-Glioma cells U87 and U251 [59]
**Levofloxacin**	**Antibiotic**<br>DNA damage – inhibition of topoisomerase [34]	**Breast cancer:**<br>**-**As single treatment:<br>Inhibits mitochondrial biogenesis, and activates PI3K/Akt/mTOR and MAPK/ERK pathways [62]<br>-In combination<br>Synergizes 5-Fluorouracil's effect [62]<br>**Lung cancer:**<br>**-**As single treatment:<br>Inhibits cell proliferation and mitochondrial activity, increases high levels of ROS → oxidative damage →induces apoptosis [63]<br>A more in depth summary is presented in the review by Yadav and Talwar [42]	***In vivo:***<br>-Breast cancer cells MCF-7 or MDA-MB-231 in SCID mice [62]<br>-Lung cancer cells H460 in SCID mice [63]<br><br>***In vitro:***<br>-Breast cancer cells MCF-7, MDA-MB-231, MDA-MB-468 and SkBr-3 [62]<br>-Lung cancer cells A549, H3255, NCL-69, H460 [63]

**Enoxacin**	**Antibiotic**<br>DNA damage – inhibition of topoisomerase [34, 67]	**Prostate cancer:**<br>**-**As single treatment:<br>downregulates cell viability, induces cell cycle arrest and apoptosis, inhibits invasion, restores global miRNA expression (malfunctional in prostate cancer) [68]<br>**Cervical cancer:**<br>**-**As single treatment:<br>Inhibits cell growth, induces apoptosis via caspase-9, -3 and -7 activation, upregulates p21, SNF, PUMA NOXA, and BAF mRNA expression [69]<br>-In combination:<br>Synergizes Epigallocatechin Gallate's effect [69]<br>**Breast cancer:**<br>**-**As single treatment:<br>Inhibits cell proliferation, alters cellular morphology, and induces cell cycle arrest [70]<br>More data is summarized in the review by Yadav and Talwar [42]	***In vitro:***<br>-Prostate cancer cells DU145, LNCaP, VCaP and PC-3 [68]<br>-Cervical cancer cells HeLa, C33A, and WI-38 [69]<br>-Breast cancer cells MCF-7 [70]
**Moxifloxacin**	**Antibiotic**<br>DNA damage – inhibition of topoisomerase [34, 64]	**Glioblastoma:**<br>**-**As single treatment:<br>Inhibits cell proliferation, induces cell cycle arrest, downregulates glutathione level, and induces mitochondrial dysfunction and caspase dependent apoptosis [38]<br>**Pancreatic cancer:**<br>**-**As single treatment:<br>Inhibits cell proliferation, induces cell cycle arrest, downregulates p27, p21, CDK2, cyclin-A and cyclin-E expression, activates caspase-8, 9, 3 via Bid and ERK 1/2 [65]<br>**Breast cancer:**<br>**-**As single treatment:<br>Inhibits cell proliferation, induces apoptosis by a Cu-moxifloxacin complex (and nitrogen adducts) [66]	***In vitro:***<br>-Glioblastoma cells U87MG [38]<br>-Pancreatic cancer cells MIA PaCa-2 and Panc-1 [65]<br>-Breast cancer cells MCF-7, T47D, MDA-MB-231, and BT-20 [66]

References

[1] Champney, W.S.; Burdine, R. Azithromycin and clarithromycin inhibition of 50S ribosomal subunit formation in Staphylococcus aureus cells. *Current microbiology*, **1998**, *36,* 119–123.

[2] Hamada, K.; Mikasa, K.; Yunou, Y.; Kurioka, T.; Majima, T.; Narita, N.; Kita, E. Adjuvant effect of clarithromycin on chemotherapy for murine lung cancer. *Chemotherapy*, **2000**, *46,* 49–61.

[3] Yatsunami, J.; Turuta, N.; Wakamatsu, K.; Hara, N.; Hayashi, S. Clarithromycin is a potent inhibitor of tumor-induced angiogenesis. *Research in experimental medicine. Zeitschrift fur die gesamte experimentelle Medizin einschliesslich experimenteller Chirurgie*, **1997**, *197,* 189–197.

[4] Yatsunami, J.; Fukuno, Y.; Nagata, M.; Tominaga, M.; Aoki, S.; Tsuruta, N.; Kawashima, M.; Taniguchi, S.; Hayashi, S. Antiangiogenic and antitumor effects of 14-membered ring macrolides on mouse B16 melanoma cells. *Clinical & experimental metastasis*, **1999**, *17,* 361–367.

[5] Moriya, S.; Che, X.-F.; Komatsu, S.; Abe, A.; Kawaguchi, T.; Gotoh, A.; Inazu, M.; Tomoda, A.; Miyazawa, K. Macrolide antibiotics block autophagy flux and sensitize to bortezomib via endoplasmic reticulum stress-mediated CHOP induction in myeloma cells. *International journal of oncology*, **2013**, *42,* 1541–1550.

[6] Sassa, K.; Mizushima, Y.; Fujishita, T.; Oosaki, R.; Kobayashi, M. Therapeutic effect of clarithromycin on a transplanted tumor in rats. *Antimicrobial agents and chemotherapy*, **1999**, *43,* 67–72.

[7] Petroni, G.; Stefanini, M.; Pillozzi, S.; Crociani, O.; Becchetti, A.; Arcangeli, A. Data describing the effects of the Macrolide Antibiotic Clarithromycin on preclinical mouse models of Colorectal Cancer. *Data in brief*, **2019**, *26,* 104406.

[8] Ohara, T.; Morishita, T.; Suzuki, H.; Masaoka, T.; Ishii, H.; Hibi, T. Antibiotics directly induce apoptosis in B cell lymphoma cells derived from BALB/c mice. *Anticancer research*, **2004**, *24,* 3723–3730.

[9] Hata. Clarithromycin attenuates autophagy in myeloma cells. *Int J Oncol*, **2010**, *37*.

[10] Coleman, M.; Leonard, J.; Lyons, L.; Pekle, K.; Nahum, K.; Pearse, R.; Niesvizky, R.; Michaeli, J. BLT-D (clarithromycin Biaxin, low-dose thalidomide, and dexamethasone) for the treatment of myeloma and Waldenström's macroglobulinemia. *Leukemia & lymphoma*, **2002**, *43,* 1777–1782.

[11] Schafranek, L.; Leclercq, T.M.; White, D.L.; Hughes, T.P. Clarithromycin enhances dasatinib-induced cell death in chronic myeloid leukemia cells, by inhibition of late stage autophagy. *Leukemia & lymphoma*, **2013**, *54,* 198–201.

[12] Komatsu, S.; Miyazawa, K.; Moriya, S.; Takase, A.; Naito, M.; Inazu, M.; Kohno, N.; Itoh, M.; Tomoda, A. Clarithromycin enhances bortezomib-induced cytotoxicity via endoplasmic reticulum stress-mediated CHOP (GADD153) induction and autophagy in breast cancer cells. *Int J Oncol*, **2012**, *40,* 1029–1039.

[13] Komatsu, S.; Moriya, S.; Che, X.-F.; Yokoyama, T.; Kohno, N.; Miyazawa, K. Combined treatment with SAHA, bortezomib, and clarithromycin for concomitant targeting of aggresome formation and intracellular proteolytic pathways enhances ER stress-mediated cell death in breast cancer cells. *Biochemical and biophysical research communications*, **2013**, *437,* 41–47.

[14] Chukwudi, C.U. rRNA Binding Sites and the Molecular Mechanism of Action of the Tetracyclines. *Antimicrobial agents and chemotherapy*, **2016**, *60,* 4433–4441.

[15] Zhao, Y.; Wang, X.; Li, L.; Li, C. Doxycycline inhibits proliferation and induces apoptosis of both human papillomavirus positive and negative cervical cancer cell lines. *Canadian journal of physiology and pharmacology*, **2016**, *94,* 526–533.

[16] Onoda, T.; Ono, T.; Dhar, D.K.; Yamanoi, A.; Fujii, T.; Nagasue, N. Doxycycline inhibits cell proliferation and invasive potential: combination therapy with cyclooxygenase-2 inhibitor in human colorectal cancer cells. *The Journal of laboratory and clinical medicine*, **2004**, *143,* 207–216.

[17] Le Zhang; Xu, L.; Zhang, F.; Vlashi, E. Doxycycline inhibits the cancer stem cell phenotype and epithelial-to-mesenchymal transition in breast cancer. *Cell cycle (Georgetown, Tex.)*, **2017**, *16,* 737–745.

[18] Scatena, C.; Roncella, M.; Di Paolo, A.; Aretini, P.; Menicagli, M.; Fanelli, G.; Marini, C.; Mazzanti, C.M.; Ghilli, M.; Sotgia, F.; Lisanti, M.P.; Naccarato, A.G. Doxycycline, an Inhibitor of Mitochondrial Biogenesis, Effectively Reduces Cancer Stem Cells (CSCs) in Early Breast Cancer Patients: A Clinical Pilot Study. *Frontiers in oncology*, **2018**, *8,* 452.

[19] Lamb, R.; Fiorillo, M.; Chadwick, A.; Ozsvari, B.; Reeves, K.J.; Smith, D.L.; Clarke, R.B.; Howell, S.J.; Cappello, A.R.; Martinez-Outschoorn, U.E.; Peiris-Pagès, M.; Sotgia, F.; Lisanti, M.P. Doxycycline down-regulates DNA-PK and radiosensitizes tumor initiating cells: Implications for more effective radiation therapy. *Oncotarget*, **2015**, *6,* 14005–14025.

[20] Lamb, R.; Ozsvari, B.; Lisanti, C.L.; Tanowitz, H.B.; Howell, A.; Martinez-Outschoorn, U.E.; Sotgia, F.; Lisanti, M.P. Antibiotics that target mitochondria effectively eradicate cancer stem cells, across multiple tumor types: treating cancer like an infectious disease. *Oncotarget*, **2015**, *6,* 4569–4584.

[21] Beabout, K.; Hammerstrom, T.G.; Perez, A.M.; Magalhães, B.F.; Prater, A.G.; Clements, T.P.; Arias, C.A.; Saxer, G.; Shamoo, Y. The ribosomal S10 protein is a general target for decreased tigecycline susceptibility. *Antimicrobial agents and chemotherapy*, **2015**, *59*, 5561–5566.

[22] Jones, R.A.; Robinson, T.J.; Liu, J.C.; Shrestha, M.; Voisin, V.; Ju, Y.; Chung, P.E.D.; Pellecchia, G.; Fell, V.L.; Bae, S.; Muthuswamy, L.; Datti, A.; Egan, S.E.; Jiang, Z.; Leone, G.; Bader, G.D.; Schimmer, A.; Zacksenhaus, E. RB1 deficiency in triple-negative breast cancer induces mitochondrial protein translation. *The Journal of clinical investigation*, **2016**, *126*, 3739–3757.

[23] Jia, X.; Gu, Z.; Chen, W.; Jiao, J. Tigecycline targets nonsmall cell lung cancer through inhibition of mitochondrial function. *Fundamental & clinical pharmacology*, **2016**, *30*, 297–306.

[24] Zhong, X.; Zhao, E.; Tang, C.; Zhang, W.; Tan, J.; Dong, Z.; Ding, H.-F.; Cui, H. Antibiotic drug tigecycline reduces neuroblastoma cells proliferation by inhibiting Akt activation in vitro and in vivo. *Tumour biology : the journal of the International Society for Oncodevelopmental Biology and Medicine*, **2016**, *37*, 7615–7623.

[25] Hu, H.; Dong, Z.; Tan, P.; Zhang, Y.; Liu, L.; Yang, L.; Liu, Y.; Cui, H. Antibiotic drug tigecycline inhibits melanoma progression and metastasis in a p21CIP1/Waf1-dependent manner. *Oncotarget*, **2016**, *7*, 3171–3185.

[26] Ma, R.; Zhang, Y.; Wang, W.; Wu, J.; Yang, Q.; Xu, W.; Jiang, S.; Han, Y.; Yu, K.; Zhang, S. Inhibition of autophagy enhances the antitumour activity of tigecycline in multiple myeloma. *Journal of cellular and molecular medicine*, **2018**, *22*, 5955–5963.

[27] Yang, R.; Yi, L.; Dong, Z.; Ouyang, Q.; Zhou, J.; Pang, Y.; Wu, Y.; Xu, L.; Cui, H. Tigecycline Inhibits Glioma Growth by Regulating miRNA-199b-5p-HES1-AKT Pathway. *Molecular cancer therapeutics*, **2016**, *15*, 421–429.

[28] Lu, Z.; Xu, N.; He, B.; Pan, C.; Lan, Y.; Zhou, H.; Liu, X. Inhibition of autophagy enhances the selective anti-cancer activity of tigecycline to overcome drug resistance in the treatment of chronic myeloid leukemia. *Journal of experimental & clinical cancer research : CR*, **2017**, *36*, 43.

[29] Skrtić, M.; Sriskanthadevan, S.; Jhas, B.; Gebbia, M.; Wang, X.; Wang, Z.; Hurren, R.; Jitkova, Y.; Gronda, M.; Maclean, N.; Lai, C.K.; Eberhard, Y.; Bartoszko, J.; Spagnuolo, P.; Rutledge, A.C.; Datti, A.; Ketela, T.; Moffat, J.; Robinson, B.H.; Cameron, J.H.; Wrana, J.; Eaves, C.J.; Minden, M.D.; Wang, J.C.Y.; Dick, J.E.; Humphries, K.; Nislow, C.; Giaever, G.; Schimmer, A.D. Inhibition of mitochondrial translation as a therapeutic strategy for human acute myeloid leukemia. *Cancer cell*, **2011**, *20*, 674–688.

[30] Li, H.; Jiao, S.; Li, X.; Banu, H.; Hamal, S.; Wang, X. Therapeutic effects of antibiotic drug tigecycline against cervical squamous cell carcinoma by inhibiting Wnt/β-catenin signaling. *Biochemical and biophysical research communications*, **2015**, *467*, 14–20.

[31] Chapman, T.M.; Perry, C.M. Cefepime: a review of its use in the management of hospitalized patients with pneumonia. *American journal of respiratory medicine : drugs, devices, and other interventions*, **2003**, *2*, 75–107.

[32] Zhang, Z.; Bi, C.; Fan, Y.; Wang, H.; Bao, Y. Cefepime, a fourth-generation cephalosporin, in complex with manganese, inhibits proteasome activity and induces the apoptosis of human breast cancer cells. *International journal of molecular medicine*, **2015**, *36*, 1143–1150.

[33] Labay, E.; Mauceri, H.J.; Efimova, E.V.; Flor, A.C.; Sutton, H.G.; Kron, S.J.; Weichselbaum, R.R. Repurposing cephalosporin antibiotics as pro-senescent radiosensitizers. *Oncotarget*, **2016**, *7*, 33919–33933.

[34] Drlica, K. Mechanism of fluoroquinolone action. *Current Opinion in Microbiology*, **1999**, *2*, 504–508.

[35] Shen, L.L. Molecular mechanisms of DNA gyrase inhibition by quinolone antibacterials. *Advances in pharmacology (San Diego, Calif.)*, **1994**, *29A*, 285–304.

[36] Hooper, D.C.; Wolfson, J.S.; Ng, E.Y.; Swartz, M.N. Mechanisms of action of and resistance to ciprofloxacin. *The American Journal of Medicine*, **1987**, *82*, 12–20.

[37] Ebisuno, S.; Inagaki, T.; Kohjimoto, Y.; Ohkawa, T. The cytotoxic effects of fleroxacin and ciprofloxacin on transitional cell carcinoma in Vitro. *Cancer*, **1997**, *80*, 2263–2267.

[38] Beberok, A.; Rzepka, Z.; Respondek, M.; Rok, J.; Sierotowicz, D.; Wrześniok, D. GSH depletion, mitochondrial membrane breakdown, caspase-3/7 activation and DNA fragmentation in U87MG glioblastoma cells: New insight into the mechanism of cytotoxicity induced by fluoroquinolones. *European journal of pharmacology*, **2018**, *835*, 94–107.

[39] Beberok, A.; Wrześniok, D.; Rok, J.; Rzepka, Z.; Respondek, M.; Buszman, E. Ciprofloxacin triggers the apoptosis of human triple-negative breast cancer MDA-MB-231 cells via the p53/Bax/Bcl-2 signaling pathway. *International journal of oncology*, **2018**, *52*, 1727–1737.

[40] Beberok, A.; Wrześniok, D.; Minecka, A.; Rok, J.; Delijewski, M.; Rzepka, Z.; Respondek, M.; Buszman, E. Ciprofloxacin-mediated induction of S-phase cell cycle arrest and apoptosis in COLO829 melanoma cells. *Pharmacological reports : PR*, **2018**, *70*, 6–13.

[41] Bourikas, L.A.; Kolios, G.; Valatas, V.; Notas, G.; Drygiannakis, I.; Pelagiadis, I.; Manousou, P.; Klironomos, S.; Mouzas, I.A.; Kouroumalis, E. Ciprofloxacin decreases survival in HT-29 cells via the induction of TGF-beta1 secretion and enhances the anti-proliferative effect of 5-fluorouracil. *British journal of pharmacology*, **2009**, *157*, 362–370.

[42] Yadav, V.; Talwar, P. Repositioning of fluoroquinolones from antibiotic to anti-cancer agents: An underestimated truth. *Biomedicine & pharmacotherapy = Biomedecine & pharmacotherapie*, **2019**, *111*, 934–946.

[43] Pelletier, C.; Prognon, P.; Bourlioux, P. Roles of divalent cations and pH in mechanism of action of nitroxoline against Escherichia coli strains. *Antimicrobial agents and chemotherapy*, **1995**, *39*, 707–713.

[44] Wijma, R.A.; Huttner, A.; Koch, B.C.P.; Mouton, J.W.; Muller, A.E. Review of the pharmacokinetic properties of nitrofurantoin and nitroxoline. *The Journal of antimicrobial chemotherapy*, **2018**, *73*, 2916–2926.

[45] Kresken, M.; Körber-Irrgang, B. In vitro activity of nitroxoline against Escherichia coli urine isolates from outpatient departments in Germany. *Antimicrobial agents and chemotherapy*, **2014**, *58*, 7019–7020.

[46] Shim, J.S.; Matsui, Y.; Bhat, S.; Nacev, B.A.; Xu, J.; Bhang, H.C.; Dhara, S.; Han, K.C.; Chong, C.R.; Pomper, M.G.; So, A.; Liu, J.O. Effect of nitroxoline on angiogenesis and growth of human bladder cancer. *Journal of the National Cancer Institute*, **2010**, *102*, 1855–1873.

[47] Jiang, H.; Taggart, J.E.; Zhang, X.; Benbrook, D.M.; Lind, S.E.; Ding, W.-Q. Nitroxoline (8-hydroxy-5-nitroquinoline) is more a potent anti-cancer agent than clioquinol (5-chloro-7-iodo-8-quinoline). *Cancer letters*, **2011**, *312*, 11–17.

[48] Chang, W.-L.; Hsu, L.-C.; Leu, W.-J.; Chen, C.-S.; Guh, J.-H. Repurposing of nitroxoline as a potential anticancer agent against human prostate cancer: a crucial role on AMPK/mTOR signaling pathway and the interplay with Chk2 activation. *Oncotarget*, **2015**, *6*, 39806–39820.

[49] Lazovic, J.; Guo, L.; Nakashima, J.; Mirsadraei, L.; Yong, W.; Kim, H.J.; Ellingson, B.; Wu, H.; Pope, W.B. Nitroxoline induces apoptosis and slows glioma growth in vivo. *Neuro-oncology*, **2015**, *17*, 53–62.

[50] Zhang, Q.I.; Wang, S.; Yang, D.; Pan, K.; Li, L.; Yuan, S. Preclinical pharmacodynamic evaluation of antibiotic nitroxoline for anticancer drug repurposing. *Oncology letters*, **2016**, *11*, 3265–3272.

[51] Balfour, J.A.; Todd, P.A.; Peters, D.H. Fleroxacin. A review of its pharmacology and therapeutic efficacy in various infections. *Drugs*, **1995**, *49*, 794–850.

[52] Garrido-Mesa, N.; Zarzuelo, A.; Gálvez, J. Minocycline: far beyond an antibiotic. *British journal of pharmacology*, **2013**, *169*, 337–352.

[53] Gelbe Liste Phramindex. Minocyclin, **March 22, 2021**.

[54] Pourgholami, M.H.; Mekkawy, A.H.; Badar, S.; Morris, D.L. Minocycline inhibits growth of epithelial ovarian cancer. *Gynecologic oncology*, **2012**, *125*, 433–440.

[55] Ataie-Kachoie, P.; Badar, S.; Morris, D.L.; Pourgholami, M.H. Minocycline targets the NF-κB Nexus through suppression of TGF-β1-TAK1-IκB signaling in ovarian cancer. *Molecular cancer research : MCR*, **2013**, *11*, 1279–1291.

[56] Ataie-Kachoie, P.; Morris, D.L.; Pourgholami, M.H. Minocycline suppresses interleukine-6, its receptor system and signaling pathways and impairs migration, invasion and adhesion capacity of ovarian cancer cells: in vitro and in vivo studies. *PloS one*, **2013**, *8*, e60817.

[57] Niu, G.; Liao, Z.; Cai, L.; Wei, R.; Sun, L. The combined effects of celecoxib and minocycline hydrochloride on inhibiting the osseous metastasis of breast cancer in nude mice. *Cancer biotherapy & radiopharmaceuticals*, **2008**, *23*, 469–476.

[58] Markovic, D.S.; Vinnakota, K.; van Rooijen, N.; Kiwit, J.; Synowitz, M.; Glass, R.; Kettenmann, H. Minocycline reduces glioma expansion and invasion by attenuating microglial MT1-MMP expression. *Brain, behavior, and immunity*, **2011**, *25*, 624–628.

[59] Liu, W.-T.; Lin, C.-H.; Hsiao, M.; Gean, P.-W. Minocycline inhibits the growth of glioma by inducing autophagy. *Autophagy*, **2011**, *7*, 166–175.

[60] Pourgholami, M.H.; Wangoo, K.T.; Morris, D.L. Albendazole-cyclodextrin complex: enhanced cytotoxicity in ovarian cancer cells. *Anticancer research*, **2008**, *28*, 2775–2779.

[61] Pourgholami, M.H.; Szwajcer, M.; Chin, M.; Liauw, W.; Seef, J.; Galettis, P.; Morris, D.L.; Links, M. Phase I clinical trial to determine maximum tolerated dose of oral albendazole in patients with advanced cancer. *Cancer chemotherapy and pharmacology*, **2010**, *65*, 597–605.

[62] Yu, M.; Li, R.; Zhang, J. Repositioning of antibiotic levofloxacin as a mitochondrial biogenesis inhibitor to target breast cancer. *Biochemical and biophysical research communications*, **2016**, *471*, 639–645.
[63] Song, M.; Wu, H.; Wu, S.; Ge, T.; Wang, G.; Zhou, Y.; Sheng, S.; Jiang, J. Antibiotic drug levofloxacin inhibits proliferation and induces apoptosis of lung cancer cells through inducing mitochondrial dysfunction and oxidative damage. *Biomedicine & pharmacotherapy = Biomedecine & pharmacotherapie*, **2016**, *84*, 1137–1143.
[64] Keating, G.M.; Scott, L.J. Moxifloxacin: a review of its use in the management of bacterial infections. *Drugs*, **2004**, *64*, 2347–2377.
[65] Yadav, V.; Varshney, P.; Sultana, S.; Yadav, J.; Saini, N. Moxifloxacin and ciprofloxacin induces S-phase arrest and augments apoptotic effects of cisplatin in human pancreatic cancer cells via ERK activation. *BMC cancer*, **2015**, *15*, 581.
[66] Patitungkho, S.; Adsule, S.; Dandawate, P.; Padhye, S.; Ahmad, A.; Sarkar, F.H. Synthesis, characterization and anti-tumor activity of moxifloxacin-copper complexes against breast cancer cell lines. *Bioorganic & medicinal chemistry letters*, **2011**, *21*, 1802–1806.
[67] Henwood, J.M.; Monk, J.P. Enoxacin. A review of its antibacterial activity, pharmacokinetic properties and therapeutic use. *Drugs*, **1988**, *36*, 32–66.
[68] Sousa, E.; Graça, I.; Baptista, T.; Vieira, F.Q.; Palmeira, C.; Henrique, R.; Jerónimo, C. Enoxacin inhibits growth of prostate cancer cells and effectively restores microRNA processing. *Epigenetics*, **2013**, *8*, 548–558.
[69] McDonnell, A.M.; Pyles, H.M.; Diaz-Cruz, E.S.; Barton, C.E. Enoxacin and Epigallocatechin Gallate (EGCG) Act Synergistically to Inhibit the Growth of Cervical Cancer Cells in Culture. *Molecules (Basel, Switzerland)*, **2019**, *24*.
[70] Mukherjee, P.; Mandal, E.R.; Das, S.K. Evaluation of Antiproliferative Activity of Enoxacin on a Human Breast Cancer Cell Line. *International Journal of Human Genetics*, **2005**, *5*, 57–63.
[71] Sader, H.S.; Jacobs, M.R.; Fritsche, T.R. Review of the spectrum and potency of orally administered cephalosporins and amoxicillin/clavulanate. *Diagnostic microbiology and infectious disease*, **2007**, *57*, 5S-12S.
[72] Tanrisever, B.; Santella, P.J. Cefadroxil. A review of its antibacterial, pharmacokinetic and therapeutic properties in comparison with cephalexin and cephradine. *Drugs*, **1986**, *32 Suppl 3*, 1–16.
[73] Chambers, H.F.; Miick, C. Characterization of penicillin-binding protein 2 of Staphylococcus aureus: deacylation reaction and identification of two penicillin-binding peptides. *Antimicrobial agents and chemotherapy*, **1992**, *36*, 656–661.
[74] Klastersky, J.; Daneau, D.; Weerts, D. Cephradine. Antibacterial activity and clinical effectiveness. *Chemotherapy*, **1973**, *18*, 191–204.
[75] Markham, A.; Brogden, R.N. Cefixime. A review of its therapeutic efficacy in lower respiratory tract infections. *Drugs*, **1995**, *49*, 1007–1022.
