# Supplementary material for: Repurposing of Antimicrobial Agents for Cancer Therapy: What Do We Know?
Source: Cancers (Basel). 2021 Jun 26;13(13):3193. doi: 10.3390/cancers13133193 (PMC8269327; doi:10.3390/cancers13133193)
Supplement: Supplementary file 1 [file cancers-13-03193-s001.zip › Supplemental Data Table S2.pdf]

Table S2: List of antiviral agents that are currently being investigated in clinical trials for their effectiveness against different cancer types when alone and/or in combination with chemotherapeutic agents, as well as antivirals that are potential new anti-cancer drugs shown to inhibit malignant cells *in vivo*/*in vitro*. The summarized data is obtained from clinicaltrials.gov, the National Institute of Health (NIH), the Drugbank, and the ReDo databank. PubMed and Google Scholar have been used to gain detailed information about the mechanism(s) of action of the potential repurposed drugs *in vitro* and *in vivo*. The keywords used for the search are "anti-cancer" and "repurposed".

Drug	Original application / target	New application (anti-cancer) / Proposed Target/Mechanism of action	Stage of development
**Amantadine**	**Antiviral**<br>Blocks the viral M2 ion channel → prevents virus entry to host cells [1–3]	**Cancer detection:**<br>-As single agent:<br>Acts as diagnostic biomarker that confirms the presence of cancer cells through its metabolism by spermidine/spermine N acetyltransferase (SSAT), highly increased in cancer cells	**Clinical Trial (Recruiting, Phase II):**<br>Detection of cancer (NCT02277938, NCT00755898)
**Cidofovir**	**Antiviral**<br>Inhibits viral DNA polymerase [4]	**Glioblastoma:**<br>-As single treatment:<br>Incorporates into the DNA of the tumor cells → promotes double strand breaks → induces apoptosis [5]<br>-In combination as a Radiosensitizer [5]<br><br>**Cervical cancer (Human Papillomavirus (HPV) positive):**<br>-As single treatment:<br>Causes DNA damage, activates Rho GTPase pathways and LXR/RXR pathways, enhances levels of p53 and p-pRb, and induces cell cycle arrest and cell death [6, 7]<br><br>-In combination with chemo-radiotherapy:<br>Downregulates virus induced-E6 and E7 levels, enhances p53 and p-pRb levels, and downregulates p16 levels [8]	**Clinical Trial Phase II:**<br>-Anal Cancer of HIV infected patients (NCT00550589, NCT01946009)<br>-Squamous Intraepithelial Lesions (NCT02976987)<br><br>**Clinical Trial Phase I:**<br>-Radiosensitizer in cervical cancer (NCT00811408, NCT02515877)<br><br>***In vivo:***<br>-Glioblastoma cells U87MG and SF7796 in athymic mice [5]<br>-Cervical cancer cells HeLa and SiHa in nude mice [7]

**Cidofovir** **(***continued)*		**Breast, colon, liver, hepatocyte, prostate, and cervical carcinomas:**<br>-As single treatment:<br>Reduces cell viability (HPV positive and negative cells), increases PARP, p85, p53, cytochrome c and caspase-3 levels, increases Bax/Bcl-2-ratio, and causes DNA fragmentation [9]	***In vitro:***<br>-Glioblastoma cells: U87MG and primary SF7796 [5]<br>-Cervical cancer cells: HPV16+ (SiHa) and HPV18+ (HeLa) [6, 9]<br>-Colon cancer cells: Caco-2 [9]<br>-Hepatocyte carcinoma cells: Hep-G2 [9]<br>-Prostate cancer cells: PC-3 [9]<br>-Breast cancer cells: MDA-MB-231 [9]<br>-Lung cancer cells: NCI-H1975 [9]
**Efavirenz**	**Antiviral**<br>Downregulates the activity of the viral non-nucleoside reverse transcriptase [10, 11]	**Colorectal and pancreatic carcinoma, glioblastoma:**<br>-As single treatment:<br>Enhances expression levels of cannabinoid receptor (CB1), and induces p53 phosphorylation [12]<br><br>-In combination:<br>Enhances the effects of a cannabinoid agonist (Win 55212-2 or (-)-11-nor-carboxy-Δ9-THC (THC)) on cancer cells [12]<br><br>**Pancreatic cancer:**<br>-As single treatment:<br>Inhibits cancer cell growth and colony formation [13]<br><br>-In combination:<br>Enhances radiation's effect (radiosensitizer) by inducing oxidative stress, mitochondrial damage, and apoptosis [14]<br><br>**Lung cancer:**<br>-As single treatment:<br>Induces loss of nuclear integrity and upregulates ATM signaling pathway [15]<br><br>**Leukemia cells:**<br>-As single treatment:<br>Activates p53, chk2 and H2AX and induces apoptosis [16]	**Clinical Trial Phase III, IV:**<br>**-**Kaposi's Sarcoma with HIV infection (NCT00444379, NCT01352117)<br><br>**Clinical Trial Phase II:**<br>-Prostate Cancer (NCT00964002)<br>-Pancreatic Cancer (NCT00964171)<br><br>**Clinical Trial Phase I:**<br>-Solid tumors / Non-Hodgkin Lymphoma (NCT01878890)<br><br>***In vitro:***<br>-Glioblastoma cells: T98G [12]<br>-Colorectal carcinoma cells: HCT-15 [12]<br>-Pancreatic cancer cells: BxPC-3, Panc-1 [12, 13] and BxPC-3 [14]<br>-Lung cancer cells: A549 [15]<br>-Leukemia cells: IM9 and HL60 [16]

**Ganciclovir**	**Antiviral**<br>Inhibits viral DNA polymerases [17]	**Prostate cancer with Herpes Virus transduction:**<br>-As single treatment:<br>Induces necrosis and apoptosis, and decreases micro-vessel density [18, 19]<br><br>**Pancreatic cancer with Herpes Virus transduction:**<br>-As single treatment:<br>Decreases cell survival, fragments DNA, and induces apoptosis [20]<br><br>**Thymidine kinase (HSV-TK) transfected cervical cancer:**<br>-As single treatment:<br>Induces cytotoxic effects and apoptosis [21]<br><br>**HSV-TK transfected lung cancer:**<br>-As single treatment:<br>Downregulates cell viability and reduces tumor size [22]	**Clinical Trial Phase II:**<br>-Gene therapy in treating women with refractory or Relapsed Ovarian Epithelial Cancer, Fallopian Tube Cancer, or Peritoneal Cancer (NCT00005025)<br>-Study of Arginine Butyrate and Ganciclovir/Valganciclovir in EBV(+) Lymphoid Malignancies (NCT00917826)<br><br>**Clinical Trial Phase I:**<br>-Brain Tumors (NCT00001328)<br>-Ovarian Cancer (NCT00964756)<br>-Ganciclovir plus Arginine Butyrate in treating patients with cancer or lymphoproliferative disorders associated with the Epstein Barr Virus (NCT00006340)<br>-Gene therapy in treating patients with Primary Brain Tumors (NCT00002824)<br>-Vaccine therapy and Ganciclovir in treating patients with Mesothelioma (NCT00006216)<br><br>***In vivo:***<br>-Prostate cancer cells: RM-1 in male C57BL/6 mice [18, 19]<br>-Cervical cancer cells: SW900 and NCI-H661 in female nude mice [21]<br><br>***In vitro:***<br>-Prostate cancer cells: PC-3, DU145, and RM-1 [18, 19]<br>-Pancreatic cancer cells: SW1990/TK and PA317/TK [20]<br>-Cervical cancer cells: HeLa [21]<br>-Adenocarcinoma cells: Calu-3, NCI-H23, NKI-H1650, A-549, SW-900, NCI-H520, NCI-H661, and NCI-H460 cells [22]

**Indinavir**	**Antiviral**<br>Inhibits HIV protease [23, 24]	**Using computer modeling:**<br>Modulates the alpha-7-nicotinic acetylcholine receptor (pro-carcinogenic protein) and matrix metalloproteinase (MMP)-2 (malignancy promotor)[25]<br><br>**HPV induced cervical cancer:**<br>-As single treatment:<br>Reduces cell viability, decreases secretion of MMP-2 and -9, and induces apoptosis [26]<br><br>**Hepatocarcinoma:**<br>-As single treatment:<br>Inhibits MMP-2 proteolytic activation, delays tumor growth, reduces angiogenesis and induces apoptosis [27]<br><br>**Kaposi's Sarcoma:**<br>-As single treatment:<br>Inhibits angiogenesis and MMP-2 proteolytic activity [28–31]	**Clinical Trial Phase II:**<br>-External-Beam radiation therapy with or without Indinavir and Ritonavir in treating patients with brain metastases (NCT00637637)<br>-Treatment with Indinavir and chemotherapy for Advanced Classical Kaposi's Sarcoma (NCT01067690)<br>-Treatment of classical Non-HIV-Related Kaposi's Sarcoma with the antiviral drug Indinavir (NCT00362310)<br><br>***In vivo:***<br>-Cervical cancer cells HeLa in immunocompromised female C57BL/6 mice [26]<br>-Hepatocarcinoma cells Huh7 and SK-HEP-1 in nude mice [27]<br><br>***In vitro:***<br>-Cervical cancer cells: HeLa [26]<br>-Hepatocarcinoma cells: Huh7 and SK-HEP-1 [27]<br>-Kaposi's Sarcoma cells: HUVECs, HDMVECs, KS, and EA-hy 926 [28]
**Lopinavir**	**Antiviral**<br>HIV-1 and HIV-2 protease inhibitor → antiretroviral combination therapy with Ritonavir [32–35]	**Urological cancer cells:**<br>-In combination with Ritonavir:<br>Induces stress in the endoplasmic reticulum, increases the expression of AMP-activated protein kinase and tumor necrosis factor-related apoptosis-inducing ligand (TRAIL) receptor [36]<br><br>**Lung cancer cells:**<br>-In combination with Ritonavir:<br>Induces cell cycle arrest, downregulates cell viability, and induces apoptosis [15]	**Clinical Trial Phase IV:**<br>-Anti-Retroviral for Kaposi's Sarcoma (NCT00444379)<br><br>**Clinical Trial Phase III:**<br>-AIDS-related Kaposi's Sarcoma (NCT00834457)<br><br>**Clinical Trial Phase II:**<br>-Ritonavir and Lopinavir in treating patients with progressive or recurrent high-grade Glioma (NCT01095094)

**Lopinavir**<br>*(continued)*		**Melanoma cells:**<br>-As single treatment (Lopinavir / Lopinavir-NO):<br>Inhibits cell proliferation, induces morphological changes, increases reactive oxygen species production, and induces induction [37].	***In vivo:***<br>-B16 melanoma cells in C57BL/6 mice [37]<br><br>***In vitro:***<br>-Renal cancer cells (769-P, 786-O) and bladder cancer cells (UMUC-3, T-24) [36]<br>-Lung cancer cells: MRC-5 and A549 [15]<br>-Melanoma cells: B16, B16F10 and A375 [37]
**Maraviroc**	**Anti-Retroviral**<br>Most used CCR5 co-receptor antagonist (interferes with the binding of CCL5 to CCR5) [38]	**Classical Hodgkin Lymphoma:**<br>-As single treatment:<br>Reduces tumor growth and inhibits monocyte accumulation (tumor-associated macrophages (TAMs) ($CD68^+$ cell)) *in vivo* and *in vitro* [39]<br>Inhibits formation and viability of heterospheroids *in vitro* [39]<br><br>-In combination:<br>Synergizes the effects of Doxorubicin(DOX) and Brentuximab Vedotin [39]<br><br>**Pancreatic cancer:**<br>-As single treatment:<br>Downregulates CDK and cyclins and upregulates CDK inhibitors (p21, p27, p18) →G1 cell cycle arrest [40]<br>Leads to remission of pancreatic liver metastasis *in vivo* [40]<br>Increases the expression of cleaved caspases- 3 and -9 and Bax → Induces apoptosis [40]<br><br>**Acute lymphoblastic leukemia (ALL):**<br>-As single treatment:<br>Blocks JAK phosphorylation → inhibits STAT3 activity → Exerts an anti-proliferative effect, induces apoptosis and inhibits the formation of spheroids *in vitro*, also leads to decreased adhesion and migration *in vivo* [41]	**Clinical trial Phase I:**<br>-Metastatic Pancreatic Cancer and Colorectal Cancer in combination with Nivolumab and Ipilimumab (NCT04721301)<br><br>-Colorectal Cancer, Liver metastasis and Neoplasm Metastasis (NCT01736813)<br><br>-Metastatic Colorectal Cancer in combination with Pembrolizumab after failed standard therapy (NCT03274804)<br><br>**Clinical trial Phase II:**<br>-Kaposi's Sarcoma (NCT01276236)

**Maraviroc** *(continued)*		**Colon Cancer:**<br>-As single treatment:<br>Downregulates CCND1 → G1 cell cycle arrest [42]<br>Induces apoptosis via the intrinsic and extrinsic pathways → Induces caspase-3.-7 and -9 cleavage [42]<br><br>**Hepatocellular carcinoma (HCC):**<br>-As single treatment<br>Acts as chemopreventive agent [43]<br><br>**Breast Cancer:**<br>-As single treatment:<br>Inhibits tumor metastasis [44]<br>Reduces the level of CCR5+ Tregs and metastasis formation *in vivo* [45]<br><br>-In combination:<br>Synergizes the effect of anti-VEGF therapy [44]<br>Synergizes the effect of Tocilizumab → Inhibits cell migration [46]<br>Synergizes the effect of anti-IL6 receptor antibody → inhibits tumor growth and metastasis formation *in vivo* [46]<br><br>**Acute myeloid leukemia** (**AML**)**:**<br>-In combination with CD8+ T cells and BX471 (CCR1 antagonist)<br>Inhibits T-reg accumulation and lung metastasis formation and increases the effects of CD8+ T cells in a xenograft model [47]<br><br>**Gastric cancer:**<br>-As single treatment:<br>Reduces the extent of peritoneal disease and increases survival, as well as decreases tumor burden *in vivo* via altered expression of different genes [48]<br><br>More extensive summary is presented in the review by Aldinucci *et al*. [38]	***In vivo:***<br>-Classic Hodgkin lymphoma cells L-540 in athymic nude/nude mice [39]<br>-Classic Hodgkin lymphoma cells L-428 in NSG mice [39]<br>-Human Pancreatic cancer cells Suit2-007 cells in RNU rats [40]<br>-ALL cells SUP-B15 in nude mice [41]<br><br>-HCC model with C57BL/6 mice on a choline-deficient diet [43]<br>-Breast cancer cells MB231 in athymic nude mice[44]<br>-Breast cancer cells MDA-MB-231-LN in athymic nude mice [46]<br><br>-MLL-AF9-induced mouse AML models [47]<br>-Breast cancer cells 4T1, 4T07, and 67NR in BALB/c mice [45]<br>-Gastric cancer cells MKN45 or MKN45 in NOD-SCID mice [48]<br><br>***In vitro:***<br>-Classic Hodgkin lymphoma cells: L-1236, L-428, KM-H2, HDLM-2, and L-540 [39]<br>-Pancreatic cancer cells: Suit2-007 and MIAPaCa-2 [40]<br>-ALL cells: SUP-B15 [41]<br><br>-Colon cancer cells: SW480 and SW620 [42]<br>-Breast cancer cells: MDA-MB-231-LN, SUM149, and SUM159 [46]<br>-Gastric cancer cells: MKN45, MKN74, and KATOIII [48]

**Nelfinavir**	**Anti-Retroviral**<br>HIV-1 protease inhibitor (prevents cleavage of the gag-pol polyprotein → immature virus particles) [49]	**Pancreatic cancer:**<br>-As single treatment:<br>Reduces cell proliferation via cell cycle arrest (phase is cell line specific) and induces apoptosis [50]<br><br>-In combination with Nitroxoline and Erlotinib:<br>Synergizes the effect of Nitroxoline [50]<br><br>**Multiple Myeloma:**<br>-As single treatment:<br>Inhibits 26S proteasome activity [51]<br>Inhibits phosphorylation of AKT, STAT3 and ERK1/2 → induces the pro-apoptotic pathway of the UPR system → caspase-3 cleavage → induces apoptosis and reduces proliferation [51]<br><br>-In combination with Dexamethasone, Bortezomib and Valproic acid<br>Synergizes the effects exerted by Dexamethasone, Bortezomib and Valproic acid [51]<br><br>**Glioblastoma, Myeloma, Laryngeal, Ovarian and Lung Cancer:**<br>-As single treatment:<br>Inhibits proteasome → accumulation of misfolded proteins → ER stress response →induces apoptosis [52–56]<br><br>**Laryngeal and Lung cancer:**<br>-As single treatment:<br>Decreases Sp1 phosphorylation and Sp1 binding to VEGF, decreases HIF-1α induction → decreases VEGF expression → decreases angiogenesis [57]<br><br>-In combination with radiation:<br>Enhances radiation' effect (radiosensitizer) *in vivo* and *in vitro* [53, 57]	**Clinical trial Phase I:**<br>-Cervical Cancer in combination with Cisplatin and radiation (NCT01485731, NCT02363829)<br>-Inoperable Non-Small Cell Lung Cancer (NSCLC) in combination with radiation therapy, Cisplatin, and Etoposide (NCT00589056)<br>-Advanced malignancies in combination with Temsirolimus (NCT01079286)<br>-Pancreatic Cancer in combination with radiation and Gemcitabine (NCT01086332)<br>-Colorectal Cancer in combination with radiation and Capecitabine (NCT00704600)<br>-Lung Cancer in combination with radiation (NCT01447589)<br>-Pancreatic Cancer in combination with Radiation and Gemcitabine Hydrochloride, Leucovorin Calcium, and Fluorouracil (NCT01068327)<br>-Solid tumors (NCT01445106)<br>-Inoperable Vulvar Cancer in combination with Cisplatin and radiation (NCT04169763)<br>-Neoplasms and Lymphoma in combination with MLN9708 (NCT03422874)<br>-Progressive Advanced Hematologic Cancer in combination with Bortezomib (NCT01164709)<br>-Gamma herpesvirus-Related Tumors (NCT02080416)<br>-Glioblastoma in combination with Temozolomide and radiation (NCT00915694, NCT01020292)<br>-Multiple Myeloma in combination with Metformin and Bortezomib (NCT03829020)<br>-Multiple Myeloma in combination with Lenalidomide and Dexamethasone (NCT01555281)<br>-Liposarcoma (NCT00233948)<br>-Glioblastoma in combination with radiochemotherapy (NCT00694837, NCT00694837)

**Nelfinavir**<br>*(continued)*		**Liposarcoma and Prostate cancer :**<br>-As single treatment:<br>Inhibits site-2 protease activity → accumulation of SREBP-1 and ATF6 → ER stress and UPR → induces apoptosis [58, 59]<br><br>**Breast Cancer:**<br>-As single treatment:<br>Inhibits HER2 protein expression and phosphorylation, and inhibits AKT and ERK1/2 signaling via inhibition of HSP90 [60]<br><br>**Myeloma and Lung Cancer:**<br>-In combination with Bortezomib:<br>Synergizes the effect of Bortezomib [54]<br><br>**Thyroid Cancer:**<br>-As single treatment:<br>Induces G0/G1 cell cycle arrest via downregulation of Cyclin D1 and CDK4 [61]<br>Induces apoptosis evidenced by caspase-3 cleavage and inhibits migration [61]<br><br>**Cervical Cancer:**<br>**-**As single treatment:<br>Decreases MnSOD protein expression → increases mitochondrial ROS production → leading to apoptosis and G1 cell cycle arrest [62]	**Clinical trial Phase II:**<br>-Head and Neck Cancer (NCT01065844)<br>-Inoperable NSCLC in combination with radiation and Carboplatin/Paclitaxel and Cisplatin/Etoposide (NCT01108666)<br>-NSCLC in combination with radio-chemotherapy before operation (NCT00791336)<br>-Pancreatic Cancer in combination with Nab-Paclitaxel, Capecitabine, Gemcitabine and radiation (NCT02024009)<br>-Pancreatic Cancer in combination with radiation, Oregovomab, Gemcitabine Hydrochloride, Leucovorin Calcium, and Fluorouracil (NCT01959672)<br><br>-Melanoma, Lung Cancer, or Kidney Cancer in combination with Pembrolizumab, Nivolumab, Atezolizumab and radiation (NCT03050060)<br>-Cervical Dysplasia (NCT01925378)<br>-Multiple Myeloma in combination with Dexamethasone and Bortezomib (NCT02188537)<br>-Kaposi`s sarcoma (NCT00002185, NCT03077451)<br>-Head and Neck Cancer in combination with FMISO and radiation (NCT02207439)<br><br>**Clinical trial Phase III:**<br>-Cervix Carcinoma in combination with radiation and Cisplatin (NCT03256916)<br><br>Published results of clinical trials are found in the following references [63–67]

**Nelfinavir**<br>*(continued)*			***In vivo:***<br>-Myeloma cell line U266-luc cell in NOD/SCID mice [51]<br>-Glioblasoma cells U87 in athymic *nu/nu* mice [53]<br>-Lung cancer cells A549 in Ncr-*nu/nu* mice [57]<br>-Liposarcoma cells LiSa-2 in SCID mice [58]<br>-Breast cancer cells HCC1954, HCC1937, and MDA-MB-231 in BALB/c, nu/nu-NCr mice [60]<br>-Lung cancer cells H157 in athymic NCr-nu/nu mice [54, 55]<br>-Myeloma cells RPMI8226 in NCr-nu/nu mice [54]<br><br>***In vitro:***<br>-Pancreatic cancer cells: AsPC-1, Capan-2 and BxPC-3 PC [50]<br>-Myeloma cell lines: U266, MM1S, RPMI, OPM2, LP1 and fresh plasma cells [51], RPMI8226 and L363 [54]<br>-Glioblastoma cell lines: U251, LN229, T98G and U87 [52]<br>-Laryngeal cancer cells: SQ20B [53, 57]<br>-Lung cancer cells: A549 [54, 55, 57] and H157 [54, 55]<br>-Liposarcoma cells: SW872 and LiSa-2 [58, 68]<br>-Breast cancer cells: HCC1143, HCC1395, HCC1937, HCC1954, HCC2218, MCF-7, BT474, and HCC38 [60], MCF-7, SKBR-3, MCF-7/LCC2, SKBR-3/Her10, JIMT-1, and BT474 [55]<br>-Thyroid cancer cell lines: FTC133, BCPAP and SW1736 [61]<br>-Prostate cancer cells: DU145, PC-3 CR-PC cells and AD-PC LNCaP FGC [59]<br>-Ovarian cancer cells: SKOV3, OV-GH-5, OVCAR3, OV-GH-1 and patient probes [56]<br>-Cervical cancer cell lines: Hela, SiHa and CaSki [62]

**Ribavirin**	**Broad spectrum anti-viral** (Hepatitis C in combination with interferon-α)<br>Mode of action not yet fully understood, but different modes of actions proposed: immunomodulatory properties<br><br>inhibition of the Inosinmonophosphate-Dehydrogenase (IMPDH)<br><br>direct inhibition of the HCV-encoded NS5B RNA polymerase<br><br>induction of lethal mutagenesis modulation of interferon-stimulated gene expression [69]	**Pharyngeal, Ovarian and Breast Cancer and ALL:**<br>-As single treatment:<br>Inhibits eIF4E → impedes mRNA export → decreases NBS1 expression → decreased Akt activation and production of Akt downstream target [70–73] and intersects eIF4/p70S6K pathway signaling [70, 71] → decreases proliferation and colony formation<br><br>**ALL:**<br>-In combination<br>Enhances the antiproliferative effects of L-asparaginases, Cytarabines, Dexamethasones, Etoposides, Doxorubicins and Vincristines [71]<br><br>**Nasopharyngeal carcinoma (NPC):**<br>-As single treatment:<br>Exerts anti-proliferative effect via G_0 cell cycle arrest, inhibits migration and invasion, and modulates EZH2, Snail, eIF4E, and IMPDH expression [74]<br><br>-In combination with radiation:<br>Enhances radiation's effect (radiosensitizer) [74]<br><br>**Glioma and Osteosarcoma:**<br>-As single treatment:<br>Exerts anti-proliferative effect via G_0 cell cycle arrest and apoptosis induction via intrinsic and extrinsic pathways [75, 76]<br>Decreases eIF4E phosphorylation on serine 209 (S209) → decreases protein synthesis →decreases proliferation [77, 78]<br>inhibits EZH2, ERK phosphorylation and Snail → decreased migration and adhesion [77, 78]<br><br>-In combination<br>Enhances the effects of Temozolomide and irradiation [78]	**Clinical trial Phase I:**<br>-Metastatic Breast Cancer (NCT01056757)<br>-Head and Neck Cancer in combination with Afatinib, and Carboplatin/Paclitaxel (NCT01721525)<br>-Malignant Solid Tumors (NCT01309490)<br>-HCC in combination with Pembrolizumab and Elbasvir/Grazoprevir (NCT02940496)<br>-AML in combination with Cytarabine Arabinoside (NCT01056523)<br>-AML in combination with Brequinar (NCT03760666)<br>-AML in combination with Decitabine (NCT02109744)<br>-Lymphoma (NCT03585725)<br><br>**Clinical trial Phase II:**<br>-AML (NCT00559091)<br>-AML in combination with Vismodegib and/or Decitabine (NCT02073838)<br><br>**Clinical trial Phase IV:**<br>-In combination with interferon-alfa-2b after resection of primary HCC (NCT00375661)<br>-In combination with Edipasvir/Sofosbuvir after resection of primary HCC (NCT02959359)<br><br>**Undefines Phase:**<br>-(HPV)-Related Malignancies (NCT02308241)<br>-Head and Neck Cancer (NCT01268579)<br><br>Published results of clinical trials are found in the following references [85, 86]

**Ribavirin**<br>*(continued)*		**Renal Cell Carcinoma (RCC):**<br>-As single treatment:<br>Exerts anti-proliferative effect via $G_{02/M}$ cell cycle arrest [79], inhibits migration and invasion, and exerts immunmodulatory effects in tumor microenvironment: reduces IL-10 production and increases TGF-β secretion [79]<br><br>**AML:**<br>-As single treatment:<br>Reduces eIF4E activity → downregulates eIF4E mRNA export and translation targets (c-Myc, XIAP, Cyclin E and NBS 1) [80]<br><br>-In combination:<br>Synergizes the effects of Azacytidine, Sorafenib, Ara-C, Idarubicin and leads to decreased colony number [80]<br><br>**Osteosarcoma and HCC:**<br>-In combination:<br>Synergizes the effect of DOX *in vivo* and *in vitro* [77, 81]<br><br>**Leukemia:**<br>-As single treatment:<br>Downregulates the phosphorylation of mTOR/eIF4E and ERK/Mnk1/eIF4E signaling pathways, inhibits the assembly of eIF4F translation initiation complexes, and reduces translation of onco-protein Mcl-1 [82]<br><br>-In combination:<br>Synergizes the effect of Imatinib [82]<br><br>**Oral Tongue Squamous Cell Carcinoma (OTSCC) and HCC:**<br>-As single treatment:<br>Suppresses eIF4E phosphorylation → suppresses Akt, mTOR and 4EBP1 phosphorylation → exerts anti-proliferative and pro-apoptotic effect [81, 83]	***In vivo***<br>-NPC cell lines C666-1 and CNE-2 in NU/NU athymic mice [74]<br>-Glioblastoma cells 9L in F433 rats [78]<br>-Glioblastoma cells GB1A in NU/NU athymic mice [78]<br>-Osteosarcoma cell lines MG-63 in SCID mice [77]<br>-Infant ALL cells PAPAWG, PAPMGS, 771–206 in NSG mouse model [71]<br>-OTSCC cell lines SCC-9 and CAL27 in SCID mice [83]<br>-Ovarian cancer cells OVCAR3 in SCID mice [70]<br>-HCC cell line HepG2 in NOD/SCID mice [81]<br><br>***In vitro***<br>-Pharyngeal cancer cells: FaDu ***[73]***<br>-NPC cell lines: C666-1, CNE-2, HNE-1, HONE-1, and SUNE-1 [74]<br>-Glioma cell lines: A-172, AM-38, T98G, U-87MG, U-138MG, U-251MG and YH-13 [75], U87MG and U138MG [76], U87, U251, LN18, SF767, T98G, 9L, F98, C6, and GB1A[78]<br>-RCC cells Renca and 786–0 [79]<br>-Primary AML cells [80]<br>-ALL cancer cell lines: RS4:11, SEM-K2, KOPN-8 and infant ALL patient samples [71]<br>-Osteosarcoma cell lines: MG-63, U-2 OS, OB-6 and BJ-5ta [77]<br>-Breast cancer cells: MCF-7, MDA-MB-468, MDA-MB-321, ZR75.1, BT474 and SkBr3, patient-derived samples [72]<br>-Leukemia cell lines: SUP-B15 and K562 and primary leukemic specimen [82]<br>-OTSCC cell lines: SCC-9 and CAL27 [83]<br>-Ovarian cancer cells: ES2, OVCAR3, TOV-21G, SKOV3, PEO1, COV504, OV56, PEA1 [70]<br>-HCC cell lines: HepG2, HepG3, HuH6, and SNU-182 [81]

**Ribavirin** *(continued)*		**OTSCC:**<br>-In combination:<br>Synergizes the effect of Paclitaxel (PAX) [83]<br><br>**Ovarian Cancer:**<br>-In combination:<br>Synergizes the effect of Cisplatin [70]<br><br>More extensive review is presented in the review by Casaos et al. [84]	
**Ritonavir**	**Anti-retroviral**<br>HIV-1 protease inhibitor (prevents cleavage of the gag-pol polyprotein → immature virus particles) [87]	**T-cell leukemia:**<br>-As single treatment:<br>Inhibits NF-κB transcriptional activity → downregulates the expression of NF-κB-regulated gene products (Bcl-XL, survivin, cyclin D2, and c-Myc) → anti-proliferative and pro-apoptotic effect [88]<br>Activates the chymotrypsin-like activity of isolated 26S proteasomes [89]<br>Inhibits proteolytic degradation → accumulation of p21 → cell cycle dysregulation and apoptosis [89]<br><br>**Myelotic Leukemia:**<br>-In combination<br>Enhances the anti-proliferative effect of ATRA and enhances the induction of differentiation [90]<br><br>**Ovarian, Pancreatic, Lung and Breast Cancer:**<br>-As single treatment:<br>Inhibits cell motility and invasiveness [91–94]<br>Decreases phosphorylation of AKT → apoptosis [91–93]<br>Binds to Hsp90 → decreases RB phosphorylation and expression of cyclins and CDK, as well as upregulates expression of CDKI →G1 cell cycle arrest [91–94]<br><br>-In combination :	**Clinical trial Phase I:**<br>-Breast Cancer before surgery (NCT01009437)<br>-Prostate Cancer in combination with DOX (NCT03136640)<br>-Neoplasm Metastasis in combination with DS-8201a (NCT03383692)<br>-Prostatic Neoplasms in combination with DOX, hormonal therapy and radiation (NCT03066154)<br>-Different Cancers in combination with DOX (NCT01173913, NCT03150368, NCT03147378)<br>-Multiple Myeloma and CLL in combination with Metformin Hydrochloride (NCT02948283)<br>-Glioblastoma in combination with 8 other repurposed drugs (NCT02770378)<br><br>**Clinical trial Phase II:**<br>-Different cancers in combination with Indinavir Sulfate and radiation (NCT00637637)<br>-Breast Cancer in combination with DOX (NCT03890744)<br>-Prostate Cancer in combination with DOX (NCT04028388)<br>-Brain Cancers in combination with lopinavir (NCT01095094)<br>-Kaposi`s Sarcoma (NCT00002366)<br>-Kaposi`s Sarcoma in combination with Abacavir and lopinavir (NCT00834457)<br>**Clinical trial Phase IV:**

**Ritonavir** *(continued)*		Induces additive effect in combination with PAX [92]<br>Enhances Gemcitabines' effect [93]<br><br>**Lung cancer**:<br>-As single treatment:<br>Decreases survivin expression, phosphorylation of c-Src and STAT3 → cell cycle arrest and apoptosis [94]<br>Increases PARP cleavage → apoptosis induction [94]<br><br>-In combination:<br>Exerts additive effect with Gemcitabine and/or Cisplatin [94]<br><br>**Kaposi`s Sarcoma:**<br>-As single treatment:<br>Inhibits NF-κB activation → decreases production of NF-κB–dependent cytokines (IL-6, IL-8, and TNF-α) and decreases expression of INF-γ, TNF-α, IL-1β, and IL-6 → may lead to decreased angiogenesis, inflammation, and immune cell infiltration [95]<br>Inhibits ICAM-1, VCAM-1, and E-selectin expression → decreases leukocyte adhesion [95]<br><br>**Multiple Myeloma:**<br>-In combination:<br>Enhances the pro-apoptotic effect of Metformin [96]<br>Suppresses the expression of p-AKT, p-AMPK, pmTORC1 and MCL-1 *in vitro* and *in vivo* [96]<br><br>**Prostate cancer:**<br>-As single treatment:<br>Inhibits DNA binding activity of NFκB [98]<br><br>-In combination<br>Inhibits DOX induced induction of CYP3A4 expression *in vivo* and *in vitro* [98]<br>Enhances DOX anti-proliferative and pro-apoptotic effect *in vivo*	-Kaposi`s Sarcoma in combination with Lopinavir plus Emtricitabine/Tenofovir<br><br>***In vivo***<br>-Primary T-cell leukemia cells in NOG mice [88]<br>-Breast cancer cell line MDA-MB-231 in nude mice [91]<br>-Kaposi's Sarcoma cell line KSIMM in BNX mice [95]<br>-Multiple Myeloma cell line KMS11-GFP in NOD/SCID CB17 mice [96]<br>-Glioma cell line 9L in Fischer rats [97]<br>-Prostate cancer cell line DU145 in triple immunodeficient BNX *nu/nu* mice [98]<br>-T-cell leukemia cells EL4 in C57BL/6 mice [89]<br><br>***In vitro***<br>-T-cell leukemia cell line: Jurkat, K562, patient-derived samples [88], EL4, T1 and Jurkat cells [89]<br>-Myelocytic leukemia cell lines :HL-60, NB4 and UF-1 [90]<br>-Ovarian cancer cell lines: MDAH-2774 and SKOV-3 [92]<br>-Breast cancer cell lines: MCF7, T47D, MDA-MB-231 and MDA-MB-436 [91]<br>-Pancreatic tumor cell lines: BxPC-3, MIA PaCa-2, and PANC-1 [93]<br>-Lung cancer cell lines: A549, H522, H23 and H838<br>-Kaposi`s Sarcoma cell line: KSIMM [95]<br>-Multiple Myeloma cell lines: KMS11, L363, JJN3 and patient-derived samples [96]<br>-Glioma cell lines: GL15 and 9L [97]<br>-Prostate cancer cell lines: PC-3 and DU145 [98]

**Ritonavir** *(continued)*		and *in vitro* [98]<br><br>**Glioma:**<br>-As single treatment:<br>Inhibits the chymotrypsin-like activity of the proteasome → G_1 cell cycle arrest and apoptosis [97]	
**Zidovudine / Azidothymidine**	**Anti-Retroviral**<br>Thymidine analogue (phosphorylated AZT is incorporated into viral DNA where it acts as a false substitute for viral reverse transcription and blocks chain elongation) [99, 100]	**Esophageal, Colorectal, Breast, Parathyroid and Ovarian Cancer, HCC:**<br>-As single treatment:<br>Reduces telomerase activity [99, 101–104]<br>Induces S and G2/M cell cycle arrest [99, 101, 103, 105]<br>Causes DNA damage and increased expression of γ-H2A and pChk2 [99]<br><br>**HCC:**<br>-As single treatment:<br>Induces apoptosis, decreases Chk1 and CHK2 expression and upregulates phosphorylated Chk1 and CHK2 [103]<br><br>**Head and Neck cancer:**<br>-In combination:<br>Induces mitochondrial dysfunction → disrupts thiol metabolism and causes oxidative stress → increased cytotoxicity in combination with Cisplatin [106]<br>Synergizes the effects of PAX [107]<br><br>**Breast Cancer:**<br>-As single treatment:<br>Suppresses hTERT and c-Myc → suppresses hTER, Mad1 and hTEP-1 → accelerates telomere loss and apoptosis [108]<br>Anti-proliferative effect [105, 109]<br>**Colorectal and Gastric Cancer:**<br>-In combination:	**Clinical trial Phase I:**<br>-Kaposi`s Sarcoma in combination with Interferon (IFN) α 2a or IFN- α (NCT00001113, NCT00000725)<br>-Kaposi`s Sarcoma in combination with Sargramostim and IFN-A2a (NCT00000694)<br><br>**Clinical trial Phase II:**<br>-Lymphoma in combination with Methotrexate and radiation (NCT00003261)<br>-Lymphoma in combination with Bortezomib effusion drainage, Bevacizumab, and combination chemotherapy (NCT00217503)<br>-Kaposi`s Sarcoma in combination with Abacavir (NCT00834457)<br>-Lymphoma in combination with combination chemotherapy, radiation therapy, and antiviral therapy (NCT00002571)<br>-T-Cell Leukemia and Lymphoma in combination with chemotherapy and IFN-α (NCT00041327)<br>-Non-Hodgkin Lymphoma in combination with combination chemotherapy (NCT01964755)<br>-Kaposi`s Sarcoma in combination with IFN- α 2a (NCT00000687)<br><br>**Clinical trial Phase IV:**<br>-Lymphoma in combination with IFN-α-2b and PEG-IFN α-2b (NCT00854581)

		Synergizes the effect of 5-FU [102, 110]	
**Zidovudine / Azidothymidine** *(continued)*		**Gastric cancer:**<br>-In combination:<br>Synergizes the effects of FA-2-b-β [111]<br>Upregulates caspase-3 mRNA and downregulates BCL-2 expression [111]<br><br>**Glioma:**<br>-In combination with radiation (as radiosensitizer)<br>Inhibits the irradiation activated telomerase activity → decreased restore rate of shortened telomere, decreased repair rate of DNA strand breaks, and increased radiosensitivity [112]	**Unknown Phase:**<br>-Non-Hodgkin Lymphoma in combination with Methotrexate and Dexamethasone (NCT00000723)<br>-Lymphoma in combination with combination chemotherapy-radiation (NCT00000703)<br><br>Published results of clinical trials are found in the following references [113]<br><br>***In vivo:***<br>-MNU-induced breast cancer model in Sprague Dawley rats [109]<br>-Gastric cancer cell line MKN28 in nude mice [110]<br><br>***In vitro:***<br>-Esophageal cell line: TE-11 [99]<br>-Head and Neck cancer cells: FaDu [107], Cal-27 and SQ20B [106]<br>-Breast cancer cell lines: MCF-7 [108, 109], T47D [105]<br>-Ovarian cancer cell line: HO-8910 [101]<br>-Colorectal cancer cell line: HT-29 [102]<br>-Gastric cancer cells: MKN45 [111]<br>-HCC cells: HepG2 [103]<br>-Primary cultures of human parathyroid cells [104]<br>-Glioma cells U251 [112]

Abbreviations: Acute lymphoblastic leukemia (ALL), Acute myeloid leukemia (AML) ,Chronic lymphocytic leukemia (CLL), doxorubicin (DOX), Hepatocellular carcinoma (HCC), Inosinmonophosphate-Dehydrogenase (IMPDH), manganese superoxide dismutase (MnSOD), Nasopharyngeal carcinoma (NPC), Non-Small Cell Lung Cancer (NSCLC), telomerase RNA (hTER), telomerase reverse transcriptase (hTERT), tumor-associated macrophages (TAMs), unfolded protein response (UPR), phosphorylated checkpoint kinase 2 (pChk2), Paclitaxel (PAX), oral tongue squamous cell carcinoma (OTSCC).

References

[1] Uhnoo, I.; Linde, A.; Pauksens, K.; Lindberg, A.; Eriksson, M.; Norrby, R. Treatment and prevention of influenza: Swedish recommendations. *Scandinavian journal of infectious diseases*, **2003**, *35*, 3–11.
[2] Jefferson, T.; Demicheli, V.; Rivetti, D.; Jones, M.; Di Pietrantonj, C.; Rivetti, A. Antivirals for influenza in healthy adults: systematic review. *The Lancet*, **2006**, *367*, 303–313.
[3] https://www.gelbe-liste.de/wirkstoffe/Amantadin_21448. Amantadin.
[4] Lea, A.P.; Bryson, H.M. Cidofovir. *Drugs*, **1996**, *52*, 225-230; discussion 231.
[5] Hadaczek, P.; Ozawa, T.; Soroceanu, L.; Yoshida, Y.; Matlaf, L.; Singer, E.; Fiallos, E.; James, C.D.; Cobbs, C.S. Cidofovir: a novel antitumor agent for glioblastoma. *Clinical cancer research : an official journal of the American Association for Cancer Research*, **2013**, *19*, 6473–6483.
[6] Schutter, T. de; Andrei, G.; Topalis, D.; Naesens, L.; Snoeck, R. Cidofovir selectivity is based on the different response of normal and cancer cells to DNA damage. *BMC medical genomics*, **2013**, *6*, 18.
[7] Yang, Y.; Zhao, X.; Chen, W.; Gao, Z.; Liu, A.; Guo, J.; Yan, Z.; Dou, Y.; Wang, H.; Li, Y. Effects of cidofovir on human papillomavirus-positive cervical cancer cells xenografts in nude mice. *Oncology research*, **2010**, *18*, 519–527.
[8] Deutsch, E.; Haie-Meder, C.; Bayar, M.A.; Mondini, M.; Laporte, M.; Mazeron, R.; Adam, J.; Varga, A.; Vassal, G.; Magné, N.; Chargari, C.; Lanoy, E.; Pautier, P.; Levy, A.; Soria, J.-C. Phase I trial evaluating the antiviral agent Cidofovir in combination with chemoradiation in cervical cancer patients. *Oncotarget*, **2016**, *7*, 25549–25557.
[9] Catalani, S.; Palma, F.; Battistelli, S.; Nuvoli, B.; Galati, R.; Benedetti, S. Reduced cell viability and apoptosis induction in human thyroid carcinoma and mesothelioma cells exposed to cidofovir. *Toxicology in vitro : an international journal published in association with BIBRA*, **2017**, *41*, 49–55.
[10] https://www.ratiopharm.de/assets/products/de/label/Efavirenz%20Teva%20600%20mg%20Filmtabletten%20-%203.pdf?pzn=6990133. Efavirenz, **May 23, 2021**.
[11] Adkins, J.C., Noble, S. Efavirenz. Efavirenz. *Drugs*, **1998,** 1055–1064.
[12] Hecht, M.; Harrer, T.; Büttner, M.; Schwegler, M.; Erber, S.; Fietkau, R.; Distel, L.V. Cytotoxic effect of efavirenz is selective against cancer cells and associated with the cannabinoid system. *AIDS (London, England)*, **2013**, *27*, 2031–2040.
[13] Hecht, M.; Erber, S.; Harrer, T.; Klinker, H.; Roth, T.; Parsch, H.; Fiebig, N.; Fietkau, R.; Distel, L.V. Efavirenz Has the Highest Anti-Proliferative Effect of Non-Nucleoside Reverse Transcriptase Inhibitors against Pancreatic Cancer Cells. *PloS one*, **2015**, *10*, e0130277.
[14] Hecht, M.; Harrer, T.; Körber, V.; Sarpong, E.O.; Moser, F.; Fiebig, N.; Schwegler, M.; Stürzl, M.; Fietkau, R.; Distel, L.V. Cytotoxic effect of Efavirenz in BxPC-3 pancreatic cancer cells is based on oxidative stress and is synergistic with ionizing radiation. *Oncology letters*, **2018**, *15*, 1728–1736.
[15] Marima, R.; Hull, R.; Dlamini, Z.; Penny, C. Efavirenz and Lopinavir/Ritonavir Alter Cell Cycle Regulation in Lung Cancer. *Frontiers in oncology*, **2020**, *10*, 1693.
[16] Brüning, A.; Jückstock, J.; Kost, B.; Tsikouras, P.; Weissenbacher, T.; Mahner, S.; Mylonas, I. Induction of DNA damage and apoptosis in human leukemia cells by efavirenz. *Oncology reports*, **2017**, *37*, 617–621.
[17] Crumpacker, C.S. Ganciclovir. *The New England journal of medicine*, **1996**, *335*, 721–729.
[18] Eastham, J.A.; Chen, S.H.; Sehgal, I.; Yang, G.; Timme, T.L.; Hall, S.J.; Woo, S.L.; Thompson, T.C. Prostate cancer gene therapy: herpes simplex virus thymidine kinase gene transduction followed by ganciclovir in mouse and human prostate cancer models. *Human gene therapy*, **1996**, *7*, 515–523.
[19] Ayala, G.; Satoh, T.; Li, R.; Shalev, M.; Gdor, Y.; Aguilar-Cordova, E.; Frolov, A.; Wheeler, T.M.; Miles, B.J.; Rauen, K.; Teh, B.S.; Butler, E.B.; Thompson, T.C.; Kadmon, D. Biological response determinants in HSV-tk + ganciclovir gene therapy for prostate cancer. *Molecular therapy : the journal of the American Society of Gene Therapy*, **2006**, *13*, 716–728.

[20] Wang, J.; Lu, X.-X.; Chen, D.-Z.; Li, S.-F.; Zhang, L.-S. Herpes simplex virus thymidine kinase and ganciclovir suicide gene therapy for human pancreatic cancer. *World journal of gastroenterology*, **2004**, *10*, 400–403.
[21] Shao, D.; Zeng, Q.; Fan, Z.; Li, J.; Zhang, M.; Zhang, Y.; Li, O.; Chen, L.; Kong, X.; Zhang, H. Monitoring HSV-TK/ganciclovir cancer suicide gene therapy using CdTe/CdS core/shell quantum dots. *Biomaterials*, **2012**, *33*, 4336–4344.
[22] Määttä, A.-M.; Tenhunen, A.; Pasanen, T.; Meriläinen, O.; Pellinen, R.; Mäkinen, K.; Alhava, E.; Wahlfors, J. Non-small cell lung cancer as a target disease for herpes simplex type 1 thymidine kinase - ganciclovir gene therapy. *Int J Oncol*, **2004**.
[23] Piscitelli, S.C.; Burstein, A.H.; Chaitt, D.; Alfaro, R.M.; Falloon, J. Indinavir concentrations and St John's wort. *The Lancet*, **2000**, *355*, 547–548.
[24] Plosker, G.L.; Noble, S. Indinavir: a review of its use in the management of HIV infection. *Drugs*, **1999**, *58*, 1165–1203.
[25] Lee, A.; Saito, E.; Ekins, S.; McMurtray, A. Extracellular binding of indinavir to matrix metalloproteinase-2 and the alpha-7-nicotinic acetylcholine receptor: implications for use in cancer treatment. *Heliyon*, **2019**, *5*, e02526.
[26] Sharma, S.; Baksi, R.; Agarwal, M. Repositioning of anti-viral drugs as therapy for cervical cancer. *Pharmacological reports : PR*, **2016**, *68*, 983–989.
[27] Esposito, V.; Palescandolo, E.; Spugnini, E.P.; Montesarchio, V.; Luca, A. de; Cardillo, I.; Cortese, G.; Baldi, A.; Chirianni, A. Evaluation of antitumoral properties of the protease inhibitor indinavir in a murine model of hepatocarcinoma. *Clinical cancer research : an official journal of the American Association for Cancer Research*, **2006**, *12*, 2634–2639.
[28] Sgadari, C.; Barillari, G.; Toschi, E.; Carlei, D.; Bacigalupo, I.; Baccarini, S.; Palladino, C.; Leone, P.; Bugarini, R.; Malavasi, L.; Cafaro, A.; Falchi, M.; Valdembri, D.; Rezza, G.; Bussolino, F.; Monini, P.; Ensoli, B. HIV protease inhibitors are potent anti-angiogenic molecules and promote regression of Kaposi sarcoma. *Nature medicine*, **2002**, *8*, 225–232.
[29] Sgadari, C.; Monini, P.; Barillari, G.; Ensoli, B. Use of HIV protease inhibitors to block Kaposi's sarcoma and tumour growth. *The Lancet Oncology*, **2003**, *4*, 537–547.
[30] Monini, P.; Sgadari, C.; Toschi, E.; Barillari, G.; Ensoli, B. Antitumour effects of antiretroviral therapy. *Nature reviews. Cancer*, **2004**, *4*, 861–875.
[31] Chow, W.A.; Jiang, C.; Guan, M. Anti-HIV drugs for cancer therapeutics: back to the future? *The Lancet Oncology*, **2009**, *10*, 61–71.
[32] Cvetkovic, R.S.; Goa, K.L. Lopinavir/ritonavir: a review of its use in the management of HIV infection. *Drugs*, **2003**, *63*, 769–802.
[33] Oldfield, V.; Plosker, G.L. Lopinavir/ritonavir: a review of its use in the management of HIV infection. *Drugs*, **2006**, *66*, 1275–1299.
[34] Brower, E.T.; Bacha, U.M.; Kawasaki, Y.; Freire, E. Inhibition of HIV-2 protease by HIV-1 protease inhibitors in clinical use. *Chemical biology & drug design*, **2008**, *71*, 298–305.
[35] https://www.gelbe-liste.de/wirkstoffe/Lopinavir_44754. Lopinavir, **May 18, 2021**.
[36] Okubo, K.; Isono, M.; Asano, T.; Sato, A. Lopinavir-Ritonavir Combination Induces Endoplasmic Reticulum Stress and Kills Urological Cancer Cells. *Anticancer research*, **2019**, *39*, 5891–5901.
[37] Paskas, S.; Mazzon, E.; Basile, M.S.; Cavalli, E.; Al-Abed, Y.; He, M.; Rakocevic, S.; Nicoletti, F.; Mijatovic, S.; Maksimovic-Ivanic, D. Lopinavir-NO, a nitric oxide-releasing HIV protease inhibitor, suppresses the growth of melanoma cells in vitro and in vivo. *Investigational new drugs*, **2019**, *37*, 1014–1028.
[38] Aldinucci, D.; Borghese, C.; Casagrande, N. The CCL5/CCR5 Axis in Cancer Progression. *Cancers*, **2020**, *12*.
[39] Casagrande, N.; Borghese, C.; Visser, L.; Mongiat, M.; Colombatti, A.; Aldinucci, D. CCR5 antagonism by maraviroc inhibits Hodgkin lymphoma microenvironment interactions and xenograft growth. *Haematologica*, **2019**, *104*, 564–575.
[40] Huang, H.; Zepp, M.; Georges, R.B.; Jarahian, M.; Kazemi, M.; Eyol, E.; Berger, M.R. The CCR5 antagonist maraviroc causes remission of pancreatic cancer liver metastasis in nude rats based on cell cycle inhibition and apoptosis induction. *Cancer Letters*, **2020**, *474*, 82–93.
[41] Zi, J.; Yuan, S.; Qiao, J.; Zhao, K.; Xu, L.; Qi, K.; Xu, K.; Zeng, L. Treatment with the C-C chemokine receptor type 5 (CCR5)-inhibitor maraviroc suppresses growth and induces apoptosis of acute lymphoblastic leukemia cells. *American Journal of Cancer Research*, **2017**, *7*, 869–880.
[42] Pervaiz, A.; Ansari, S.; Berger, M.R.; Adwan, H. CCR5 blockage by maraviroc induces cytotoxic and apoptotic effects in colorectal cancer cells. *Medical oncology (Northwood, London, England)*, **2015**, *32*, 158.
[43] Ochoa-Callejero, L.; Pérez-Martínez, L.; Rubio-Mediavilla, S.; Oteo, J.A.; Martínez, A.; Blanco, J.R. Maraviroc, a CCR5 antagonist, prevents development of hepatocellular carcinoma in a mouse model. *PloS one*, **2013**, *8*, e53992.

[44] Lee, E.; Fertig, E.J.; Jin, K.; Sukumar, S.; Pandey, N.B.; Popel, A.S. Breast cancer cells condition lymphatic endothelial cells within pre-metastatic niches to promote metastasis. *Nature communications*, **2014**, *5*, 4715.

[45] Halvorsen, E.C.; Hamilton, M.J.; Young, A.; Wadsworth, B.J.; LePard, N.E.; Lee, H.N.; Firmino, N.; Collier, J.L.; Bennewith, K.L. Maraviroc decreases CCL8-mediated migration of CCR5(+) regulatory T cells and reduces metastatic tumor growth in the lungs. *Oncoimmunology*, **2016**, *5*, e1150398.

[46] Jin, K.; Pandey, N.B.; Popel, A.S. Simultaneous blockade of IL-6 and CCL5 signaling for synergistic inhibition of triple-negative breast cancer growth and metastasis. *Breast cancer research : BCR*, **2018**, *20*, 54.

[47] Wang, R.; Feng, W.; Wang, H.; Wang, L.; Yang, X.; Yang, F.; Zhang, Y.; Liu, X.; Zhang, D.; Ren, Q.; Feng, X.; Zheng, G. Blocking migration of regulatory T cells to leukemic hematopoietic microenvironment delays disease progression in mouse leukemia model. *Cancer Letters*, **2020**, *469*, 151–161.

[48] Mencarelli, A.; Graziosi, L.; Renga, B.; Cipriani, S.; D'Amore, C.; Francisci, D.; Bruno, A.; Baldelli, F.; Donini, A.; Fiorucci, S. CCR5 Antagonism by Maraviroc Reduces the Potential for Gastric Cancer Cell Dissemination. *Translational oncology*, **2013**, *6*, 784–793.

[49] Lv, Z.; Chu, Y.; Wang, Y. HIV protease inhibitors: a review of molecular selectivity and toxicity. *HIV/AIDS (Auckland, N.Z.)*, **2015**, *7*, 95–104.

[50] Veschi, S.; Lellis, L. de; Florio, R.; Lanuti, P.; Massucci, A.; Tinari, N.; Tursi, M. de; Di Sebastiano, P.; Marchisio, M.; Natoli, C.; Cama, A. Effects of repurposed drug candidates nitroxoline and nelfinavir as single agents or in combination with erlotinib in pancreatic cancer cells. *Journal of Experimental & Clinical Cancer Research : CR*, **2018**, *37*, 236.

[51] Bono, C.; Karlin, L.; Harel, S.; Mouly, E.; Labaume, S.; Galicier, L.; Apcher, S.; Sauvageon, H.; Fermand, J.-P.; Bories, J.-C.; Arnulf, B. The human immunodeficiency virus-1 protease inhibitor nelfinavir impairs proteasome activity and inhibits the proliferation of multiple myeloma cells in vitro and in vivo. *Haematologica*, **2012**, *97*, 1101–1109.

[52] Pyrko, P.; Kardosh, A.; Wang, W.; Xiong, W.; Schönthal, A.H.; Chen, T.C. HIV-1 protease inhibitors nelfinavir and atazanavir induce malignant glioma death by triggering endoplasmic reticulum stress. *Cancer research*, **2007**, *67*, 10920–10928.

[53] Gupta, A.K.; Li, B.; Cerniglia, G.J.; Ahmed, M.S.; Hahn, S.M.; Maity, A. The HIV protease inhibitor nelfinavir downregulates Akt phosphorylation by inhibiting proteasomal activity and inducing the unfolded protein response. *Neoplasia (New York, N.Y.)*, **2007**, *9*, 271–278.

[54] Kawabata, S.; Gills, J.J.; Mercado-Matos, J.R.; Lopiccolo, J.; Wilson, W.; Hollander, M.C.; Dennis, P.A. Synergistic effects of nelfinavir and bortezomib on proteotoxic death of NSCLC and multiple myeloma cells. *Cell death & disease*, **2012**, *3*, e353.

[55] Gills, J.J.; LoPiccolo, J.; Tsurutani, J.; Shoemaker, R.H.; Best, C.J.M.; Abu-Asab, M.S.; Borojerdi, J.; Warfel, N.A.; Gardner, E.R.; Danish, M.; Hollander, M.C.; Kawabata, S.; Tsokos, M.; Figg, W.D.; Steeg, P.S.; Dennis, P.A. Nelfinavir, A lead HIV protease inhibitor, is a broad-spectrum, anticancer agent that induces endoplasmic reticulum stress, autophagy, and apoptosis in vitro and in vivo. *Clinical cancer research : an official journal of the American Association for Cancer Research*, **2007**, *13*, 5183–5194.

[56] Brüning, A.; Burger, P.; Vogel, M.; Rahmeh, M.; Gingelmaiers, A.; Friese, K.; Lenhard, M.; Burges, A. Nelfinavir induces the unfolded protein response in ovarian cancer cells, resulting in ER vacuolization, cell cycle retardation and apoptosis. *Cancer biology & therapy*, **2009**, *8*, 226–232.

[57] Pore, N.; Gupta, A.K.; Cerniglia, G.J.; Jiang, Z.; Bernhard, E.J.; Evans, S.M.; Koch, C.J.; Hahn, S.M.; Maity, A. Nelfinavir down-regulates hypoxia-inducible factor 1alpha and VEGF expression and increases tumor oxygenation: implications for radiotherapy. *Cancer research*, **2006**, *66*, 9252–9259.

[58] Guan, M.; Fousek, K.; Jiang, C.; Guo, S.; Synold, T.; Xi, B.; Shih, C.-C.; Chow, W.A. Nelfinavir induces liposarcoma apoptosis through inhibition of regulated intramembrane proteolysis of SREBP-1 and ATF6. *Clinical cancer research : an official journal of the American Association for Cancer Research*, **2011**, *17*, 1796–1806.

[59] Guan, M.; Fousek, K.; Chow, W.A. Nelfinavir inhibits regulated intramembrane proteolysis of sterol regulatory element binding protein-1 and activating transcription factor 6 in castration-resistant prostate cancer. *The FEBS journal*, **2012**, *279*, 2399–2411.

[60] HIV Drug Shows Efficacy in Treating Mouse Models of HER2+ Breast Cancer. *JNCI Journal of the National Cancer Institute*, **2012**, *104*, NP-NP.

[61] Jensen, K.; Bikas, A.; Patel, A.; Kushchayeva, Y.; Costello, J.; McDaniel, D.; Burman, K.; Vasko, V. Nelfinavir inhibits proliferation and induces DNA damage in thyroid cancer cells. *Endocrine-related cancer*, **2017**, *24*, 147–156.

[62] Xiang, T.; Du, L.; Pham, P.; Zhu, B.; Jiang, S. Nelfinavir, an HIV protease inhibitor, induces apoptosis and cell cycle arrest in human cervical cancer cells via the ROS-dependent mitochondrial pathway. *Cancer Letters*, **2015**, *364*, 79–88.

[63] Blumenthal, G.M.; Gills, J.J.; Ballas, M.S.; Bernstein, W.B.; Komiya, T.; Dechowdhury, R.; Morrow, B.; Root, H.; Chun, G.; Helsabeck, C.; Steinberg, S.M.; LoPiccolo, J.; Kawabata, S.; Gardner, E.R.; Figg, W.D.; Dennis, P.A. A phase I trial of the HIV protease inhibitor nelfinavir in adults with solid tumors. *Oncotarget*, **2014**, *5*, 8161–8172.

[64] Hill, E.J.; Roberts, C.; Franklin, J.M.; Enescu, M.; West, N.; MacGregor, T.P.; Chu, K.-Y.; Boyle, L.; Blesing, C.; Wang, L.-M.; Mukherjee, S.; Anderson, E.M.; Brown, G.; Dutton, S.; Love, S.B.; Schnabel, J.A.; Quirke, P.; Muschel, R.; McKenna, W.G.; Partridge, M.; Sharma, R.A. Clinical Trial of Oral Nelfinavir before and during Radiation Therapy for Advanced Rectal Cancer. *Clinical cancer research : an official journal of the American Association for Cancer Research*, **2016**, *22*, 1922–1931.

[65] Buijsen, J.; Lammering, G.; Jansen, R.L.H.; Beets, G.L.; Wals, J.; Sosef, M.; Boer, M.O. den; Leijtens, J.; Riedl, R.G.; Theys, J.; Lambin, P. Phase I trial of the combination of the Akt inhibitor nelfinavir and chemoradiation for locally advanced rectal cancer. *Radiotherapy and oncology : journal of the European Society for Therapeutic Radiology and Oncology*, **2013**, *107*, 184–188.

[66] Rengan, R.; Mick, R.; Pryma, D.; Rosen, M.A.; Lin, L.L.; Maity, A.M.; Evans, T.L.; Stevenson, J.P.; Langer, C.J.; Kucharczuk, J.; Friedberg, J.; Prendergast, S.; Sharkoski, T.; Hahn, S.M. A phase I trial of the HIV protease inhibitor nelfinavir with concurrent chemoradiotherapy for unresectable stage IIIA/IIIB non-small cell lung cancer: a report of toxicities and clinical response. *Journal of thoracic oncology : official publication of the International Association for the Study of Lung Cancer*, **2012**, *7*, 709–715.

[67] Wilson, J.M.; Fokas, E.; Dutton, S.J.; Patel, N.; Hawkins, M.A.; Eccles, C.; Chu, K.-Y.; Durrant, L.; Abraham, A.G.; Partridge, M.; Woodward, M.; O'Neill, E.; Maughan, T.; McKenna, W.G.; Mukherjee, S.; Brunner, T.B. ARCII: A phase II trial of the HIV protease inhibitor Nelfinavir in combination with chemoradiation for locally advanced inoperable pancreatic cancer. *Radiotherapy and oncology : journal of the European Society for Therapeutic Radiology and Oncology*, **2016**, *119*, 306–311.

[68] Chow, W.A.; Guo, S.; Valdes-Albini, F. Nelfinavir induces liposarcoma apoptosis and cell cycle arrest by upregulating sterol regulatory element binding protein-1. *Anti-cancer drugs*, **2006**, *17*, 891–903.

[69] Hofmann, W.P.; Herrmann, E.; Sarrazin, C.; Zeuzem, S. Ribavirin mode of action in chronic hepatitis C: from clinical use back to molecular mechanisms. *Liver international : official journal of the International Association for the Study of the Liver*, **2008**, *28*, 1332–1343.

[70] Jin, J.; Xiang, W.; Wu, S.; Wang, M.; Xiao, M.; Deng, A. Targeting eIF4E signaling with ribavirin as a sensitizing strategy for ovarian cancer. *Biochemical and biophysical research communications*, **2019**, *510*, 580–586.

[71] Urtishak, K.A.; Wang, L.-S.; Culjkovic-Kraljacic, B.; Davenport, J.W.; Porazzi, P.; Vincent, T.L.; Teachey, D.T.; Tasian, S.K.; Moore, J.S.; Seif, A.E.; Jin, S.; Barrett, J.S.; Robinson, B.W.; Chen, I.-M.L.; Harvey, R.C.; Carroll, M.P.; Carroll, A.J.; Heerema, N.A.; Devidas, M.; Dreyer, Z.E.; Hilden, J.M.; Hunger, S.P.; Willman, C.L.; Borden, K.L.B.; Felix, C.A. Targeting EIF4E signaling with ribavirin in infant acute lymphoblastic leukemia. *Oncogene*, **2019**, *38*, 2241–2262.

[72] Pettersson, F.; Yau, C.; Dobocan, M.C.; Culjkovic-Kraljacic, B.; Retrouvey, H.; Retrouvay, H.; Puckett, R.; Flores, L.M.; Krop, I.E.; Rousseau, C.; Cocolakis, E.; Borden, K.L.B.; Benz, C.C.; Miller, W.H. Ribavirin treatment effects on breast cancers overexpressing eIF4E, a biomarker with prognostic specificity for luminal B-type breast cancer. *Clinical cancer research : an official journal of the American Association for Cancer Research*, **2011**, *17*, 2874–2884.

[73] Tan, K.; Culjkovic, B.; Amri, A.; Borden, K.L.B. Ribavirin targets eIF4E dependent Akt survival signaling. *Biochemical and biophysical research communications*, **2008**, *375*, 341–345.

[74] Huq, S.; Casaos, J.; Serra, R.; Peters, M.; Xia, Y.; Ding, A.S.; Ehresman, J.; Kedda, J.N.; Morales, M.; Gorelick, N.L.; Zhao, T.; Ishida, W.; Perdomo-Pantoja, A.; Cecia, A.; Ji, C.; Suk, I.; Sidransky, D.; Brait, M.; Brem, H.; Skuli, N.; Tyler, B. Repurposing the FDA-Approved Antiviral Drug Ribavirin as Targeted Therapy for Nasopharyngeal Carcinoma. *Molecular cancer therapeutics*, **2020**, *19*, 1797–1808.

[75] Ogino, A.; Sano, E.; Ochiai, Y.; Yamamuro, S.; Tashiro, S.; Yachi, K.; Ohta, T.; Fukushima, T.; Okamoto, Y.; Tsumoto, K.; Ueda, T.; Yoshino, A.; Katayama, Y. Efficacy of ribavirin against malignant glioma cell lines. *Oncology letters*, **2014**, *8*, 2469–2474.

[76] Ochiai, Y.; Sano, E.; Okamoto, Y.; Yoshimura, S.; Makita, K.; Yamamuro, S.; Ohta, T.; Ogino, A.; Tadakuma, H.; Ueda, T.; Nakayama, T.; Hara, H.; Yoshino, A.; Katayama, Y. Efficacy of ribavirin against malignant glioma cell lines: Follow-up study. *Oncology reports*, **2018**, *39*, 537–544.

[77] Chen, J.; Xu, X.; Chen, J. Clinically relevant concentration of anti-viral drug ribavirin selectively targets pediatric osteosarcoma and increases chemosensitivity. *Biochemical and biophysical research communications*, **2018**, *506*, 604–610.

[78] Volpin, F.; Casaos, J.; Sesen, J.; Mangraviti, A.; Choi, J.; Gorelick, N.; Frikeche, J.; Lott, T.; Felder, R.; Scotland, S.J.; Eisinger-Mathason, T.S.K.; Brem, H.; Tyler, B.; Skuli, N. Use of an anti-viral drug, Ribavirin, as an anti-glioblastoma therapeutic. *Oncogene*, **2017**, *36*, 3037–3047.

[79] Teng, L.; Ding, D.; Chen, Y.; Dai, H.; Liu, G.; Qiao, Z.; An, R. Anti-tumor effect of ribavirin in combination with interferon-α on renal cell carcinoma cell lines in vitro. *Cancer cell international*, **2014**, *14*, 63.

[80] Kraljacic, B.C.; Arguello, M.; Amri, A.; Cormack, G.; Borden, K. Inhibition of eIF4E with ribavirin cooperates with common chemotherapies in primary acute myeloid leukemia specimens. *Leukemia*, **2011**, *25*, 1197–1200.

[81] Tan, J.; Ye, J.; Song, M.; Zhou, M.; Hu, Y. Ribavirin augments doxorubicin's efficacy in human hepatocellular carcinoma through inhibiting doxorubicin-induced eIF4E activation. *Journal of biochemical and molecular toxicology*, **2018**, *32*.

[82] Shi, F.; Len, Y.; Gong, Y.; Shi, R.; Yang, X.; Naren, D.; Yan, T. Ribavirin Inhibits the Activity of mTOR/eIF4E, ERK/Mnk1/eIF4E Signaling Pathway and Synergizes with Tyrosine Kinase Inhibitor Imatinib to Impair Bcr-Abl Mediated Proliferation and Apoptosis in Ph+ Leukemia. *PloS one*, **2015**, *10*, e0136746.

[83] Dai, D.; Chen, H.; Tang, J.; Tang, Y. Inhibition of mTOR/eIF4E by anti-viral drug ribavirin effectively enhances the effects of paclitaxel in oral tongue squamous cell carcinoma. *Biochemical and biophysical research communications*, **2017**, *482*, 1259–1264.

[84] Casaos, J.; Gorelick, N.L.; Huq, S.; Choi, J.; Xia, Y.; Serra, R.; Felder, R.; Lott, T.; Kast, R.E.; Suk, I.; Brem, H.; Tyler, B.; Skuli, N. The Use of Ribavirin as an Anticancer Therapeutic: Will It Go Viral? *Molecular cancer therapeutics*, **2019**, *18*, 1185–1194.

[85] Kosaka, T.; Maeda, T.; Shinojima, T.; Nagata, H.; Mizuno, R.; Oya, M. A clinical study to evaluate the efficacy and safety of docetaxel with ribavirin in patients with progressive castration resistant prostate cancer who have previously received docetaxel alone. *JCO*, **2017**, *35*, e14010-e14010.

[86] Assouline, S.; Culjkovic, B.; Cocolakis, E.; Rousseau, C.; Beslu, N.; Amri, A.; Caplan, S.; Leber, B.; Roy, D.-C.; Miller, W.H.; Borden, K.L.B. Molecular targeting of the oncogene eIF4E in acute myeloid leukemia (AML): a proof-of-principle clinical trial with ribavirin. *Blood*, **2009**, *114*, 257–260.

[87] Nunes, M.; Henriques Abreu, M.; Bartosch, C.; Ricardo, S. Recycling the Purpose of Old Drugs to Treat Ovarian Cancer. *International journal of molecular sciences*, **2020**, *21*.

[88] Dewan, M.Z.; Uchihara, J.; Terashima, K.; Honda, M.; Sata, T.; Ito, M.; Fujii, N.; Uozumi, K.; Tsukasaki, K.; Tomonaga, M.; Kubuki, Y.; Okayama, A.; Toi, M.; Mori, N.; Yamamoto, N. Efficient intervention of growth and infiltration of primary adult T-cell leukemia cells by an HIV protease inhibitor, ritonavir. *Blood*, **2006**, *107*, 716–724.

[89] Gaedicke, S.; Firat-Geier, E.; Constantiniu, O.; Lucchiari-Hartz, M.; Freudenberg, M.; Galanos, C.; Niedermann, G. Antitumor effect of the human immunodeficiency virus protease inhibitor ritonavir: induction of tumor-cell apoptosis associated with perturbation of proteasomal proteolysis. *Cancer research*, **2002**, *62*, 6901–6908.

[90] Ikezoe, T.; Daar, E.S.; Hisatake, J.; Taguchi, H.; Koeffler, H.P. HIV-1 protease inhibitors decrease proliferation and induce differentiation of human myelocytic leukemia cells. *Blood*, **2000**, *96*, 3553–3559.

[91] Srirangam, A.; Mitra, R.; Wang, M.; Gorski, J.C.; Badve, S.; Baldridge, L.; Hamilton, J.; Kishimoto, H.; Hawes, J.; Li, L.; Orschell, C.M.; Srour, E.F.; Blum, J.S.; Donner, D.; Sledge, G.W.; Nakshatri, H.; Potter, D.A. Effects of HIV protease inhibitor ritonavir on Akt-regulated cell proliferation in breast cancer. *Clinical cancer research : an official journal of the American Association for Cancer Research*, **2006**, *12*, 1883–1896.

[92] Kumar, S.; Bryant, C.S.; Chamala, S.; Qazi, A.; Seward, S.; Pal, J.; Steffes, C.P.; Weaver, D.W.; Morris, R.; Malone, J.M.; Shammas, M.A.; Prasad, M.; Batchu, R.B. Ritonavir blocks AKT signaling, activates apoptosis and inhibits migration and invasion in ovarian cancer cells. *Molecular cancer*, **2009**, *8*, 26.

[93] Batchu, R.B.; Gruzdyn, O.V.; Bryant, C.S.; Qazi, A.M.; Kumar, S.; Chamala, S.; Kung, S.T.; Sanka, R.S.; Puttagunta, U.S.; Weaver, D.W.; Gruber, S.A. Ritonavir-Mediated Induction of Apoptosis in Pancreatic Cancer Occurs via the RB/E2F-1 and AKT Pathways. *Pharmaceuticals (Basel, Switzerland)*, **2014**, *7*, 46–57.

[94] Srirangam, A.; Milani, M.; Mitra, R.; Guo, Z.; Rodriguez, M.; Kathuria, H.; Fukuda, S.; Rizzardi, A.; Schmechel, S.; Skalnik, D.G.; Pelus, L.M.; Potter, D.A. The human immunodeficiency virus protease inhibitor ritonavir inhibits lung cancer cells, in part, by inhibition of survivin. *Journal of thoracic oncology : official publication of the International Association for the Study of Lung Cancer*, **2011**, *6*, 661–670.

[95] Pati, S.; Pelser, C.B.; Dufraine, J.; Bryant, J.L.; Reitz, M.S.; Weichold, F.F. Antitumorigenic effects of HIV protease inhibitor ritonavir: inhibition of Kaposi sarcoma. *Blood*, **2002**, *99*, 3771–3779.

[96] Dalva-Aydemir, S.; Bajpai, R.; Martinez, M.; Adekola, K.U.A.; Kandela, I.; Wei, C.; Singhal, S.; Koblinski, J.E.; Raje, N.S.; Rosen, S.T.; Shanmugam, M. Targeting the metabolic plasticity of multiple myeloma with FDA-approved ritonavir and metformin. *Clinical cancer research : an official journal of the American Association for Cancer Research*, **2015**, *21*, 1161–1171.
[97] Laurent, N.; Boüard, S. de; Guillamo, J.-S.; Christov, C.; Zini, R.; Jouault, H.; Andre, P.; Lotteau, V.; Peschanski, M. Effects of the proteasome inhibitor ritonavir on glioma growth in vitro and in vivo. *Molecular cancer therapeutics*, **2004**, *3*, 129–136.
[98] Ikezoe, T.; Hisatake, Y.; Takeuchi, T.; Ohtsuki, Y.; Yang, Y.; Said, J.W.; Taguchi, H.; Koeffler, H.P. HIV-1 protease inhibitor, ritonavir: a potent inhibitor of CYP3A4, enhanced the anticancer effects of docetaxel in androgen-independent prostate cancer cells in vitro and in vivo. *Cancer research*, **2004**, *64*, 7426–7431.
[99] Wang, H.; Zhou, J.; He, Q.; Dong, Y.; Liu, Y. Azidothymidine inhibits cell growth and telomerase activity and induces DNA damage in human esophageal cancer. *Molecular medicine reports*, **2017**, *15*, 4055–4060.
[100] Langtry, H.D.; Campoli-Richards, D.M. Zidovudine. A review of its pharmacodynamic and pharmacokinetic properties, and therapeutic efficacy. *Drugs*, **1989**, *37*, 408–450.
[101] Li, H.; Song, T.; Xu, W.; Yu, Y.; Xin, X.; Du Hui. Effect of 3`-Azido-3`-Deoxythymidine (AZT) on Telomerase Activity and Proliferation of HO-8910 Cell Line of Ovarian Cancer. *International Journal of Biomedical Science : IJBS*, **2006**, *2*, 34–40.
[102] Brown, T.; Sigurdson, E.; Rogatko, A.; Broccoli, D. Telomerase inhibition using azidothymidine in the HT-29 colon cancer cell line. *Annals of surgical oncology*, **2003**, *10*, 910–915.
[103] Fang, J.-L.; Beland, F.A. Long-term exposure to zidovudine delays cell cycle progression, induces apoptosis, and decreases telomerase activity in human hepatocytes. *Toxicological sciences : an official journal of the Society of Toxicology*, **2009**, *111*, 120–130.
[104] Falchetti, A.; Franchi, A.; Bordi, C.; Mavilia, C.; Masi, L.; Cioppi, F.; Recenti, R.; Picariello, L.; Marini, F.; Del Monte, F.; Ghinoi, V.; Martineti, V.; Tanini, A.; Brandi, M.L. Azidothymidine induces apoptosis and inhibits cell growth and telomerase activity of human parathyroid cancer cells in culture. *Journal of bone and mineral research : the official journal of the American Society for Bone and Mineral Research*, **2005**, *20*, 410–418.
[105] Mediavilla, M.D.; Sánchez-Barceló, E.J. Doses and time-dependent effects of 3'-azido-3'-deoxythymidine on T47D human breast cancer cells in vitro. *Pharmacology & toxicology*, **2000**, *87*, 138–143.
[106] Mattson, D.M.; Ahmad, I.M.; Dayal, D.; Parsons, A.D.; Aykin-Burns, N.; Li, L.; Orcutt, K.P.; Spitz, D.R.; Dornfeld, K.J.; Simons, A.L. Cisplatin combined with zidovudine enhances cytotoxicity and oxidative stress in human head and neck cancer cells via a thiol-dependent mechanism. *Free radical biology & medicine*, **2009**, *46*, 232–237.
[107] Johnston, J.S.; Johnson, A.; Gan, Y.; Wientjes, M.G.; Au, J.L.S. Synergy between 3'-azido-3'-deoxythymidine and paclitaxel in human pharynx FaDu cells. *Pharmaceutical research*, **2003**, *20*, 957–961.
[108] Ji, H.J.; Rha, S.Y.; Jeung, H.C.; Yang, S.H.; An, S.W.; Chung, H.C. Cyclic induction of senescence with intermittent AZT treatment accelerates both apoptosis and telomere loss. *Breast cancer research and treatment*, **2005**, *93*, 227–236.
[109] Wagner, C.R.; Ballato, G.; Akanni, A.O.; McIntee, E.J.; Larson, R.S.; Chang, S.; Abul-Hajj, Y.J. Potent growth inhibitory activity of zidovudine on cultured human breast cancer cells and rat mammary tumors. *Cancer research*, **1997**, *57*, 2341–2345.
[110] Yasuda, C.; Kato, M.; Kuroda, D.; Ohyanagi, H. Experimental studies on potentiation of the antitumor activity of 5-fluorouracil with 3'-azido-3'-deoxythymidine for the gastric cancer cell line MKN28 in vivo. *Japanese journal of cancer research : Gann*, **1997**, *88*, 97–102.
[111] Sun, Y.-Q.; Guo, T.-K.; Xi, Y.-M.; Chen, C.; Wang, J.; Wang, Z.-R. Effects of AZT and RNA-protein complex (FA-2-b-beta) extracted from Liang Jin mushroom on apoptosis of gastric cancer cells. *World journal of gastroenterology*, **2007**, *13*, 4185–4191.
[112] Zhou, F.-X.; Liao, Z.-K.; Dai, J.; Xiong, J.; Xie, C.-H.; Luo, Z.-G.; Liu, S.-Q.; Zhou, Y.-F. Radiosensitization effect of zidovudine on human malignant glioma cells. *Biochemical and biophysical research communications*, **2007**, *354*, 351–356.
[113] Tosi, P.; Gherlinzoni, F.; Visani, G.; Coronado, O.; Costigliola, P.; Mazzetti, M.; Gritti, F.; Chiodo, F. AZT plus methotrexate in HIV-related non-Hodgkin's lymphomas. *Leukemia & lymphoma*, **1998**, *30*, 175–179.
