# Supplementary material for: Repurposing of Antimicrobial Agents for Cancer Therapy: What Do We Know?
Source: Cancers (Basel). 2021 Jun 26;13(13):3193. doi: 10.3390/cancers13133193 (PMC8269327; doi:10.3390/cancers13133193)
Supplement: Supplementary file 1 [file cancers-13-03193-s001.zip › Supplemental Data Table S3.pdf]

Table S3. List of antifungal agents that are currently being investigated in clinical trials for their effectiveness against different cancer types when alone and/or in combination with chemotherapeutic agents as well as antifungals that are potential new anti-cancer drugs shown to inhibit malignant cells *in vivo*/*in vitro*. The summarized data is obtained from clinicaltrials.gov, the National Institute of Health (NIH), the Drugbank, and the ReDo databank. PubMed and Google Scholar have been used to gain detailed information about the mechanism(s) of action of the potential repurposed drugs *in vitro* and *in vivo*. The keywords used for the search are "anti-cancer" and "repurposed".

Drug	Original application / target	New application (anti-cancer) / Proposed Target/Mechanism of action	Stage of development
**Clioquinol**	**Antiseptic**<br>High affinity to disulfhydrile groups → inhibition of essential enzymes [1, 2]	**Prostate cancer:**<br>-As single treatment:<br>Acts as zinc ionophore → disrupts lysosome integrity, cleaves Bid → induces apoptosis [3] and inhibits NF-κB activity [4], inhibits cell viability and increases caspase activity [5]	**Clinical trial Phase I:**<br>Leukemia (NCT00963495)<br><br>***In vitro:***<br>-Lymphoma cells Raji and ovarian cancer cells A1780 in nude mice [5]<br>***In vitro:***<br>-Prostate cancer cells DU 145 [3, 4]<br>-B-cell lymphoma lines DHL-4, Raji, breast MCF-7, MDA-MB231, ovarian A2780, cervical SiHa, bladder T24, and pancreatic Mpanc-96 cancer cells [5]
**Ketoconazol**	**Antifungal**<br>Inhibition of ergosterol synthesis [6]	**Prostate cancer:**<br>-As single treatment:<br>Blocks the production of androgen, necessary to enhance prostate tumor growth [7, 8]	**Clinical trials Phase II:**<br>-Prostate cancer (NCT00895310)<br>-Metastatic breast cancer (NCT00212095)<br>-Prostate cancer (NCT01036594)<br>-Prostatic neoplasms (NCT00447473)<br>-Prostate cancer in a drug combination with hydrocortisone (NCT00673127)
**Terbinafine**	**Antifungal**<br>Inhibition of ergosterol synthesis by inhibition of squalene monooxygenase [16, 17]	**Melanoma:**<br>-In combination:<br>Enhances radiation' effect (radiosensitizer) [18]	***In vivo:***<br>-Murine melanoma B16.SIY cells in C57BL/6 female mice [18]

**Itraconazol**	**Antifungal**<br>Inhibition of ergosterol synthesis, disruption of cell wall synthesis [9, 10]	**Pancreatic, ovarian cancer:**<br>- In combination with Docetaxel, Gemcitabine, and Carboplatin:<br>Enhances over-all survival of patients [11, 12],<br>Inhibits angiogenesis and Hedgehog (Hh) signaling (due to triazole unit in the molecule) [11, 13]<br>**Melanoma:**<br>-As single treatment:<br>Inhibits tumor cell growth, cell proliferation, and colony formation<br>Inhibits Hedgehog and Wnt signaling by downregulation of Gli-1, Gli-2, Wnt3A, β-catenin and cyclin D1<br>Suppresses PI3K/mTOR signaling pathway by downregulation of the phosphorylation of p70S6K, 4E-BP1 and AKT [14]<br>Inhibits cell progression, induces G1 arrest, inhibits angiogenesis by blocking the vascular endothelial growth factor (*in vivo* and *in-vitro*) [15]	**Clinical trial Phase II:**<br>-Prostate cancer (NCT00887458)<br>-Lung cancer (NCT03664115)<br>-Neoplasm (NCT02366884)<br><br>**Clinical trial Phase I:**<br>-Esophageal cancer (NCT02749513)<br>-Lung cancer (NCT02357836)<br><br>***In vitro:***<br>-Xenograft model with melanoma A735 cells in mice [14]<br><br>***In vitro:***<br>-Melanoma cells SK-MEL-28 and A375 [14]
**Clotrimazol**	**Antifungal**<br>Inhibition of ergosterol synthesis – inhibition of the fungus cell growth and cell wall permeability [19]	**Breast cancer:**<br>**-**As single treatment:[15]<br>Induces morphological changes, inhibits lactate production and glucose consumption → inhibits glycolytic enzymes such as glycolytic enzyme 6-phosphofructo-1-kinase by dissociation of f-actin [20–22]<br>**Colon and lung cancer:**<br>**-**As single treatment:<br>Detaches phosphofructokinase and aldolase from cytoskeleton [23]<br>**Melanoma:**<br>**-**As single treatment:<br>Detaches hexokinase from mitochondria [24]<br>Inhibits Ca^{2+}-activated potassium channel [25]<br>-In combination:<br>Enhances radiation' effect (radiosensitizer) [18]	***In vivo:***<br>-Murine melanoma B16.SIY cells in C57BL/6 female mice [18]<br><br>***In vitro:***<br>-Human breast cancer tissue [20]<br>-Breast cancer cells MCF10A, MCF-7 and MDA-MB-231 [21, 22]<br>-Colon cancer cells CT-26 and LL/2 Lewis lung carcinoma cells [23]<br>-B16 Melanoma cells [24]

References

[1] Bareggi, S.R.; Cornelli, U. Clioquinol: review of its mechanisms of action and clinical uses in neurodegenerative disorders. *CNS neuroscience & therapeutics*, **2012**, *18*, 41–46.

[2] https://www.gelbe-liste.de/wirkstoffe/Clioquinol_1890. Clioquinol, **March 23, 2021**.

[3] Yu, H.; Zhou, Y.; Lind, S.E.; Ding, W.-Q. Clioquinol targets zinc to lysosomes in human cancer cells. *The Biochemical journal*, **2009**, *417*, 133–139.

[4] Yu, H.; Lou, J.R.; Ding, W.-Q. Clioquinol independently targets NF-kappaB and lysosome pathways in human cancer cells. *Anticancer research*, **2010**, *30*, 2087–2092.

[5] Ding, W.-Q.; Liu, B.; Vaught, J.L.; Yamauchi, H.; Lind, S.E. Anticancer activity of the antibiotic clioquinol. *Cancer research*, **2005**, *65*, 3389–3395.

[6] Borgers, M.; van den Bossche, H.; Brabander, M. de. The mechanism of action of the new antimycotic ketoconazole. *The American Journal of Medicine*, **1983**, *74*, 2–8.

[7] Bok, R.A.; Small, E.J. The treatment of advanced prostate cancer with ketoconazole: safety issues. *Drug safety*, **1999**, *20*, 451–458.

[8] Patel, V.; Liaw, B.; Oh, W. The role of ketoconazole in current prostate cancer care. *Nature reviews. Urology*, **2018**, *15*, 643–651.

[9] Leyden, J. Pharmacokinetics and pharmacology of terbinafine and itraconazole. *Journal of the American Academy of Dermatology*, **1998**, *38*, S42-S47.

[10] https://www.gelbe-liste.de/wirkstoffe/Itraconazol_10331. Itroconazol, **March 25, 2021**.

[11] Tsubamoto, H.; Sonoda, T.; Ikuta, S.; Tani, S.; Inoue, K.; Yamanaka, N. Combination Chemotherapy with Itraconazole for Treating Metastatic Pancreatic Cancer in the Second-line or Additional Setting. *Anticancer research*, **2015**, *35*, 4191–4196.

[12] Tsubamoto, H.; Ueda, T.; Inoue, K.; Sakata, K.; Shibahara, H.; Sonoda, T. Repurposing itraconazole as an anticancer agent. *Oncology letters*, **2017**, *14*, 1240–1246.

[13] Pace, J.R.; DeBerardinis, A.M.; Sail, V.; Tacheva-Grigorova, S.K.; Chan, K.A.; Tran, R.; Raccuia, D.S.; Wechsler-Reya, R.J.; Hadden, M.K. Repurposing the Clinically Efficacious Antifungal Agent Itraconazole as an Anticancer Chemotherapeutic. *Journal of medicinal chemistry*, **2016**, *59*, 3635–3649.

[14] Liang, G.; Liu, M.; Wang, Q.; Shen, Y.; Mei, H.; Li, D.; Liu, W. Itraconazole exerts its anti-melanoma effect by suppressing Hedgehog, Wnt, and PI3K/mTOR signaling pathways. *Oncotarget*, **2017**, *8*, 28510–28525.

[15] Chong, C.R.; Xu, J.; Lu, J.; Bhat, S.; Sullivan, D.J.; Liu, J.O. Inhibition of angiogenesis by the antifungal drug itraconazole. *ACS chemical biology*, **2007**, *2*, 263–270.

[16] Darkes, M.J.M.; Scott, L.J.; Goa, K.L. Terbinafine: a review of its use in onychomycosis in adults. *American journal of clinical dermatology*, **2003**, *4*, 39–65.

[17] Ryder, N.S. Terbinafine: mode of action and properties of the squalene epoxidase inhibition. *The British journal of dermatology*, **1992**, *126 Suppl 39*, 2–7.

[18] Labay, E.; Mauceri, H.J.; Efimova, E.V.; Flor, A.C.; Sutton, H.G.; Kron, S.J.; Weichselbaum, R.R. Repurposing cephalosporin antibiotics as pro-senescent radiosensitizers. *Oncotarget*, **2016**, *7*, 33919–33933.

[19] Crowley, P.D.; Gallagher, H.C. Clotrimazole as a pharmaceutical: past, present and future. *Journal of applied microbiology*, **2014**, *117*, 611–617.

[20] Coelho, R.G.; Calaça, I.d.C.; Celestrini, D.d.M.; Correia, A.H.; Costa, M.A.S.M.; Sola-Penna, M. Clotrimazole disrupts glycolysis in human breast cancer without affecting non-tumoral tissues. *Molecular genetics and metabolism*, **2011**, *103*, 394–398.

[21] Furtado, C.M.; Marcondes, M.C.; Sola-Penna, M.; Souza, M.L.S. de; Zancan, P. Clotrimazole preferentially inhibits human breast cancer cell proliferation, viability and glycolysis. *PloS one*, **2012**, *7*, e30462.

[22] Meira, D.D.; Marinho-Carvalho, M.M.; Teixeira, C.A.; Veiga, V.F.; Da Poian, A.T.; Holandino, C.; Freitas, M.S. de; Sola-Penna, M. Clotrimazole decreases human breast cancer cells viability through alterations in cytoskeleton-associated glycolytic enzymes. *Molecular genetics and metabolism*, **2005**, *84*, 354–362.

[23] Penso, J.; Beitner, R. Clotrimazole decreases glycolysis and the viability of lung carcinoma and colon adenocarcinoma cells. *European journal of pharmacology*, **2002**, *451*, 227–235.

[24] Penso, J.; Beitner, R. Clotrimazole and bifonazole detach hexokinase from mitochondria of melanoma cells. *European journal of pharmacology*, **1998**, *342*, 113–117.

[25] Kadavakollu, S.; Stailey, C.; Kunapareddy, C.S.; White, S. Clotrimazole as a Cancer Drug: A Short Review. *Medicinal chemistry*, **2014**, *4*, 722–724.
