# Supplementary material for: Repurposing of Antimicrobial Agents for Cancer Therapy: What Do We Know?
Source: Cancers (Basel). 2021 Jun 26;13(13):3193. doi: 10.3390/cancers13133193 (PMC8269327; doi:10.3390/cancers13133193)
Supplement: Supplementary file 1 [file cancers-13-03193-s001.zip › Supplemental Data Table S4.pdf]

Table S4. List of anti-malarial agents that are currently being investigated in clinical trials for their effectiveness against different cancer types when alone and/or in combination with chemotherapeutic agents as well as anti-malarials that are potential new anti-cancer drugs shown to inhibit malignant cells in *vivo*/*in vitro*. The summarized data is obtained from clinicaltrials.gov, the National Institute of Health (NIH), the Drugbank, and the ReDo databank. PubMed and Google Scholar have been used to gain detailed information about the mechanism(s) of action of the potential repurposed drugs *in vitro* and *in vivo*. The keywords used for the search are "anti-cancer" and "repurposed".

**Drug**	**Original application / target**	**New application (anti-cancer) / Proposed Target/Mechanism of action**	**Stage of development**
**Artesunate**	**Anti-Malaria**<br>Cleavage of endoperoxide bridge → ROS generation in parasite → DNA doublestrand breaks [1]	**Hepatocellular carcinoma (HCC) and Head and neck cancer (HNC):**<br>-As single treatment<br>Induces iron-dependent, ROS-mediated ferroptosis [2, 3]<br>**HCC and Renal cell carcinoma (RCC):**<br>-In combination<br>Synergizes with Sorafenib [2, 4, 5]<br>**Breast cancer:**<br>**-**In combination<br>Synergizes with DOX and PTX [6]<br>Upregulates Beclin1 → autophagy → G2/M cell cycle arrest and chemo sensitization to Epirubicin [7]<br>Induces ROS-dependent G2/M cell cycle arrest and apoptosis, leads to chemo sensitization to different chemotherapeutics [8]<br>-As single treatment<br>Disrupts endolysosomal trafficking and inhibits autophagic flux [9]<br>**Glioblastoma:**<br>-In combination<br>(Supra-)additive effects with OSI-774/ sensitivity and resistance dependent on genetic alterations [10]<br>Downregulates survivin → induces apoptosis and G2/M phase arrest, reduces DDR in combination with X-irradiation [11]<br>-As single treatment<br>Induces oxidative DNA stress → induces DSB and DDR → necrosis and apoptosis [12]	**Clinical trial Phase I:**<br>-Metastatic breast cancer (NCT00764036)<br>-Solid tumors (NCT02353026)<br>-Cervical neoplasia intravaginally (NCT02354534)<br>-Vulvar neoplasia: ointment (NCT03792516)<br>-Anal neoplasia: intra-anally (NCT03100045)<br>-HCC (NCT02304289)<br><br>**Clinical trial Phase II:**<br>-Pre-operative in stage II/III Colon (NCT02633098, NCT03093129)<br>-Cervical neoplasia: intravaginally (NCT04098744)

**Artesunate**<br>*(Continued)*		**Gastric cancer:**<br>-As single treatment<br>Leads to release of cytochrome c and activation of Caspase-9 via ROS production → apoptosis via COX-2 inhibition [13]<br>Leads to oncosis-like cell death via increased cytosolic Ca2+ → calpain-2 activation and VEGF inhibition [14]<br>**T-cell leukemia:**<br>-In combination<br>Synergizes with DOX [15]<br>Leads to the release of cytochrome c and activation of Caspase-9 via ROS production → apoptosis via intrinsic pathway [15]<br>**Lung cancer:**<br>-As single treatment<br>Increases radiosensitivity via increased NO production → G2/M phase arrest via reduced Cyclin B1 mRNA expression [16]<br>Cytotoxicity is dependent on the expression of glutathione-related Enzymes [17]<br>**T-cell leukemia:**<br>-In combination<br>Synergizes with DOX [15]<br>Leads to the release of cytochrome c and activation of Caspase-9 via ROS production → apoptosis via intrinsic pathway [15]<br>**Karposi's sarcoma:**<br>-As single treatment<br>Inhibits angiogenesis and tumor [18]<br>**HCC:**<br>-As single treatment<br>Reduces VEGF and PlGF expression → decreases angiogenesis [4]<br>**Lung cancer:**<br>-As single treatment<br>Increases radiosensitivity via increased NO production → G2/M phase arrest via reduced Cyclin B1 mRNA expression [16]<br>Cytotoxicity is dependent on the expression of glutathione-related Enzymes [17]	***In vivo:***<br>-HNC cells HN9 in athymic nude BALB/c nude mice [3]<br>-Liver cancer cells Huh7 in nude Balb/c mice [2]<br>DEN-induced HCC model in 129S2/SvPasCrl mice [4]<br>-Lung cancer cells A549 in nude mice [16]<br>-Kaposi's sarcoma cells KS-IMM in C57BL/6 mice and nude (CD-1) BR mice [18]<br>-RCC cells 786-O-Luc in nude BALB/c mice [5]<br>-NPC cells C666-1 or CNE2 in SCID mice [19]<br>-Gastric cancer cells BGC-823 cells in nude athymic BALB/c mice [14]<br>-Pancreatic cancer cells Panc-1 in nude athymic BALB/c mice [14]

**Artesunate**<br>*(Continued)*		**RCC:**<br>-As single treatment<br>Reduces cyclin D1 levels → G2/M cell cycle arrest [5]<br>Increases calpain-1 and calpain-2 expression → oncosis-like cell death [5]<br>Inhibits cell proliferation, migration/metastasis and angiogenesis via decreased phosphorylation of VEGFR2 [5]<br>**Pancreatic cancer:**<br>-As single treatment<br>Induces Oncosis, loss of ΔΨm via mitochondrial dysfunction and ROS production [20]<br>**Cervical and breast cancer:**<br>-As single treatment<br>Activates lysosomal function → increases lysosomal degradation of ferritin → necessary for ROS production and apoptosis [9, 21]<br>**Ovarian cancer:**<br>Sensitivity dependent on TGF/Wnt pathway genes [22]<br>**Nasopharyngeal Carcinoma (NPC):**<br>-As single treatment<br>Upregulates Cyclin B1 and downregulates Rb and E2F-1 expression → G2/M cell cycle arrest [19]<br>Induces mitochondrial-independent apoptosis, apoptosis via Akt/mTOR inhibition and ROS production [19]<br>-In combination<br>Synergizes with Cisplatin [19]<br>**Non-Hodgkin lymphoma (NHL):**<br>-As single treatment<br>Increases Beclin-1 and LC3-I/II expression, increases Caspase-3 expression → autophagy and apoptosis simultaneously [23]<br>**Cervical cells:**<br>-In combination<br>Enhances TRAIL cytotoxicity via decreased survivin, XIAP and Bcl-XL expression [24]	***In vitro:***<br>-HNC cells SNU, HN2-10, HN3-cisR, HN4-cisR, and HN9-cisR [3]<br>-Liver cancer cells Huh7, HepG2 [4, 21], SNU-182 and SNU-449 [2], BWTG3 [4]<br>-Breast cancer cells MCF-7 [6–8], MDA-MB-231 [7, 8] and T47D [9], SK-BR-3 [8]<br>-Glioblastoma cells LN-229 [11, 12], G-211GM, G-750GM, G-1163GM, G-1187GM, G-1265GM, G-1301GM, G-1408GM, G-210GM, G-599GM, U-87MG.LUX [29], U-87MG.DK-2N, U-87MG.WT-2N, U-87MG [11] and U-87MG.ΔEGFR [10, 29]<br>-T-cell Leukemia cells Hut78, CEM, Molt-4, Jurkat J16, J-Neo, J-Bcl-2, J-caspase-8$^{-/-}$, Jurkat A3 FADD$^{-/-}$ and Jurkat A3 [15], CCRFCEM and HL-60 [29]<br>-Lung cancer cells A549 [16]<br>-Kaposi's sarcoma cells Kaposi's sarcoma-IMM [18]<br>-RCC cells Caki-1-GFP, 786-O-GFP and SN12C-GFP [5], HCT116 [29]<br>-NPC cells C666-1, HONE-1, HK1, HNE1, CNE2 [19]<br>-Gastric cancer cells BGC-823, HGC-27 and MGC-803 [13], SGC-7901, BGC-823, and AGS [14]<br>-Pancreatic cancer cells Panc-1, BxPC-3 and CFPAC-1 [20]<br>-Stem cells TSC2-WT and TSC2-KO MEFs [21]<br>-Cervical cancer cells HeLa [21, 24]<br>-7 different ovarian cancer cell lines [22]<br>-NHL cells Raji [23]<br>-MSV-HL13 and MSV-PC4 cells, panel of 55 different cell lines [17, 29–31], WEHI7.2 cells [31]

**Artesunate** *(Continued)*		More extensive summary is presented in the reviews of Augustin et al., Li et al., Xu et al., and Sun et al. [25–28]	
**Mefloquine**	**Anti-Malaria** no known mode of action, but Wong et al. showed that it target the 80S-ribosome of the parasite [32]	**Breast cancer:**<br>-In combination<br>Synergizes with Doxorubicin and PAX [6]<br>Chemo sensitizes to PAX [33]<br>-As single treatment<br>Enhanced cleavage of caspase 7 and PARP → apoptosis induction [33]<br>-Arrests autophagy at the stage of autophagosome formation via increased LC3 and p62 expression [33]<br>**Osteosarcoma (OSC):**<br>-In combination<br>Sensitizes resistant cells to vinblastine via inhibition of P-gp and increased apoptosis [34]<br>**Leukemia:**<br>-In combination<br>Sensitizes resistant cells to Doxorubicin via decreased P-gp expression and inhibited pump-efflux activity of the same [35]<br>**Prostate cancer:**<br>-As single treatment<br>Increases ROS generation → induces apoptosis and necrosis [36]<br>Induces G_1 cell cycle arrest via increased cyclin D1 protein expression [36]<br>Exerts anti-clonogenic effects [37]<br>Hyperpolarizes MMP → ROS production → decreases Akt Ser473 phosphorylation, activates ERK, JNK and AMPK signaling → decreases cell viability [37]<br>**Gastric cancer:**<br>-As single treatment<br>Inhibits PI3K/Akt/mTOR phosphorylation and signaling [38]<br>-In combination<br>Synergizes with PAX [38]	**Clinical trial Phase I:**<br>-Glioblastoma: in combination with Temozolomide, Memantine Hydrochloride, and Metformin Hydrochloride after surgery (NCT01430351)<br>Results [39]<br><br>***In vivo:***<br>-Prostate cancer cells PC3 in C57BL/6J mice [36]<br>-Gastric cancer cells YCC1 or SNU1 in SCID mice [38]<br>-Cervical cancer cells HeLa in SCID mice [40]<br>-Liver cancer cells HepG2 in nude BALB/c mice [41]<br>-Colon cancer cells HCT116 in nude mice [42]<br>-AML cells MDAY-D2, K562 or OCI-AML2 in NOD/SCID mice [43]

**Mefloquine**<br>*(Continued)*		**Stem cells:**<br>-As single treatment<br>Reduces the expression levels of β-catenin and cyclin D1 → decreases self-renewal and proliferation [41]<br>**Cervical cancer:**<br>-As single treatment<br>-Leads to mitochondrial dysfunction → ROS production and energy crisis, suppression of mTOR phosphorylation → inhibits proliferation and induces apoptosis [40]<br>-In combination<br>Synergizes with PAX [40]<br>**Colon cancer:**<br>-As single treatment<br>Inhibits IKK and NF-κB activation → induces apoptosis [42]<br>-In combination<br>Synergizes with Doxorubicin [42]<br>**Acute myeloid leukemia (AML):**<br>-As single treatment<br>Disrupts lysosomes via ROS production → permeabilizes lysosome membrane → release of cathepsins → decreases proliferation and increased death [43]<br>**Neuroblastoma:**<br>-As single treatment<br>Induces autophagy in an ATG6 dependent manner via formation of autophagosomes and the conversion of LC3I into LC3II → suppression of autophagy increases Mefloquine cytotoxicity [44]	***In vitro:***<br>-Breast cancer cells MCF-7 [6], MDA-MB-231, MDA-MB-468 and T47D [33]<br>-OSC cells KB and KBV20C [34]<br>-Prostate cancer cells PC3 [36], DU145 [37]<br>-Leukemia cells K562 and K562/DXR [35]<br>-Gastric cancer cells SNU1, SNU16, AGS, Hs746T, NCI-N87, MKN45, MKN74, YCC1, YCC10 and YCC11 [38]<br>-Cervical cancer cells HeLa, SiHa and C-33A [40]<br>-Liver cancer cells HepG2 and CD133+ HepG2 [41]<br>-Colon cancer cells HT-29, HCT116, RKO, SW620 and Lovo [42]<br>-Kidney cancer cells HEK293T [42]<br>-Primary AML patient cells [43]<br>-Neuroblastoma cells SH-SY5Y, MEFS and ATG5−/− MEF [44]

**Chloroquine/ hydroxychloroquine** (Inhibition of Chloroquine activity under acidic stress [45])	**Anti-Malaria**<br>Inhibition of DNA/RNA synthesis via electrostatic forces<br>Binding of heme → toxicity against parasites [46]	**HCC:**<br>-As single treatment<br>Induces G0/G1 cell cycle arrest [47]<br>Leads to loss of $\Delta\Psi m$ → cleavage of Caspase 3 and PARP → intrinsic apoptosis [47]<br>Acts chemopreventive: Decreases p-AKT, AKT and NF-κB-p65 protein expression via TLR9 and TLR7 inhibition → inhibits HCC development in two HCC xenograft models [48]<br>**Melanoma:**<br>-As single treatment<br>Stabilizes PUMA protein → induces apoptosis in vivo and in vitro [49]<br>**Colon cancer:**<br>-As single treatment<br>Reduces phosphorylation of Akt and p44/42 MAPK → reduces proliferation and increases apoptosis in vivo and in vitro [50]<br>Synergizes with 5-FU via enhancement of G0/G1cell cycle arrest and 5-FU induced apoptosis [51]<br>**Melanoma and bladder cancer:**<br>-As single treatment<br>-Inhibits autophagic flux through inhibition of autophagosomes degradation and lysosomes fusion [52, 53]<br>**Pancreatic cancer:**<br>-As single treatment<br>-Inhibits CXCL12 binding to CXCR4 and inhibits downstream signaling [54]<br>-Inhibits autophagy → increases ROS production → increases DNA damage and alters cell metabolism [55]<br>**Lung Cancer**<br>-As single treatment<br>Increases volume of lysosomes → inhibits cell proliferation [56]<br>**Glioblastoma:**<br>-As single treatment<br>-Decreases invasiveness under hypoxic conditions [57]<br>-Stabilizes p53 → tp53-depenendet apoptosis → decreased growth in vivo and in vitro [58]	**Clinical trial Phase I:**<br>-Neoplasms (NCT02366884)<br>-Neoplasm in combination with Carboplatin and Gemcitabine (NCT02071537)<br>-Pancreatic in combination with Gemcitabine (NCT01777477)<br>-Glioblastoma (NCT04772846)<br>-Glioblastoma as radio sensitizer (NCT02378532)<br>-Glioma, Cholangiocarcinoma, Chondrosarcoma (NCT02496741)<br>-Carcinoma (NCT01023477)<br>-Glioblastoma in combination with Temozolomide as chemotherapeutic agent and radio sensitizer (NCT04397679)<br>-Results breast cancer study [59]<br><br>**Clinical trial Phase II:**<br>-Breast (NCT02333890)<br>-Breast in combination with chemotherapy (NCT01446016)<br>-Glioblastoma, astrocytoma (NCT02432417)<br>-Multiple myeloma in combination with Velcade and Cyclophosphamide (NCT01438177)<br>-Neoplasm in combination with other Anti-Protozoal Agents (NCT02366884)

**Chloroquine/ hydroxychloroquine** (Continued)		**Breast cancer:**<br>**-**In combination<br>Radio-sensitizes via increased lysosomal volume and decreased plasma membrane stability → increased necrosis [60]<br>Synergizes with Doxorubicin [61]<br>-As single treatment<br>Inhibits MMP9 and MMP2, but also elevates MMP13 expression and decreases TLR9 expression (tumor-promoting and tumor-inhibiting) [62]<br>Inhibits autophagy → decreases growth and survival in EGFR overexpressing cells [63]<br>Leads to loss of $\Delta\Psi m$ → cleavage of caspase 3 and 9 → induces apoptosis via intrinsic pathway [64, 65]<br>Acts anti-metastatic [64]<br>Induces G2/M cell cycle arrest via decreased polo like kinase 1 and phosphorylated cdc25C levels [65]<br>Breast cancer–protective effect via p53 and p21 activation: chloroquine dependent ATM phosphorylation → phosphorylation of p53 → G_1 cell cycle arrest in induced breast cancer models [66]<br>**Stem cells:**<br>-As single treatment<br>Increases apoptosis and decreases clonogenic capacity [67]<br><br>More extensive summary is presented in the summary of Munshin et al. [68]	**Clinical Trial Phase III:**<br>-Glioblastoma multiforme (NCT00224978)<br><br>***In vivo:***<br>-Glioblastoma cells U373-EGFRwt and U373 in NMRI-nu (nu/nu) mice [63]<br>-Glioblastoma cells U87MG in NMRI mice [58]<br>-Liver cancer cells Huh 7 in athymic BALB/c nu/nu [67]<br>-Liver cancer cells HepG2-GFP in nude mice [47]<br>-Liver cancer cells HepG2 and HuH7 in NOD-SCID mice [48]<br>-DEN-induced HCC model in Fischer F344 rats [48]<br>-Melanoma cells SKMel23 in NOD-SCID mice [49]<br>-NMU-induced mammary adenocarcinoma in Wistar-Furth rats [66]<br>-Breast cancer cells MCF7 and MDA-MB-231 in athymic nude/nu Foxn1 mice [62]<br>-Breast cancer cells 4T1 in BALB/c mice [64]<br>-Colon cancer cells HCT116 and HT29 in nude mice<br>-Colon cancer cells CT26 in BALB/c mice [50]<br>-Pancreatic cancer cells 8988T and panc1 in NCr nude mice [55]<br>-Lung cancer cells H460 in NCr nude mice [55]

Abbreviations: Acute myeloid leukemia (AML), DNA damage response (DDR), Doublestrand breaks (DSB), Doxorubicin (DOX), Head and neck cancer (HNC), Hepatocellular carcinoma (HCC), IkB Kinase (IKK), Mitochondrial membrane potential ($\Delta\Psi m$), Non-Hodgkin lymphoma (NHL), **Paclitaxel (PAX),** P-glycoprotein (P-gp), Poly (ADP-ribose)-Polymerase (PARP), Osteosarcoma (OSC), Renal cell carcinoma (RCC)

References

[1] Gopalakrishnan, A.M.; Kumar, N. Antimalarial action of artesunate involves DNA damage mediated by reactive oxygen species. *Antimicrobial agents and chemotherapy*, **2015**, *59*, 317–325.

[2] Li, Z.-J.; Dai, H.-Q.; Huang, X.-W.; Feng, J.; Deng, J.-H.; Wang, Z.-X.; Yang, X.-M.; Liu, Y.-J.; Wu, Y.; Chen, P.-H.; Shi, H.; Wang, J.-G.; Zhou, J.; Lu, G.-D. Artesunate synergizes with sorafenib to induce ferroptosis in hepatocellular carcinoma. *Acta pharmacologica Sinica*, **2021**, *42*, 301–310.

[3] Roh, J.-L.; Kim, E.H.; Jang, H.; Shin, D. Nrf2 inhibition reverses the resistance of cisplatin-resistant head and neck cancer cells to artesunate-induced ferroptosis. *Redox biology*, **2017**, *11*, 254–262.

[4] Vandewynckel, Y.-P.; Laukens, D.; Geerts, A.; Vanhove, C.; Descamps, B.; Colle, I.; Devisscher, L.; Bogaerts, E.; Paridaens, A.; Verhelst, X.; van Steenkiste, C.; Libbrecht, L.; Lambrecht, B.N.; Janssens, S.; van Vlierberghe, H. Therapeutic effects of artesunate in hepatocellular carcinoma: repurposing an ancient antimalarial agent. *European journal of gastroenterology & hepatology*, **2014**, *26*, 861–870.

[5] Da Jeong, E.; Song, H.J.J.; Lim, S.; Lee, S.J.J.; Lim, J.E.; Nam, D.-H.; Joo, K.M.; Jeong, B.C.; Jeon, S.S.; Choi, H.Y.; Lee, H.W. Repurposing the anti-malarial drug artesunate as a novel therapeutic agent for metastatic renal cell carcinoma due to its attenuation of tumor growth, metastasis, and angiogenesis. *Oncotarget*, **2015**, *6*, 33046–33064.

[6] Duarte, D.; Vale, N. New Trends for Antimalarial Drugs: Synergism between Antineoplastics and Antimalarials on Breast Cancer Cells. *Biomolecules*, **2020**, *10*.

[7] Chen, K.; Shou, L.-M.; Lin, F.; Duan, W.-M.; Wu, M.-Y.; Xie, X.; Xie, Y.-F.; Li, W.; Tao, M. Artesunate induces G2/M cell cycle arrest through autophagy induction in breast cancer cells. *Anti-cancer drugs*, **2014**, *25*, 652–662.

[8] Greenshields, A.L.; Fernando, W.; Hoskin, D.W. The anti-malarial drug artesunate causes cell cycle arrest and apoptosis of triple-negative MDA-MB-468 and HER2-enriched SK-BR-3 breast cancer cells. *Experimental and molecular pathology*, **2019**, *107*, 10–22.

[9] Hamacher-Brady, A.; Stein, H.A.; Turschner, S.; Toegel, I.; Mora, R.; Jennewein, N.; Efferth, T.; Eils, R.; Brady, N.R. Artesunate activates mitochondrial apoptosis in breast cancer cells via iron-catalyzed lysosomal reactive oxygen species production. *The Journal of biological chemistry*, **2011**, *286*, 6587–6601.

[10] Efferth, T.; Ramirez, T.; Gebhart, E.; Halatsch, M.-E. Combination treatment of glioblastoma multiforme cell lines with the anti-malarial artesunate and the epidermal growth factor receptor tyrosine kinase inhibitor OSI-774. *Biochemical pharmacology*, **2004**, *67*, 1689–1700.

[11] Reichert, S.; Reinboldt, V.; Hehlgans, S.; Efferth, T.; Rödel, C.; Rödel, F. A radiosensitizing effect of artesunate in glioblastoma cells is associated with a diminished expression of the inhibitor of apoptosis protein survivin. *Radiotherapy and oncology : journal of the European Society for Therapeutic Radiology and Oncology*, **2012**, *103*, 394–401.

[12] Berdelle, N.; Nikolova, T.; Quiros, S.; Efferth, T.; Kaina, B. Artesunate induces oxidative DNA damage, sustained DNA double-strand breaks, and the ATM/ATR damage response in cancer cells. *Molecular cancer therapeutics*, **2011**, *10*, 2224–2233.

[13] Zhang, P.; Luo, H.-S.; Li, M.; Tan, S.-Y. Artesunate inhibits the growth and induces apoptosis of human gastric cancer cells by downregulating COX-2. *OncoTargets and therapy*, **2015**, *8*, 845–854.

[14] Zhou, X.; Sun, W.-J.; Wang, W.-M.; Chen, K.; Zheng, J.-H.; Lu, M.-D.; Li, P.-H.; Zheng, Z.-Q. Artesunate inhibits the growth of gastric cancer cells through the mechanism of promoting oncosis both in vitro and in vivo. *Anti-cancer drugs*, **2013**, *24*, 920–927.

[15] Efferth, T.; Giaisi, M.; Merling, A.; Krammer, P.H.; Li-Weber, M. Artesunate induces ROS-mediated apoptosis in doxorubicin-resistant T leukemia cells. *PloS one*, **2007**, *2*, e693.

[16] Zhao, Y.; Jiang, W.; Li, B.; Yao, Q.; Dong, J.; Cen, Y.; Pan, X.; Li, J.; Zheng, J.; Pang, X.; Zhou, H. Artesunate enhances radiosensitivity of human non-small cell lung cancer A549 cells via increasing NO production to induce cell cycle arrest at G2/M phase. *International immunopharmacology*, **2011**, *11*, 2039–2046.

[17] Efferth, T.; Volm, M. Glutathione-related enzymes contribute to resistance of tumor cells and low toxicity in normal organs to artesunate. *In vivo (Athens, Greece)*, **2005**, *19*, 225–232.

[18] Dell'Eva, R.; Pfeffer, U.; Vené, R.; Anfosso, L.; Forlani, A.; Albini, A.; Efferth, T. Inhibition of angiogenesis in vivo and growth of Kaposi's sarcoma xenograft tumors by the anti-malarial artesunate. *Biochemical pharmacology*, **2004**, *68*, 2359–2366.

[19] Li, Q.; Ni, W.; Deng, Z.; Liu, M.; She, L.; Xie, Q. Targeting nasopharyngeal carcinoma by artesunate through inhibiting Akt/mTOR and inducing oxidative stress. *Fundamental & clinical pharmacology*, **2017**, *31*, 301–310.

[20] Du, J.-H.; Zhang, H.-D.; Ma, Z.-J.; Ji, K.-M. Artesunate induces oncosis-like cell death in vitro and has antitumor activity against pancreatic cancer xenografts in vivo. *Cancer chemotherapy and pharmacology*, **2010**, *65*, 895–902.

[21] Yang, N.-D.; Tan, S.-H.; Ng, S.; Shi, Y.; Zhou, J.; Tan, K.S.W.; Wong, W.-S.F.; Shen, H.-M. Artesunate induces cell death in human cancer cells via enhancing lysosomal function and lysosomal degradation of ferritin. *The Journal of biological chemistry*, **2014**, *289*, 33425–33441.

[22] Ramirez, I.; Marchion, D.; Xiong, Y.; Abbasi, F.; Boac, B.; McClung, C.; Al Sawah, E.; Judson, P.; Apte, S.M.; Lancaster, J.M. Repurposing Artesunate, an anti-malarial, for ovarian cancer treatment: Mechanistic insights from cell line models. *Gynecologic Oncology*, **2015**, *137*, 207.

[23] Wang, Z.-C.; Liu, Y.; Wang, H.; Han, Q.-K.; Lu, C. Research on the relationship between artesunate and Raji cell autophagy and apoptosis of Burkitt's lymphoma and its mechanism. *European review for medical and pharmacological sciences*, **2017**, *21*, 2238–2243.

[24] Thanaketpaisarn, O.; Waiwut, P.; Sakurai, H.; Saiki, I. Artesunate enhances TRAIL-induced apoptosis in human cervical carcinoma cells through inhibition of the NF-κB and PI3K/Akt signaling pathways. *Int J Oncol*, **2011**, *39*, 279–285.

[25] Augustin, Y.; Staines, H.M.; Krishna, S. Artemisinins as a novel anti-cancer therapy: Targeting a global cancer pandemic through drug repurposing. *Pharmacology & therapeutics*, **2020**, *216*, 107706.

[26] Li, Z.; Li, Q.; Wu, J.; Wang, M.; Yu, J. Artemisinin and Its Derivatives as a Repurposing Anticancer Agent: What Else Do We Need to Do? *Molecules (Basel, Switzerland)*, **2016**, *21*.

[27] Xu, C.; Zhang, H.; Mu, L.; Yang, X. Artemisinins as Anticancer Drugs: Novel Therapeutic Approaches, Molecular Mechanisms, and Clinical Trials. *Frontiers in pharmacology*, **2020**, *11*, 529881.

[28] Sun, X.; Yan, P.; Zou, C.; Wong, Y.-K.; Shu, Y.; Lee, Y.M.; Zhang, C.; Yang, N.-D.; Wang, J.; Zhang, J. Targeting autophagy enhances the anticancer effect of artemisinin and its derivatives. *Medicinal research reviews*, **2019**, *39*, 2172–2193.

[29] Efferth, T.; Sauerbrey, A.; Olbrich, A.; Gebhart, E.; Rauch, P.; Weber, H.O.; Hengstler, J.G.; Halatsch, M.-E.; Volm, M.; Tew, K.D.; Ross, D.D.; Funk, J.O. Molecular modes of action of artesunate in tumor cell lines. *Molecular pharmacology*, **2003**, *64*, 382–394.

[30] Efferth, T.; Dunstan, H.; Sauerbrey, A.; Miyachi, H.; Chitambar, C.R. The anti-malarial artesunate is also active against cancer. *International journal of oncology*, **2001**, *18*, 767–773.

[31] Efferth, T.; Briehl, M.; Tome, M. Role of antioxidant genes for the activity of artesunate against tumor cells. *Int J Oncol*, **2003**.

[32] Wong, W.; Bai, X.-C.; Sleebs, B.E.; Triglia, T.; Brown, A.; Thompson, J.K.; Jackson, K.E.; Hanssen, E.; Marapana, D.S.; Fernandez, I.S.; Ralph, S.A.; Cowman, A.F.; Scheres, S.H.W.; Baum, J. Mefloquine targets the Plasmodium falciparum 80S ribosome to inhibit protein synthesis. *Nature microbiology*, **2017**, *2*, 17031.

[33] Sharma, N.; Thomas, S.; Golden, E.B.; Hofman, F.M.; Chen, T.C.; Petasis, N.A.; Schönthal, A.H.; Louie, S.G. Inhibition of autophagy and induction of breast cancer cell death by mefloquine, an antimalarial agent. *Cancer letters*, **2012**, *326*, 143–154.

[34] Kim, J.-H.; Choi, A.-R.; Kim, Y.K.; Yoon, S. Co-treatment with the anti-malarial drugs mefloquine and primaquine highly sensitizes drug-resistant cancer cells by increasing P-gp inhibition. *Biochemical and biophysical research communications*, **2013**, *441*, 655–660.

[35] Fujita, R.; Ishikawa, M.; Takayanagi, M.; Takayanagi, Y.; Sasaki, K. Enhancement of doxorubicin activity in multidrug-resistant cells by mefloquine. *Methods and findings in experimental and clinical pharmacology*, **2000**, *22*, 281–284.

[36] Yan, K.-H.; Lin, Y.-W.; Hsiao, C.-H.; Wen, Y.-C.; Lin, K.-H.; Liu, C.-C.; Hsieh, M.-C.; Yao, C.-J.; Yan, M.-D.; Lai, G.-M.; Chuang, S.-E.; Lee, L.-M. Mefloquine induces cell death in prostate cancer cells and provides a potential novel treatment strategy in vivo. *Oncology letters*, **2013**, *5*, 1567–1571.

[37] Yan, K.-H.; Yao, C.-J.; Hsiao, C.-H.; Lin, K.-H.; Lin, Y.-W.; Wen, Y.-C.; Liu, C.-C.; Yan, M.-D.; Chuang, S.-E.; Lai, G.-M.; Lee, L.-M. Mefloquine exerts anticancer activity in prostate cancer cells via ROS-mediated modulation of Akt, ERK, JNK and AMPK signaling. *Oncology letters*, **2013**, *5*, 1541–1545.

[38] Liu, Y.; Chen, S.; Xue, R.; Zhao, J.; Di, M. Mefloquine effectively targets gastric cancer cells through phosphatase-dependent inhibition of PI3K/Akt/mTOR signaling pathway. *Biochemical and biophysical research communications*, **2016**, *470*, 350–355.

[39] Maraka, S.; Groves, M.D.; Mammoser, A.G.; Melguizo-Gavilanes, I.; Conrad, C.A.; Tremont-Lukats, I.W.; Loghin, M.E.; O'Brien, B.J.; Puduvalli, V.K.; Sulman, E.P.; Hess, K.R.; Aldape, K.D.; Gilbert, M.R.; Groot, J.F. de; Alfred Yung, W.K.; Penas-Prado, M. Phase 1 lead-in to a phase 2 factorial study of temozolomide plus memantine, mefloquine, and metformin as postradiation adjuvant therapy for newly diagnosed glioblastoma. *Cancer*, **2019**, *125*, 424–433.
[40] Li, H.; Jiao, S.; Li, X.; Banu, H.; Hamal, S.; Wang, X. Therapeutic effects of antibiotic drug mefloquine against cervical cancer through impairing mitochondrial function and inhibiting mTOR pathway. *Canadian journal of physiology and pharmacology*, **2017**, *95*, 43–50.
[41] Li, Y.-H.; Yang, S.-L.; Zhang, G.-F.; Wu, J.-C.; Gong, L.-L.; Ming-Zhong; Lin, R.-X. Mefloquine targets β-catenin pathway and thus can play a role in the treatment of liver cancer. *Microbial pathogenesis*, **2018**, *118*, 357–360.
[42] Xu, X.; Wang, J.; Han, K.; Li, S.; Xu, F.; Yang, Y. Antimalarial drug mefloquine inhibits nuclear factor kappa B signaling and induces apoptosis in colorectal cancer cells. *Cancer science*, **2018**, *109*, 1220–1229.
[43] Sukhai, M.A.; Prabha, S.; Hurren, R.; Rutledge, A.C.; Lee, A.Y.; Sriskanthadevan, S.; Sun, H.; Wang, X.; Skrtic, M.; Seneviratne, A.; Cusimano, M.; Jhas, B.; Gronda, M.; MacLean, N.; Cho, E.E.; Spagnuolo, P.A.; Sharmeen, S.; Gebbia, M.; Urbanus, M.; Eppert, K.; Dissanayake, D.; Jonet, A.; Dassonville-Klimpt, A.; Li, X.; Datti, A.; Ohashi, P.S.; Wrana, J.; Rogers, I.; Sonnet, P.; Ellis, W.Y.; Corey, S.J.; Eaves, C.; Minden, M.D.; Wang, J.C.Y.; Dick, J.E.; Nislow, C.; Giaever, G.; Schimmer, A.D. Lysosomal disruption preferentially targets acute myeloid leukemia cells and progenitors. *The Journal of clinical investigation*, **2013**, *123*, 315–328.
[44] Shin, J.H.; Park, S.J.; Jo, Y.K.; Kim, E.S.; Kang, H.; Park, J.-H.; Lee, E.H.; Cho, D.-H. Suppression of autophagy exacerbates Mefloquine-mediated cell death. *Neuroscience letters*, **2012**, *515*, 162–167.
[45] Pellegrini, P.; Strambi, A.; Zipoli, C.; Hägg-Olofsson, M.; Buoncervello, M.; Linder, S.; Milito, A. de. Acidic extracellular pH neutralizes the autophagy-inhibiting activity of chloroquine: implications for cancer therapies. *Autophagy*, **2014**, *10*, 562–571.
[46] Coban, C. The host targeting effect of chloroquine in malaria. *Current opinion in immunology*, **2020**, *66*, 98–107.
[47] Hu, T.; Li, P.; Luo, Z.; Chen, X.; Zhang, J.; Wang, C.; Chen, P.; Dong, Z. Chloroquine inhibits hepatocellular carcinoma cell growth in vitro and in vivo. *Oncol Rep*, **2016**, *35*, 43–49.
[48] Mohamed, F.E.; Al-Jehani, R.M.; Minogue, S.S.; Andreola, F.; Winstanley, A.; Olde Damink, S.W.M.; Habtesion, A.; Malagó, M.; Davies, N.; Luong, T.V.; Dhillon, A.P.; Mookerjee, R.P.; Dhar, D.K.; Jalan, R. Effect of toll-like receptor 7 and 9 targeted therapy to prevent the development of hepatocellular carcinoma. *Liver international : official journal of the International Association for the Study of the Liver*, **2015**, *35*, 1063–1076.
[49] Lakhter, A.J.; Sahu, R.P.; Sun, Y.; Kaufmann, W.K.; Androphy, E.J.; Travers, J.B.; Naidu, S.R. Chloroquine promotes apoptosis in melanoma cells by inhibiting BH3 domain-mediated PUMA degradation. *The Journal of investigative dermatology*, **2013**, *133*, 2247–2254.
[50] Zheng, Y.; Zhao, Y.-L.; Deng, X.; Yang, S.; Mao, Y.; Li, Z.; Jiang, P.; Zhao, X.; Wei, Y. Chloroquine inhibits colon cancer cell growth in vitro and tumor growth in vivo via induction of apoptosis. *Cancer investigation*, **2009**, *27*, 286–292.
[51] Sasaki, K.; Tsuno, N.H.; Sunami, E.; Tsurita, G.; Kawai, K.; Okaji, Y.; Nishikawa, T.; Shuno, Y.; Hongo, K.; Hiyoshi, M.; Kaneko, M.; Kitayama, J.; Takahashi, K.; Nagawa, H. Chloroquine potentiates the anti-cancer effect of 5-fluorouracil on colon cancer cells. *BMC cancer*, **2010**, *10*, 370.
[52] Lin, Y.-C.; Lin, J.-F.; Wen, S.-I.; Yang, S.-C.; Tsai, T.-F.; Chen, H.-E.; Chou, K.-Y.; Hwang, T.I.-S. Chloroquine and hydroxychloroquine inhibit bladder cancer cell growth by targeting basal autophagy and enhancing apoptosis. *The Kaohsiung Journal of Medical Sciences*, **2017**, *33*, 215–223.
[53] Maes, H.; Martin, S.; Verfaillie, T.; Agostinis, P. Dynamic interplay between autophagic flux and Akt during melanoma progression in vitro. *Experimental dermatology*, **2014**, *23*, 101–106.
[54] Kim, J.; Yip, M.L.R.; Shen, X.; Li, H.; Hsin, L.-Y.C.; Labarge, S.; Heinrich, E.L.; Lee, W.; Lu, J.; Vaidehi, N. Identification of anti-malarial compounds as novel antagonists to chemokine receptor CXCR4 in pancreatic cancer cells. *PloS one*, **2012**, *7*, e31004.
[55] Yang, S.; Wang, X.; Contino, G.; Liesa, M.; Sahin, E.; Ying, H.; Bause, A.; Li, Y.; Stommel, J.M.; Dell'antonio, G.; Mautner, J.; Tonon, G.; Haigis, M.; Shirihai, O.S.; Doglioni, C.; Bardeesy, N.; Kimmelman, A.C. Pancreatic cancers require autophagy for tumor growth. *Genes & development*, **2011**, *25*, 717–729.

[56] Fan, C.; Wang, W.; Zhao, B.; Zhang, S.; Miao, J. Chloroquine inhibits cell growth and induces cell death in A549 lung cancer cells. *Bioorganic & medicinal chemistry*, **2006**, *14*, 3218–3222.

[57] Sandholm, J.; Tuomela, J.; Kauppila, J.H.; Harris, K.W.; Graves, D.; Selander, K.S. Hypoxia regulates Toll-like receptor-9 expression and invasive function in human brain cancer cells in vitro. *Oncology letters*, **2014**, *8*, 266–274.

[58] Kim, E.L.; Wüstenberg, R.; Rübsam, A.; Schmitz-Salue, C.; Warnecke, G.; Bücker, E.-M.; Pettkus, N.; Speidel, D.; Rohde, V.; Schulz-Schaeffer, W.; Deppert, W.; Giese, A. Chloroquine activates the p53 pathway and induces apoptosis in human glioma cells. *Neuro-oncology*, **2010**, *12*, 389–400.

[59] Arnaout, A.; Robertson, S.J.; Pond, G.R.; Lee, H.; Jeong, A.; Ianni, L.; Kroeger, L.; Hilton, J.; Coupland, S.; Gottlieb, C.; Hurley, B.; McCarthy, A.; Clemons, M. A randomized, double-blind, window of opportunity trial evaluating the effects of chloroquine in breast cancer patients. *Breast cancer research and treatment*, **2019**, *178*, 327–335.

[60] Zhao, H.; Cai, Y.; Santi, S.; Lafrenie, R.; Lee, H. Chloroquine-mediated radiosensitization is due to the destabilization of the lysosomal membrane and subsequent induction of cell death by necrosis. *Radiation research*, **2005**, *164*, 250–257.

[61] Maycotte, P.; Gearheart, C.M.; Barnard, R.; Aryal, S.; Mulcahy Levy, J.M.; Fosmire, S.P.; Hansen, R.J.; Morgan, M.J.; Porter, C.C.; Gustafson, D.L.; Thorburn, A. STAT3-mediated autophagy dependence identifies subtypes of breast cancer where autophagy inhibition can be efficacious. *Cancer research*, **2014**, *74*, 2579–2590.

[62] Tuomela, J.; Sandholm, J.; Kauppila, J.H.; Lehenkari, P.; Harris, K.W.; Selander, K.S. Chloroquine has tumor-inhibitory and tumor-promoting effects in triple-negative breast cancer. *Oncology letters*, **2013**, *6*, 1665–1672.

[63] Jutten, B.; Keulers, T.G.; Schaaf, M.B.E.; Savelkouls, K.; Theys, J.; Span, P.N.; Vooijs, M.A.; Bussink, J.; Rouschop, K.M.A. EGFR overexpressing cells and tumors are dependent on autophagy for growth and survival. *Radiotherapy and oncology : journal of the European Society for Therapeutic Radiology and Oncology*, **2013**, *108*, 479–483.

[64] Jiang, P.-D.; Zhao, Y.-L.; Deng, X.-Q.; Mao, Y.-Q.; Shi, W.; Tang, Q.-Q.; Li, Z.-G.; Zheng, Y.-Z.; Yang, S.-Y.; Wei, Y.-Q. Antitumor and antimetastatic activities of chloroquine diphosphate in a murine model of breast cancer. *Biomedicine & pharmacotherapy = Biomedecine & pharmacotherapie*, **2010**, *64*, 609–614.

[65] Jiang, P.-D.; Zhao, Y.-L.; Shi, W.; Deng, X.-Q.; Xie, G.; Mao, Y.-Q.; Li, Z.-G.; Zheng, Y.-Z.; Yang, S.-Y.; Wei, Y.-Q. Cell growth inhibition, G2/M cell cycle arrest, and apoptosis induced by chloroquine in human breast cancer cell line Bcap-37. *Cellular physiology and biochemistry : international journal of experimental cellular physiology, biochemistry, and pharmacology*, **2008**, *22*, 431–440.

[66] Loehberg, C.R.; Thompson, T.; Kastan, M.B.; Maclean, K.H.; Edwards, D.G.; Kittrell, F.S.; Medina, D.; Conneely, O.M.; O'Malley, B.W. Ataxia telangiectasia-mutated and p53 are potential mediators of chloroquine-induced resistance to mammary carcinogenesis. *Cancer research*, **2007**, *67*, 12026–12033.

[67] Song, Y.-J.; Zhang, S.-S.; Guo, X.-L.; Sun, K.; Han, Z.-P.; Li, R.; Zhao, Q.-D.; Deng, W.-J.; Xie, X.-Q.; Zhang, J.-W.; Wu, M.-C.; Wei, L.-X. Autophagy contributes to the survival of CD133+ liver cancer stem cells in the hypoxic and nutrient-deprived tumor microenvironment. *Cancer letters*, **2013**, *339*, 70–81.

[68] Munshi, A. Chloroquine in glioblastoma--new horizons for an old drug. *Cancer*, **2009**, *115*, 2380–2383.
