# Supplementary material for: Repurposing of Antimicrobial Agents for Cancer Therapy: What Do We Know?
Source: Cancers (Basel). 2021 Jun 26;13(13):3193. doi: 10.3390/cancers13133193 (PMC8269327; doi:10.3390/cancers13133193)
Supplement: Supplementary file 1 [file cancers-13-03193-s001.zip › Supplemental Data table S5.pdf]

Table S5. List of anthelmintic agents that are currently being investigated in clinical trials for their effectiveness against different cancer types when alone and/or in combination with chemotherapeutic agents as well as anthelmintics that are potential new anti-cancer drugs shown to inhibit malignant cells *in vivo*/*in vitro*. The summarized data is obtained from clinicaltrials.gov, the National Institute of Health (NIH), the Drugbank, and the ReDo databank. PubMed and Google Scholar have been used to gain detailed information about the mechanism(s) of action of the potential repurposed drugs *in vitro* and *in vivo*. The keywords used for the search are "anti-cancer" and "repurposed".

**Drug**	**Original application / target**	**New application (anti-cancer) / Proposed Target/Mechanism of action**	**Stage of development**
**Mebendazole**	**Anthelmintic**<br>Binds to tubulin – inhibition of tubulin formation [1]	**Meningioma:**<br>-As single treatment:<br>Inhibits colony formation, activates caspase-3, reduces angiogenesis [2]<br>-In combination:<br>Enhances radiation' effect (radiosensitizer) [2]<br>**Lung cancer:**<br>-As single treatment:<br>Stimulates CD14+ myeloid cells → enhances T-cell activation →<br>enhances tumor cell killing/induces apoptosis [3]<br>**Medulla blastoma:**<br>-As single treatment:<br>Suppresses primary cilium formation (tubulin based organelle,<br>signaling hub for the Hedgehog pathway) → inhibits Hedgehog<br>pathway → inhibits cell growth [4]<br>Stimulates immune system → increases TNFα and IFN-γ [3]	**Clinical trials Phase III:**<br>-Cancer overall survival (NCT02201381)<br>-Neoplasms (NCT02366884)<br><br>*In combination*<br>-Colorectal cancer – Mebendazole with FOLFOX (NCT03925662)<br><br>**Clinical trials Phase II:**<br>-Cancer of unknown origin (NCT03628079)<br><br>**Clinical trials Phase I:**<br>-Recurrent pediatric brain cancers (NCT02644291)<br>-Cancer of the gastrointestinal tract (NCT03628079)<br>-High grade glioma (NCT01729260)<br>-Pilomyxoid Astrocytoma (NCT01837862)<br><br>***In vivo:***<br>-Female athymic mice with meningioma cells KT21MG1 cells [2]<br><br>***In vitro:***<br>-Meningioma cells KT21MG1, IOMM LEE, AC-1, SF4068, SF6717, SF1335, SF1335 + YAP [2]<br>-Medulla blastoma cells 293T and hTERT-RPE1 [4]

**Niclosanide**	**Anthelmintic**<br>Inhibition glucose uptake by worms [5]	**Renal cancer:**<br>**-**As single treatment:<br>Inhibits C-MYC and E2F1 expression, enhances PTEN expression, inhibits cell proliferation, migration and progression, and induces apoptosis [6]<br>-In combination:<br>Synergizes Sorafenib's effect [6]<br>**Adeno cortical carcinoma:**<br>**-**As single treatment:<br>Inhibits tumor growth and proliferation, induces cell cycle arrest and apoptosis; decreases cellular migration, downregulates the level of β-catenin [7]<br>**Glioblastoma:**<br>**-**As single treatment:<br>Induces cell arrest and decreases cell viability, by inhibiting tubulin formation [8]<br>**Lung cancer:**<br>-In combination:<br>Enhances radiation' effect (radiosensitizer) [9]	**Clinical trial phase II:**<br>-Prostate cancer (NCT02807805)<br>-Familial Adenomatous Polyposis (NCT04296851)<br><br>**Clinical trial phase I:**<br>-Prostate cancer (NCT03123978)<br>-Enzalutamine with Niclosamide, prostate cancer (NCT02532114)<br><br>***In vivo:***<br>-Nude mice with renal cancer cells Caki-1 [6]<br>-Adeno cortical carcinoma cells NCI-H295R in Nu^+/Nu^+ mice [7]<br>-Female C57BL/6 mice with glioblastoma cells GL-261 [8]<br><br>***In vitro:***<br>-Renal cancer cells A498 and Caki-1 [6]<br>-Adeno cortical carcinoma cells NCI-H295R and SW-13 [7]<br>-Glioblastoma cells GL-261 [8]<br>-Lung cancer cells H1299 [9]
**Ivermectin**	**Anthelmintic**<br>Modulator of the γ-aminobutyric acid (GABA) receptor activity or glutamate-gated chloride ion channels (Glu-Cl) [10–12]	**Ovarian cancer:**<br>**-**As single treatment:<br>Induces DNA damage – double-strand breaks, breaks down mitochondrial membrane, upregulates BAX/BCL-2, releases free cytochrome C in cytosol, inhibits cell viability, induces apoptosis via caspase-9/3 activation [11]<br>**Liver cancer:**<br>**-**As single treatment:<br>Induces morphological changes – DNA fragmentation and chromatin condensation, produces ROS, decreases mitochondrial membrane potential, upregulates BAX/BCL-2, induces apoptosis via caspase-9/3 activation [12]	**Clinical Phase II:**<br>-Neoplasms (NCT02366884)<br>**In vivo:**<br>-MDAY-D2 murine leukemia cells in mice [13]<br>-Glioblastoma cancer xenograft in SCID mice [14]<br>-Female NOD/SCID and Balb/c mice with breast cancer cells MDA-MB-231 [15]

**Ivermectin**<br>*(Continued)*		**Leukemia:**<br>**-**As single treatment:<br>Increases intracellular chloride ion concentration – plasma membrane hyperpolarization, enhances ROS level, and induces cell death [13]<br>**Glioma, Melanoma, and Bronchio-alveolar carcinoma :**<br>**-**As single treatment:<br>Blocks WNT-TCF signaling, inhibits cell proliferation, induces apoptosis; *in vivo* only active against TCF dependent cancer types [16]<br>**Glioblastoma:**<br>**-**As single treatment:<br>Inhibits angiogenesis, induces mitochondrial stress and enhances ROS levels, deactivates Akt/mTOR signaling pathway [14]<br>**Breast cancer:**<br>**-**As single treatment:<br>Inhibits cell growth, downregulates self-renewal transcription factors such as homeobox protein nanog (nanog), octamer-binding protein 4 (oct-4) and SRY-box 2 (sox-2) [17]<br>Activates cytostatic autophagy, decreases P21-activated kinase 1 (PAK1) expression → blocks Akt/mTOR signaling pathway [15]	***In vitro:***<br>-Ovarian cancer cells HeLa [11]<br>-Liver cancer cells HepG2 [12]<br>-Leukemia cells OCI-AML2, HL60, U937, KG1a, MDAY-D2 [13]<br>-Human colon cancer cells CC14, CC36, Ls174T, HT-29, and DLD-1<br>-Bronchio-alveolar carcinoma H358, 293T cells, GBM primary cells, U251 glioma, and SKMel2 melanoma cells [16]<br>-Glioblastoma cells A172 and U138-MG [14]<br>-Breast cancer cells MDA-MB-231 [17], MDA-MB-435, HS578T, 4T1, and HEK 293T [15]
**Praziquantel**	**Anthelmintic**<br>Induction of spastic paralysis of the worm musculature - related to the Ca^{2+} level [18, 19]	**Colon cancer, breast cancer, lung cancer, cervical cancer:**<br>-In combination:<br>Synergizes Paclitaxel's effects – inhibition of cell growth [20]	**Clinical trial Phase II:**<br>-Neoplasms – single treatment (NCT02366884)<br><br>***In vivo:***<br>-DLD-1 cells in athymic nude mice [20]<br><br>***In vitro:***<br>-Colon cancer cells DLD-1, breast cancer cells ZR-7530, lung cancer cells SPC-A-1 and Ltep-a-2, cervical cancer cells HeLa and breast cancer cells Bcap37 [20]

**Nitazoxamide**	**Anthelmintic**<br>Inhibition of pyruvate-ferredoxin oxidoreductase - down-regulates anaerobic metabolism [21, 22]	**Colon cancer:**<br>**-**As single treatment:<br>Interacts with glutathione-S-transferase P1 (GSTP1), inhibits cell growth, and induces nuclear condensation/DNA fragmentation/apoptosis [23]<br>Activates the AMPK pathway and downregulates the c-Myc, mTOR, and Wnt signaling [24]<br>**Glioblastoma:**<br>**-**As single treatment:<br>Affects autophagy regulation by blockage of late-stage lysosome<br>acidification, suppresses cell growth and induces cell cycle arrest<br>via upregulation of inhibitor growth protein 1 (ING1) expression [25]<br>**Breast cancer:**<br>**-**As single treatment:<br>Inhibits c-Myc, suppresses tumor growth, and induces apoptosis [26]	**Clinical trial Phase II:**<br>-Neoplasms (NCT02366884)<br><br>***In vivo:***<br>-Glioblastoma cells LN229 in ALB/C nude mice[25]<br>-Female NMRI nu/nu mice with colon cancer cells HCT-116 cells [24]<br><br>***In vitro:***<br>-Colon cancer cells Caco-2 [23], HCT-116 and HT29 [24]<br>-Glioblastoma cells LN229, A172, U87, and HUVEC [25]<br>-Breast cancer cells SKBR3 [26]
**Albendazole**	**Anthelmintic**<br>Inhibition of tubulin formation [1]	**Ovarian cancer:**<br>**-**As single treatment:<br>Inhibits cell proliferation and colony formation [27]<br>Disrupts tubulin formation, inhibits tumor growth and angiogenesis [28]<br><br>As BSA-Albendazol nano-particles:<br>Inhibits vascular endothelial growth factor [29]	**Clinical trial Phase II:**<br>-Neoplasms (NCT02366884)<br>***In vivo:***<br>-Female athymic nude mice with ovarian cancer cells OVCAR3 [29]<br>***In vitro:***<br>-Ovarian cancer cells 1A9, OVCAR-3 and SKOV-3 [27], OVCAR3 and SKOV3 [29], 1A9PTX22 [28]

**Levamisole**	**Anthelmintic**<br>Agonist of nicotinic acetylcholine receptors [30–32]	**Myeloma:**<br>**-**As single treatment:<br>Inhibits CD138 expression (transmembrane heparin sulfate glycoprotein, standard marker to identify tumor cells), increases<br>IL-6 secretion [33]<br>**Lung cancer:**<br>**-**As single treatment:<br>Inhibits cJNK phosphorylation, induces cell cycle arrest and tumor necrosis factor-related apoptosis [34]	**Clinical trials phase III:**<br>-Colon cancer – Levamisol hydrochloride in combination with 5-fluorouracil and leucovorin calcium (NCT00002593, NCT00425152, NCT00002551)<br>-Colon cancer – Levamisol in combination with 5-flurouracil and interleukin (NCT00309530, NCT00003063)<br>-Intrahepatic Cholangiocarcinoma (NCT03940378)<br><br>**Clinical trials phase II:**<br>-Neoplasms (NCT02366884)<br><br>***In vivo:***<br>-Lung cancer cells H460 in female BALB/c nude mice [34]<br><br>***In vitro***:<br>-Myeloma cells and U266 B1 [33]<br>-Lung cancer cells HCC827, H1975, H157, H460, and A549 [34]
**Pyrvinium**	**Anthelmintic**<br>Inhibition of glucose uptake [35]	**Breast cancer:**<br>**-**As single treatment:<br>Inhibits cell proliferation → inhibits Wnt pathway, suppresses EMT marker expression, and inhibits tumor cell self-renewal [36]<br><br>**Colon cancer:**<br>**-**As single treatment:<br>Inhibits cell viability, downregulates Wnt signaling mRNA transcription and protein expression, induces p21 [37]<br>-In combination:<br>Enhances 5-fluorouracil' effect [37]<br>**Various cancer cells:**<br>**-**As single treatment:<br>Inhibits autophagy [38]	***In vivo:***<br>-Breast cancer cells MDA-MB-231 in NOD/SCID mice [36]<br>-Healthy female mice with breast cancer cells 4T1 [38]<br>-Nude mice with colon cancer cells HCT-116 [37]

**Pyrvinium** *(continued)*		**Cancer stem-like cells:**<br>**-**As single treatment:<br>Inhibits lipid anabolism, impairs anabolic flux from glucose to cholesterol and fatty acids [39]<br>**Breast, DCIS, ovarian, prostate, lung, pancreatic, melanoma, and glioblastoma:**<br>-As single treatment:<br>Disrupts mitochondrial biogenesis [40]<br>Intercalates into DNA [41]	***In vitro:***<br>-Breast cancer cells MCF-7, T47D, MDA-MB-231, MDA-MB-468, Her-2, MCF10.DCIS, and SkBr-3 [36]<br>-Colon cancer cells HCT-25, HCT-116, LS174T, HT29, SW620, T84 [37]<br>-Cervical cancer cells HeLa, HeLa-GFP-LC3 [38]<br>-Kidney cancer cells HEK293[38]<br>-Ovarian cancer SKOV3, Tov21G, ES2 [40]<br>-Prostate cancer cells P3C [40]<br>-Pancreas cancer cells Ma PaCa2 [36], PANC1 [38]<br>-Lung caner cells A549 [36]-Melanoma cells A379 [36]<br>-Glioma cells U-87 MG [36]<br>[40]

References

[1] Martin, R.J. Modes of action of anthelmintic drugs. *The Veterinary Journal*, **1997**, *154*, 11–34.

[2] Skibinski, C.G.; Williamson, T.; Riggins, G.J. Mebendazole and radiation in combination increase survival through anticancer mechanisms in an intracranial rodent model of malignant meningioma. *Journal of neuro-oncology*, **2018**, *140*, 529–538.

[3] Rubin, J.; Mansoori, S.; Blom, K.; Berglund, M.; Lenhammar, L.; Andersson, C.; Loskog, A.; Fryknäs, M.; Nygren, P.; Larsson, R. Mebendazole stimulates CD14+ myeloid cells to enhance T-cell activation and tumour cell killing. *Oncotarget*, **2018**, *9*, 30805–30813.

[4] Larsen, A.R.; Bai, R.-Y.; Chung, J.H.; Borodovsky, A.; Rudin, C.M.; Riggins, G.J.; Bunz, F. Repurposing the antihelmintic mebendazole as a hedgehog inhibitor. *Molecular cancer therapeutics*, **2015**, *14*, 3–13.

[5] Märtlbauer, E. *Niclosamid;* Thieme Gruppe, **2008**.

[6] Yu, X.; Liu, F.; Zeng, L.; He, F.; Zhang, R.; Yan, S.; Zeng, Z.; Shu, Y.; Zhao, C.; Wu, X.; Lei, J.; Zhang, W.; Yang, C.; Wu, K.; Wu, Y.; An, L.; Huang, S.; Ji, X.; Gong, C.; Yuan, C.; Zhang, L.; Feng, Y.; Huang, B.; Liu, W.; Zhang, B.; Dai, Z.; Wang, X.; Liu, B.; Haydon, R.C.; Luu, H.H.; Gan, H.; He, T.-C.; Chen, L. Niclosamide Exhibits Potent Anticancer Activity and Synergizes with Sorafenib in Human Renal Cell Cancer Cells. *Cellular physiology and biochemistry : international journal of experimental cellular physiology, biochemistry, and pharmacology*, **2018**, *47*, 957–971.

[7] Satoh, K.; Zhang, L.; Zhang, Y.; Chelluri, R.; Boufraqech, M.; Nilubol, N.; Patel, D.; Shen, M.; Kebebew, E. Identification of Niclosamide as a Novel Anticancer Agent for Adrenocortical Carcinoma. *Clinical cancer research : an official journal of the American Association for Cancer Research*, **2016**, *22*, 3458–3466.

[8] Witt, M. de; Gamble, A.; Hanson, D.; Markowitz, D.; Powell, C.; Al Dimassi, S.; Atlas, M.; Boockvar, J.; Ruggieri, R.; Symons, M. Repurposing Mebendazole as a Replacement for Vincristine for the Treatment of Brain Tumors. *Molecular medicine (Cambridge, Mass.)*, **2017**, *23*, 50–56.

[9] Lee, S.; Son, A.-R.; Ahn, J.; Song, J.-Y. Niclosamide enhances ROS-mediated cell death through c-Jun activation. *Biomedicine & pharmacotherapy = Biomedecine & pharmacotherapie*, **2014**, *68*, 619–624.

[10] Bai, S.H.; Ogbourne, S. Eco-toxicological effects of the avermectin family with a focus on abamectin and ivermectin. *Chemosphere*, **2016**, *154*, 204–214.

[11] Zhang, Y.; Luo, M.; Xu, W.; Yang, M.; Wang, B.; Gao, J.; Li, Y.; Tao, L. Avermectin Confers Its Cytotoxic Effects by Inducing DNA Damage and Mitochondria-Associated Apoptosis. *Journal of agricultural and food chemistry*, **2016**, *64*, 6895–6902.

[12] Zhang, Y.; Wu, J.; Xu, W.; Gao, J.; Cao, H.; Yang, M.; Wang, B.; Hao, Y.; Tao, L. Cytotoxic effects of Avermectin on human HepG2 cells in vitro bioassays. *Environmental pollution (Barking, Essex : 1987)*, **2017**, *220*, 1127–1137.

[13] Sharmeen, S.; Skrtic, M.; Sukhai, M.A.; Hurren, R.; Gronda, M.; Wang, X.; Fonseca, S.B.; Sun, H.; Wood, T.E.; Ward, R.; Minden, M.D.; Batey, R.A.; Datti, A.; Wrana, J.; Kelley, S.O.; Schimmer, A.D. The antiparasitic agent ivermectin induces chloride-dependent membrane hyperpolarization and cell death in leukemia cells. *Blood*, **2010**, *116*, 3593–3603.

[14] Liu, Y.; Fang, S.; Sun, Q.; Liu, B. Anthelmintic drug ivermectin inhibits angiogenesis, growth and survival of glioblastoma through inducing mitochondrial dysfunction and oxidative stress. *Biochemical and biophysical research communications*, **2016**, *480*, 415–421.

[15] Dou, Q.; Chen, H.-N.; Wang, K.; Yuan, K.; Lei, Y.; Li, K.; Lan, J.; Chen, Y.; Huang, Z.; Xie, N.; Zhang, L.; Xiang, R.; Nice, E.C.; Wei, Y.; Huang, C. Ivermectin Induces Cytostatic Autophagy by Blocking the PAK1/Akt Axis in Breast Cancer. *Cancer research*, **2016**, *76*, 4457–4469.

[16] Melotti, A.; Mas, C.; Kuciak, M.; Lorente-Trigos, A.; Borges, I.; Ruiz i Altaba, A. The river blindness drug Ivermectin and related macrocyclic lactones inhibit WNT-TCF pathway responses in human cancer. *EMBO molecular medicine*, **2014**, *6*, 1263–1278.

[17] Dominguez-Gomez, G.; Chavez-Blanco, A.; Medina-Franco, J.L.; Saldivar-Gonzalez, F.; Flores-Torrontegui, Y.; Juarez, M.; Díaz-Chávez, J.; Gonzalez-Fierro, A.; Dueñas-González, A. Ivermectin as an inhibitor of cancer stem-like cells. *Molecular medicine reports*, **2018**, *17*, 3397–3403.

[18] Cioli, D.; Pica-Mattoccia, L. Praziquantel. *Parasitology research*, **2003**, *90 Supp 1*, S3-9.

[19] R. Pax, J. L. Bennett, and R. Fetterer. *A Benzodiazepine Derivative and Praziquantel: Effects on Musculature of Schistosoma mansoni and Schistosoma japonicum;* Springer, **1978**.

[20] Wu, Z.H.; Lu, M.; Hu, L.Y.; Li, X. Praziquantel synergistically enhances paclitaxel efficacy to inhibit cancer cell growth. *PloS one*, **2012**, *7*, e51721.

[21] Fox, L.M.; Saravolatz, L.D. Nitazoxanide: a new thiazolide antiparasitic agent. *Clinical infectious diseases : an official publication of the Infectious Diseases Society of America*, **2005**, *40*, 1173–1180.

[22] Sisson, G.; Goodwin, A.; Raudonikiene, A.; Hughes, N.J.; Mukhopadhyay, A.K.; Berg, D.E.; Hoffman, P.S. Enzymes associated with reductive activation and action of nitazoxanide, nitrofurans, and metronidazole in Helicobacter pylori. *Antimicrobial agents and chemotherapy*, **2002**, *46*, 2116–2123.

[23] Müller, J.; Sidler, D.; Nachbur, U.; Wastling, J.; Brunner, T.; Hemphill, A. Thiazolides inhibit growth and induce glutathione-S-transferase Pi (GSTP1)-dependent cell death in human colon cancer cells. *International journal of cancer*, **2008**, *123*, 1797–1806.

[24] Senkowski, W.; Zhang, X.; Olofsson, M.H.; Isacson, R.; Höglund, U.; Gustafsson, M.; Nygren, P.; Linder, S.; Larsson, R.; Fryknäs, M. Three-Dimensional Cell Culture-Based Screening Identifies the Anthelmintic Drug Nitazoxanide as a Candidate for Treatment of Colorectal Cancer. *Molecular cancer therapeutics*, **2015**, *14*, 1504–1516.

[25] Wang, X.; Shen, C.; Liu, Z.; Peng, F.; Chen, X.; Yang, G.; Zhang, D.; Yin, Z.; Ma, J.; Zheng, Z.; Zhao, B.; Liu, H.; Wang, L.; Wu, J.; Han, D.; Wang, K.; Zhong, C.; Hou, X.; Zhao, W.; Shu, M.; Wang, X.; Zhao, S. Nitazoxanide, an antiprotozoal drug, inhibits late-stage autophagy and promotes ING1-induced cell cycle arrest in glioblastoma. *Cell death & disease*, **2018**, *9*, 1032.

[26] Fan-Minogue, H.; Bodapati, S.; Solow-Cordero, D.; Fan, A.; Paulmurugan, R.; Massoud, T.F.; Felsher, D.W.; Gambhir, S.S. A c-Myc activation sensor-based high-throughput drug screening identifies an antineoplastic effect of nitazoxanide. *Molecular cancer therapeutics*, **2013**, *12*, 1896–1905.

[27] Pourgholami, M.H.; Wangoo, K.T.; Morris, D.L. Albendazole-cyclodextrin complex: enhanced cytotoxicity in ovarian cancer cells. *Anticancer research*, **2008**, *28*, 2775–2779.

[28] Chu, S.W.L.; Badar, S.; Morris, D.L.; Pourgholami, M.H. Potent inhibition of tubulin polymerisation and proliferation of paclitaxel-resistant 1A9PTX22 human ovarian cancer cells by albendazole. *Anticancer research*, **2009**, *29*, 3791–3796.

[29] Noorani, L.; Stenzel, M.; Liang, R.; Pourgholami, M.H.; Morris, D.L. Albumin nanoparticles increase the anticancer efficacy of albendazole in ovarian cancer xenograft model. *Journal of nanobiotechnology*, **2015**, *13*, 25.

[30] Culetto, E.; Baylis, H.A.; Richmond, J.E.; Jones, A.K.; Fleming, J.T.; Squire, M.D.; Lewis, J.A.; Sattelle, D.B. The Caenorhabditis elegans unc-63 gene encodes a levamisole-sensitive nicotinic acetylcholine receptor alpha subunit. *The Journal of biological chemistry*, **2004**, *279*, 42476–42483.

[31] Rayes, D.; Flamini, M.; Hernando, G.; Bouzat, C. Activation of single nicotinic receptor channels from Caenorhabditis elegans muscle. *Molecular pharmacology*, **2007**, *71*, 1407–1415.

[32] Hu, Y.; Xiao, S.-H.; Aroian, R.V. The new anthelmintic tribendimidine is an L-type (levamisole and pyrantel) nicotinic acetylcholine receptor agonist. *PLoS neglected tropical diseases*, **2009**, *3*, e499.

[33] Nageshwari, B.; Merugu, R. Effect of levamisole on expression of CD138 and interleukin-6 in human multiple myeloma cell lines. *Indian journal of cancer*, **2017**, *54*, 566–571.

[34] Qiao, X.; Wang, C.; Wang, W.; Shang, Y.; Li, Y.; Ni, J.; Chen, S.-Z. Levamisole enhances DR4-independent apoptosis induced by TRAIL through inhibiting the activation of JNK in lung cancer. *Life sciences*, **2020**, *257*, 118034.

[35] Hamilton, G.; Rath, B. Repurposing of Anthelminthics as Anticancer Drugs. *Oncomedicine*, **2018**, *3*, 1–8.

[36] Xu, L.; Le Zhang; Hu, C.; Liang, S.; Fei, X.; Yan, N.; Zhang, Y.; Zhang, F. WNT pathway inhibitor pyrvinium pamoate inhibits the self-renewal and metastasis of breast cancer stem cells. *International journal of oncology*, **2016**, *48*, 1175–1186.

[37] Wiegering, A.; Uthe, F.-W.; Hüttenrauch, M.; Mühling, B.; Linnebacher, M.; Krummenast, F.; Germer, C.-T.; Thalheimer, A.; Otto, C. The impact of pyrvinium pamoate on colon cancer cell viability. *International journal of colorectal disease*, **2014**, *29*, 1189–1198.

[38] Deng, L.; Lei, Y.; Liu, R.; Li, J.; Yuan, K.; Li, Y.; Chen, Y.; Liu, Y.; Lu, Y.; Edwards, C.K.; Huang, C.; Wei, Y. Pyrvinium targets autophagy addiction to promote cancer cell death. *Cell death & disease*, **2013**, *4*, e614.

[39] Dattilo, R.; Mottini, C.; Camera, E.; Lamolinara, A.; Auslander, N.; Doglioni, G.; Muscolini, M.; Tang, W.; Planque, M.; Ercolani, C.; Buglioni, S.; Manni, I.; Trisciuoglio, D.; Boe, A.; Grande, S.; Luciani, A.M.; Iezzi, M.; Ciliberto, G.; Ambs, S.; Maria, R. de; Fendt, S.-M.; Ruppin, E.; Cardone, L. Pyrvinium Pamoate Induces Death of Triple-Negative Breast Cancer Stem-Like Cells and Reduces Metastases through Effects on Lipid Anabolism. *Cancer research*, **2020**, *80*, 4087–4102.

[40] Lamb, R.; Ozsvari, B.; Lisanti, C.L.; Tanowitz, H.B.; Howell, A.; Martinez-Outschoorn, U.E.; Sotgia, F.; Lisanti, M.P. Antibiotics that target mitochondria effectively eradicate cancer stem cells, across multiple tumor types: treating cancer like an infectious disease. *Oncotarget*, **2015**, *6*, 4569–4584.

[41] Stockert, J.C.; Trigoso, C.I.; Llorente, A.R.; Del Castillo, P. DNA fluorescence induced by polymethine cation pyrvinium binding. *The Histochemical journal*, **1991**, *23*, 548–552.
